# Genome-Wide Association Mapping in Tomato (*Solanum lycopersicum*) Is Possible Using Genome Admixture of *Solanum lycopersicum* var. *cerasiforme*

**DOI:** 10.1534/g3.112.002667

**Published:** 2012-08-01

**Authors:** Nicolas Ranc, Stephane Muños, Jiaxin Xu, Marie-Christine Le Paslier, Aurélie Chauveau, Rémi Bounon, Sophie Rolland, Jean-Paul Bouchet, Dominique Brunel, Mathilde Causse

**Affiliations:** * INRA, UR1052, Unité de Génétique et Amélioration des Fruits et Légumes, Avignon, 84143, France; † Northwest A&F University, College of Horticulture, Yang Ling, Shaanxin, 712100, People's Republic of China, and; ‡ INRA, UR1279, Unité Etude du Polymorphisme des Génomes Végétaux, CEA-Institut de Génomique-CNG, Evry, 91057, France

**Keywords:** tomato (*Solanum lycopersicum*), admixture, association mapping, linkage disequilibrium

## Abstract

Genome-wide association mapping is an efficient way to identify quantitative trait loci controlling the variation of phenotypes, but the approach suffers severe limitations when one is studying inbred crops like cultivated tomato (*Solanum lycopersicum*). Such crops exhibit low rates of molecular polymorphism and high linkage disequilibrium, which reduces mapping resolution. The cherry type tomato (*S. lycopersicum var. cerasiforme*) genome has been described as an admixture between the cultivated tomato and its wild ancestor, *S. pimpinellifolium*. We have thus taken advantage of the properties of this admixture to improve the resolution of association mapping in tomato. As a proof of concept, we sequenced 81 DNA fragments distributed on chromosome 2 at different distances in a core collection of 90 tomato accessions, including mostly cherry type tomato accessions. The 81 Sequence Tag Sites revealed 352 SNPs and indels. Molecular diversity was greatest for *S. pimpinellifolium* accessions, intermediate for *S. l. cerasiforme* accessions, and lowest for the cultivated group. We assessed the structure of molecular polymorphism and the extent of linkage disequilibrium over genetic and physical distances. Linkage disequilibrium decreased under *r*^2^ = 0.3 within 1 cM, and minimal estimated value (r^2^ = 0.13) was reached within 20 kb over the physical regions studied. Associations between polymorphisms and fruit weight, locule number, and soluble solid content were detected. Several candidate genes and quantitative trait loci previously identified were validated and new associations detected. This study shows the advantages of using a collection of *S. l. cerasiforme* accessions to overcome the low resolution of association mapping in tomato.

Linkage mapping has proved its usefulness in detecting important qualitative and quantitative loci in crops (Doebley *et al.* 1997; Frary *et al.* 2000). Linkage mapping strategies are limited in detecting loci underlying quantitative traits (QTL) because, commonly, only two extreme parents are used for generating the segregating population, and only a few recombination events are studied (Flint-Garcia *et al.* 2005). Furthermore, the discovery of new genes underlying the variation of phenotypic traits is limited to those having a large effect on phenotypic variation (Buckler *et al.* 2002). Genetic resources consist of a large number of accessions with different histories, mutations, and recombination events and may represent a large reservoir of phenotypic and molecular diversity. The association mapping strategy has been proposed to identify polymorphisms involved in phenotypic variations and may prove useful in identifying interesting alleles for breeding purpose.

Recently, the value of association mapping in genetic studies has been described (Gupta *et al.* 2005; Zhu *et al.* 2008). New statistical methods have been developed to analyze structured samples (Pritchard *et al.* 2000b; Price *et al.* 2006; Yu *et al.* 2006), and these methods have been efficiently applied to plants (Thornsberry *et al.* 2001; Flint-Garcia *et al.* 2005; Zhao *et al.* 2007). One of the most important parameters in association mapping is the intensity of linkage disequilibrium (LD) over the genome. LD is defined as nonrandom association of alleles, and its intensity determines the resolution of association mapping (Rafalski 2002). When LD extends within several hundreds of base-pairs (bp), a large number of markers is necessary to cover the whole genome, and alleles at selected candidate genes should be tested for association. If it extends over greater distances, the whole genome may be scanned with a lower density of markers to identify polymorphisms associated with phenotypic variation. The extent of LD over the genome is expected to vary according to the species, genome region, and population under study (Nordborg and Tavare 2002). LD is expected to be stronger in inbred than outbred species because recombination is less effective in selfing species, where individuals are more likely to be homozygous at a given locus, than in outcrossing species (Flint-Garcia *et al.* 2003). Moreover, reduction in population size (bottleneck) increases the drift effect and, consequently, LD within and between chromosomes. Thus, inbred crops are theoretically less suitable for high-resolution association mapping because of their low level of molecular diversity and high overall genomic LD.

The cultivated tomato (*Solanum lycopersicum* var. *esculentum*, formerly *Lycopersicon esculentum*) is a diploid plant that is predominantly selfing and highly inbred. The tomato was domesticated from its wild relative, *S. pimpinellifolium*, with the first domesticated form presumably represented by *S. lycopersicum* var. *cerasiforme* (*i.e.*, the cherry tomato). The modern cultivated tomato accessions exhibit a low level of genetic diversity compared with their wild relatives as the result of several bottlenecks that occurred during domestication, migration, and selection; this low level of genetic diversity is exacerbated by the autogamous nature of this species (Yang *et al.* 2004; Van Deynze *et al.* 2007). As expected, LD extends through long genetic distances in the cultivated accessions (van Berloo *et al.* 2008). Part of the *S. lycopersicum* var. *cerasiforme* (*S. l. cerasiforme*) accessions display a genetic admixture pattern between cultivated and wild tomato accessions (Ranc *et al.* 2008). Such an admixture population could be compared with advanced intercrossed lines (*i.e.*, populations derived from two inbred strains that were randomly intercrossed for several generations). As a consequence, cherry-type tomatoes have a greater level of genetic diversity than *S. l. esculentum* and a greater phenotypic diversity than *S. pimpinellifolium*, which offers interesting properties for association mapping.

Association mapping has rarely been used to identify the molecular bases of QTL in the tomato, with the exception of analysis of two regions encompassing map-based cloned genes. Recently, association mapping was shown to be relevant in identifying quantitative trait nucleotides (QTN) responsible for locule number (LCN) differences between *S. l. cerasiforme* and *S. l. esculentum* (Muños *et al.* 2011). A sequence of 1800 bp containing the QTL *lcn2.1* was identified by map-based cloning. LD mapping detected two SNPs within this sequence that show highly significant associations with phenotypic variation. Previously, Nesbitt and Tanksley (2002) failed to find any association between fruit size and genomic sequence of the *fw2.2* region, which carries a QTL for fruit size; however, they studied only 39 cherry tomato accessions.

The objectives of the present study was to define the optimal conditions for whole-genome association in the tomato by using cherry tomato accessions and to assess the marker density needed to perform association mapping in this crop. This pilot study focused on chromosome 2 because several clusters of QTL for fruit morphology and quality traits have been mapped on this chromosome (Causse *et al.* 2002). Four genes underlying these QTL have been cloned: *fw2.2*, which is responsible for fruit weight (FW) variation (Frary *et al.* 2000); *Ovate*, which causes pear-shaped tomato fruit (Liu *et al.* 2002); *Cnr*, which causes nonripening fruit (Manning *et al.* 2006); and *lcn2.1*, responsible for LCN (Muños *et al.* 2011).

We genotyped a core collection of 90 accessions mainly composed of *S. l. cerasiforme* accessions by Sanger sequencing of DNA fragments. We sequenced 81 fragments mapped on chromosome 2 and spread over three different mapping densities: (1) a whole chromosome density (1 fragment/5 cM); (2) a fine mapping density (1 fragment/cM) and (iii) a physical mapping density (1 fragment/100 kb). For physical mapping density, we focused on regions in which QTL were previously fine mapped (Lecomte *et al.* 2004). In this study, we describe the level of molecular polymorphism detected. The extent of LD was assessed over the entire chromosome and over physical distances. Finally, association tests regarding FW, LCN, and soluble solid content (SSC) phenotypes were performed.

## Materials and Methods

### Plant material

The accessions were sampled in a germplasm collection that is maintained and characterized at the Institut National de la Recherche Agronomique (INRA) in Avignon, France. These accessions are part of a core collection drawn from 380 accessions that maximizes both genetic and phenotypic diversity (Ranc *et al.* 2008). A set of 90 tomato accessions (supporting information, Table S1) was used for sequence analysis. This sample was composed of 63 cherry type tomato accessions (*i.e.*, *S. lycopersicum* var. *cerasiforme*, hereafter named *S. l. cerasiforme*), 17 large fruited accessions (*S. lycopersicum* var. *esculentum*, hereafter named *S. l. esculentum*), and 10 *S. pimpinellifolium* accessions. Accessions were derived from French researchers' prospecting, breeders' collections, the Tomato Genetics Resource Center (Davis, CA), the Centre for Genetic Resources (Wageningen, The Netherlands), the North Central Regional Plant Introduction Station (Ames, IA), and the N.I. Vavilov Research Institute of Plant Industry (St. Petersburg, Russia).

### Tomato phenotyping

The accessions were grown during 2007 and 2008 summers in Avignon. Four plants per accession were bred in plastic greenhouse. Three harvests of 10 ripe fruits were done for each accession and were used as repetition in the phenotypic analysis. The 10 fruits were phenotyped for FW, LCN, and SSC. Year and accession effects were assessed by two-factor analysis of variance with [R] software (R Development Core Team 2005). Heritability estimations were calculated as following: $h_{F}^{2}=\sigma_{g}^{2}/(\sigma_{g}^{2}=\sigma_{e}^{2})$ with $\sigma_{g}^{2}$ and $\sigma_{e}^{2}$ the genetic and residual variance, respectively. $\sigma_{g}^{2}$ and $\sigma_{e}^{2}$were estimated by (MSc-MSe)/89 and MSe, respectively. MSc and MSe represent the mean squares of cultivar and residual effects, respectively. Because genetic effect over the two years was much significantly greater than year effect, we calculated associations by using accession adjusted means over years. FW and LCN were log transformed (Table S1). Pearson correlations were assessed among traits.

### DNA fragments sequenced

**Figure 1:**
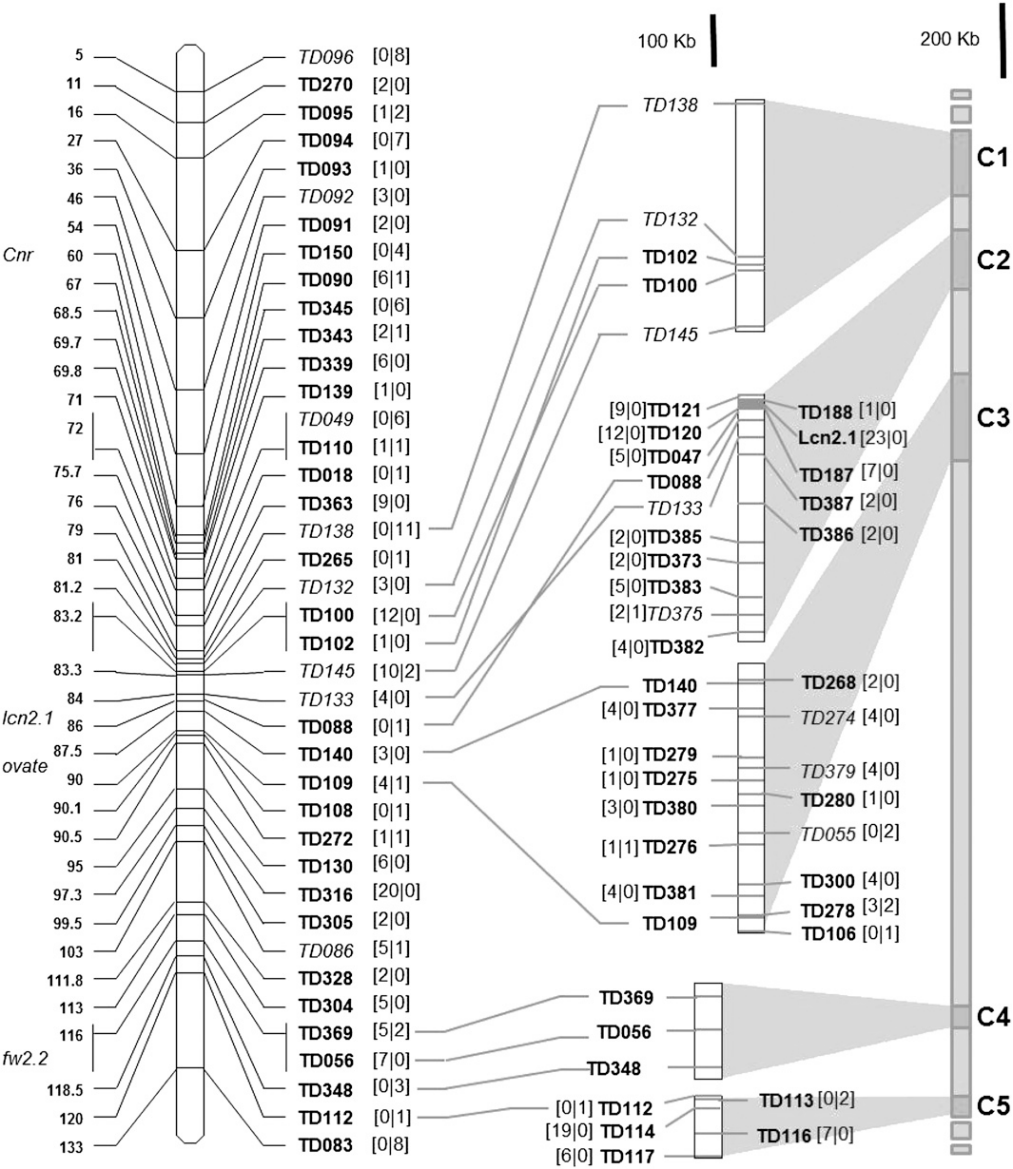
Genetic and physical location of the polymorphic fragments sequenced on chromosome 2. Genetic distances on the EXPEN2000 reference map are indicated on the left of the chromosome. Physical contigs are drawn on the right of the scheme. Cloned QTL are indicated on the left of the chromosome. Gray shaded area indicates homology of contigs on chromosome 2 pseudo-molecule. Numbers of polymorphisms (SNPs and indels) found in noncoding and coding regions are indicated within bracket in the first and second position, respectively. Markers in italics show high LD when compared together.

The positions of the sequence tag sites (STS) along the chromosome 2 are shown in Figure 1. We used Primer3 (Rozen and Skaletsky 2000) to design pairs of primers for each STS based on sequence data of genes and markers mapped on chromosome 2 (http://solgenomics.net/). These fragments were chosen to cover the entire chromosome with three different densities: (1) fragments every 5 cM chosen to cover the whole chromosome (2); fragments every cM chosen to cover the middle of the chromosome; and (3) fragments every 100 Kb chosen to cover five physical contigs representing candidate regions for fruit quality QTL: Contig1 (SL2.40ch02: 41129698.. 41563558), mapped around a sugar content QTL (sugs2.1); Contig2 (SL2.40ch02: 41752714.. 42140082), mapped in an LCN QTL (lcn2.1); Contig3 (SL2.40ch02: 42664935.. 43230501), mapped around a SSC QTL (ssc2.2); Contig4 (SL2.40ch02: 46744832.. 46893523), mapped in around FW QTL (fw2.2); and Contig5 (SL2.40ch02: 47342796.. 47472243), mapped around a sugar content QTL (sugs 2.2). Because of a low level of polymorphism previously described in *S. lycopersicum*, we targeted fragments for sequencing on intronic or intergenic regions. For a specific unigene, intron localization was predicted with tblastx on *Arabidopsis thaliana* genomic sequence and primers were designed on exonic sequence surrounding introns. The characteristics of the STS are presented in Table S2.

### Fragment sequencing and analysis

Genomic DNA was isolated from 100 mg of frozen leaves using the DNeasy Plant Mini Kit (QIAGEN, Valencia, CA) according the manufacturer's recommendations. Amplification reactions were performed in a final volume of 5 µL in a reaction mix composed of 2.5 ng of template DNA, 0.4 pmol of each primer, 0.05 mM concentration of each deoxynucleotide, 2 mM MgSO_4_, 1X *Taq* polymerase buffer P, and 0.03 units of Platinum *Taq* HiFi (Invitrogen, Carlsbad, CA). After 5 min of denaturation at 94°, 30 cycles were performed of 20 s at 94°, initial denaturation during 20 s at 55°, annealing during 2 min at 68°, followed by a final extension step of 5 min at 68°. Pairs of primers revealing single-band polymerase chain reaction (PCR) product were chosen for sequencing. PCR products were purified using the ExoSAP method with Exonuclease I (NEB, Beverly, MA) and Shrimp Alkaline Phosphatase (USB, Cleveland, OH). Fragments were sequenced with SP6 universal primer in an adapted 5-µL reaction volume method using BigDye terminator V3.1 and analyzed on an ABI 3730 xl sequencer (Applied Biosystems, Foster City, CA). Sequence alignment and SNP detection were performed using Genalys software available at http://software.cng.fr/ (Takahashi *et al.* 2003). Sequences of *lcn2.1*, previously obtained for this core collection (Muños *et al.* 2011), were added in this study (embl accession number JF284938 and JF284939). Genotype data are provided in Table S3.

### Linkage disequilibrium

The molecular diversity was estimated by Watterson's θ. The LD parameter *r^2^* was estimated among loci with tassel (Bradbury *et al.* 2007), and the comparison-wise significance was computed by 1000 permutations. We compared different strategies for analyzing LD decay over genetic distances. We examined pairwise LD values, analyzing all polymorphisms with minor allele frequency (MAF) greater than 5% or only one polymorphism by fragment with the greatest heterozygosity index. We also compared pairwise LD decay between polymorphisms assessed in the whole population (N = 90) or only in the *cerasiforme* subset (n = 63). Pairwise *r^2^* were plotted according to genetic distance between two loci, and nonlinear regression fitted the decay of LD over genetic or physical distance. The decrease of LD over genetic distance was fitted by the equation: $y=a=be^{-c/x}$ using nonlinear regression, where y represents *r^2^* and x represents the genetic or physical distance in cM or kb (Tenesa *et al.* 2004).

### Association analysis

An association study was performed with the set of 90 accessions. Several statistical models were tested: (1) the Simple general linear model (GLM); (2) the structured association model (Q model), taking into account only the structure of the collection; and (3) the mixed linear model (K+Q or MLM model), taking into account both kinship and structure, as described by Yu *et al.* (2006). The significance of associations between traits and markers was estimated with Tassel. Population assignment of individuals was inferred by Structure 2.1 software (Pritchard *et al.* 2000a) based either on 20 simple sequence repeat (SSR) markers spread throughout the genome (Ranc *et al.* 2008) or on the genotypes of all the STS markers or just a subset of these markers. For inferring the most likely number of population, the Evanno *et al.* (2005) transformation method was used. The Ritland's matrix of relative kinship coefficients (Ritland 1996), implemented in the mixed linear model, was estimated using SPAGeDi (Hardy and Vekemans 2002) based on the set of SSR markers. According to Yu *et al.* (2006), the diagonal of the matrix was set to 2.0, and the negative values were set to 0. To deal with multiple testing, we computed adjusted *P* values using Benjamini and Hochberg (2000) procedures to control for the false discovery rate. Associations with an adjusted *P* value less than 0.005 were declared significant. For markers that were significantly associated with a trait, a general linear model with all fixed-effect terms was used to estimate R^2^, the amount of phenotypic variation explained by each marker. The standardized effect of each marker was also calculated by dividing the difference of average values of the two homozygous classes by the phenotypic standard deviation for the trait (Weber *et al.* 2008). The accession used for tomato genome sequencing, Heinz 1706, was used as a reference for allele effect calculation.

## Results

### Identification of polymorphisms on chromosome 2

**Figure 2:**
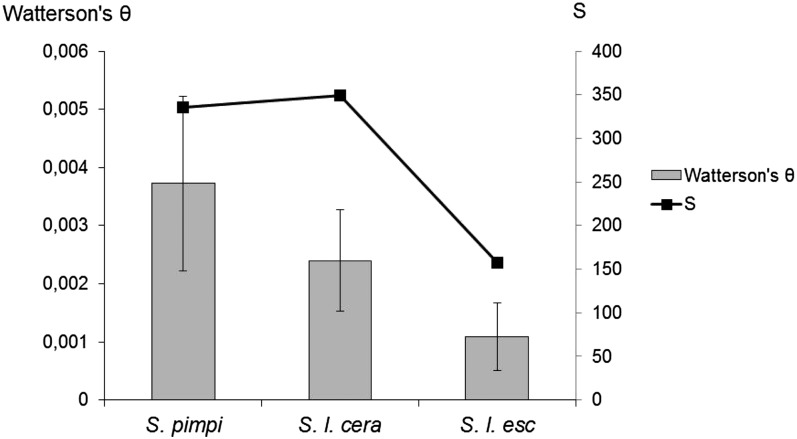
Molecular diversity of the three groups of tomato based on 352 polymorphisms. Molecular diversity was estimated by Watterson's θ and compared with the total number of polymorphisms (S) for S. pimpinellifolium, *S. l. cerasiforme*, and *S. l. esculentum*.

Eighty-six pairs of primers, corresponding to 86 loci on chromosome 2, revealed a unique PCR band and were chosen for forward sense sequencing of the 90 tomato accessions. Five fragments were not readable because of heterozygous signals, probably due to the amplification of paralogous sequences. The 81 remaining fragments (Table S4) had an average size of 542 bp. Noncoding regions represented almost 69% (30,396 bp) of the total length sequenced (44,223 bp). Eleven fragments (13%) were monomorphic among the 90 accessions. Figure 1 shows the location and polymorphism content of the 70 polymorphic STS. A total of 300 single-nucleotide polymorphisms (SNPs) and 52 insertion-deletions (indels) were detected among 90 accessions. Only polymorphisms with MAF values greater than 5% were taken into account in the following description. Polymorphisms were analyzed according to species membership of accessions (Table 1). SNPs and indels were more frequent in noncoding regions, with an average of 8.7 polymorphisms per 1000 bp, than in the exonic parts of genes (average of 5.4 polymorphisms per 1000 bp). The molecular diversity decreased from wild to cultivated groups, whereas the number of polymorphisms dropped only for *S. l. esculentum* (Figure 2). *S. l. cerasiforme* shared polymorphisms with both cultivated and wild accessions. *S. l. cerasiforme* had only five specific polymorphisms, and 344 polymorphisms shared with one of the two other species (187 with *S. pimpinellifolium*, 11 with *S. lycopersicum*, and 146 with both species). Fifty-four percent of overall polymorphisms identified in *S. l. esculentum* corresponded to singletons within this group. Most of these polymorphisms were carried by two accessions (LA0409 and Stupicke Polni Rane).

**Table 1:** Distribution and frequencies of polymorphisms (SNP and indel) across species and ratio of polymorphism in coding and noncoding region

	Number of Access.	Number of Total Polymorphic Sites	Number of Shared Polymorph.*^a^*			Polymorph. Frequency for 1000 bp		Noncoding/Coding Polymorphisms Ratio
			*esc*	*cera*	*pimpi*	coding	noncoding	
*esc*	17	157	0			1.66	4.27	2.57
*cera*	63	349	11	5		5.42	8.61	1.59
*pimpi*	10	336	0	187	3	5.27	8.25	1.57

All fragments (81) are taken into account.

a Numbers in diagonal indicate species specific polymorphisms.

**Figure 3:**
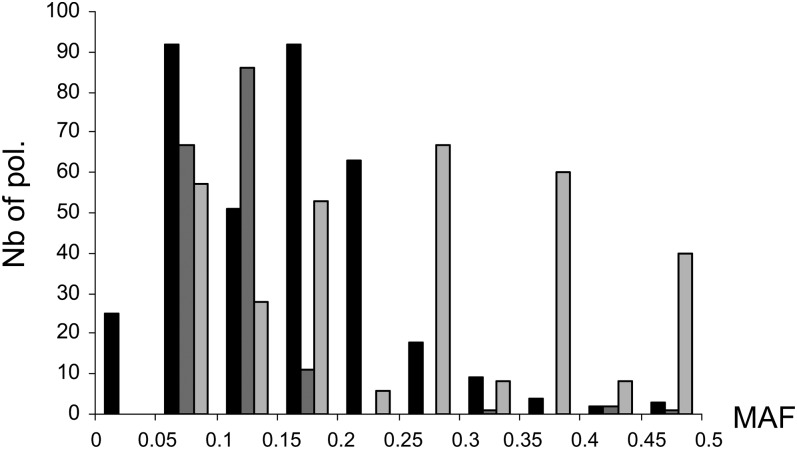
Distribution of polymorphism MAFs among tomato species. *S. l. cerasiforme* (n = 63) is represented in black, *S. l. esculentum* (n = 17) in dark gray, and *S. pimpinellifolium* (n = 10) in light gray. Polymorphisms with overall species MAF lower than 0.05 were previously discarded (see *Materials and Methods*).

The ratio of polymorphisms in noncoding regions to coding regions is similar in *S. pimpinellifolium* and *S. l. cerasiforme* but is strikingly higher in *S. l. esculentum* (Table 1). *S. l. esculentum* also showed an excess of low frequency polymorphisms, as did *S. l. cerasiforme*, although to a lesser extent (Figure 3).

### Linkage disequilibrium

**Figure 4:**
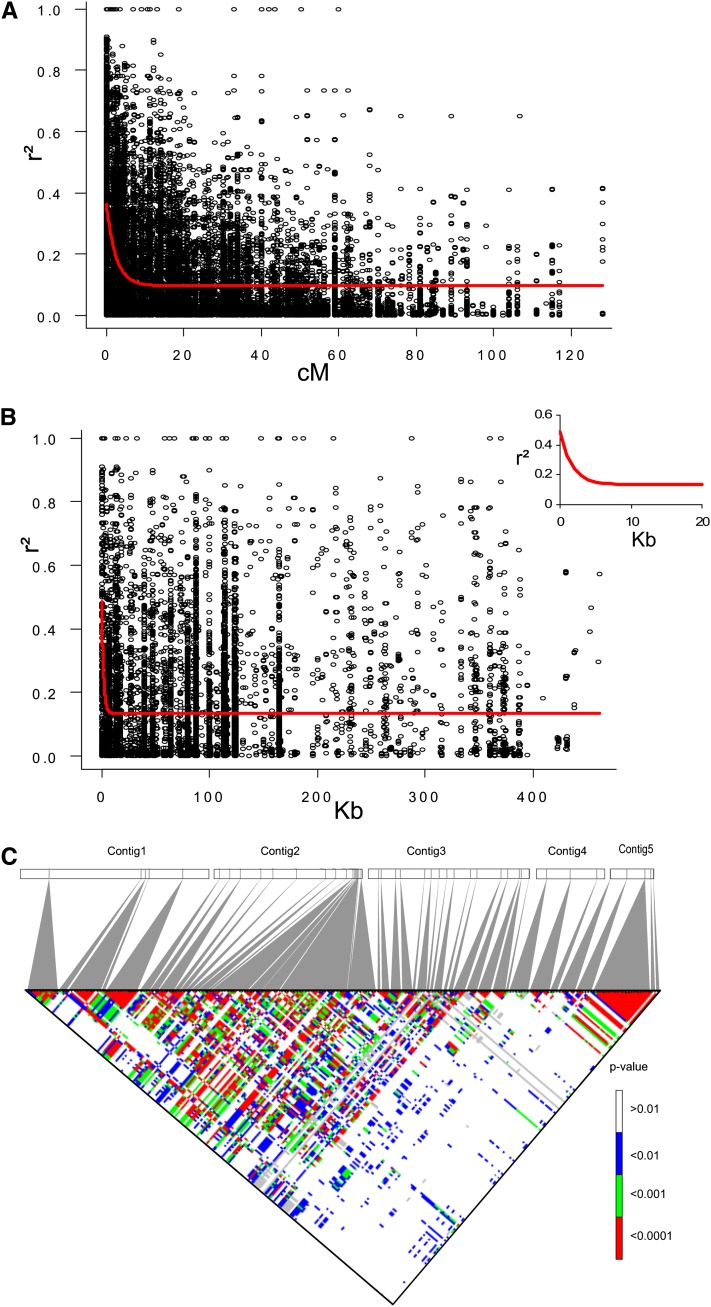
Estimates of LD (*r^2^*) *vs.* genetic and physical distance on chromosome 2 for the 63 *S. l. cerasiforme* accessions. Only polymorphic sites having MAF greater than 5% are indicated (see *Materials and Methods*). (A) Decay of *r^2^* over genetic distance on chromosome 2. Plot of *r^2^* over distance was fitted by nonlinear regression (red curve). (B) Decay of *r^2^* over physical distance on the five major contigs. Plot of *r^2^* over distance is fitted by nonlinear regression (red curve). The inset shows a more detailed view of the LD decay curve for markers located less than 20 Kb apart. (C) Matrix of pairwise LD *P* value between and within physical contigs. *P* values were calculated with 1000 permutations.

We compared LD decay over genetic distances in different samples. LD decreased over shorter genetic distances when all polymorphisms per sequence were taken into account than when using a single polymorphism per fragment. Minimal difference was observed when only the *cerasiforme* subset was analyzed (Figure S1). LD was likely overestimated in the whole sample because of the genetic structure with both cultivated and wild accessions added to the *cerasiforme* subset. For further LD analysis, we focused on the 63 *cerasiforme* accessions. Based on the regression of LD over distances, LD decay reached *r^2^* = 0.3 for a genetic distance of 1 cM, and the minimal value of *r^2^* = 0.09 was obtained for distances of 13 cM (Figure 4A). Nevertheless, high *r^2^* (reaching the maximum value *r^2^* = 1) remained even within a distance of 60 cM, but only 28 sites of 340 (corresponding to 12 STS spread over chromosome 2) were responsible for these high pairwise LD values.

We assessed the extent of LD over physical distances within the five physical contigs covering a total of 1.86 Mb (Figure 4B). The minimal estimated *r^2^* fitted value of 0.13 was obtained within 20 kb, but high pairwise LD persisted within 400 kb. Figure 4C shows the matrix of LD between polymorphic sites of the physical contigs. The pattern of LD intensity over physical distances was heterogeneous. In Contig1, polymorphisms within STS formed blocks with high LD. In Contig2 and the first part of Contig3, STS did not form LD blocks. High LD between and within STS was interrupted by polymorphisms showing low LD with other polymorphisms. A striking break in the LD pattern over physical distance appeared in the middle of Contig3, where strong intrafragment blocks of LD but low LD between fragments were observed. To check whether this region corresponds to a hotspot of recombination, we used the tomato genome sequence to assess the physical positions of STS and the reference genetic map (EXPEN2000, http://solgenomics.net), and we calculated the ratio of physical to genetic distances among STS. The genetic *vs.* physical distance ratios in Contig3 were unevenly distributed with 136 kb/cM between TD140 and TD055 and 20 kb/cM between TD109 and TD106, suggesting the presence of a hotspot of recombination. The difference in LD behavior between and within contig clearly appears on graphical haplotypes (Figure S2).

### Association mapping

The genetic structure of 90 tomato accessions was first estimated using 20 SSR markers spread over the genome. The most probable number of subpopulations in the sample was two (Figure S3). A subdivision in four populations was also detected, as previously shown with 318 accessions (Ranc *et al.* 2008). Twenty-six cherry tomato accessions were not clustered with high probability (Q > 0.8) within one structure group and were thus classified as an admixture between the two major groups (Table S1). The same trend of structure with only two populations was observed when estimating the structure with all the STS markers on chromosome 2.

**Figure 5:**
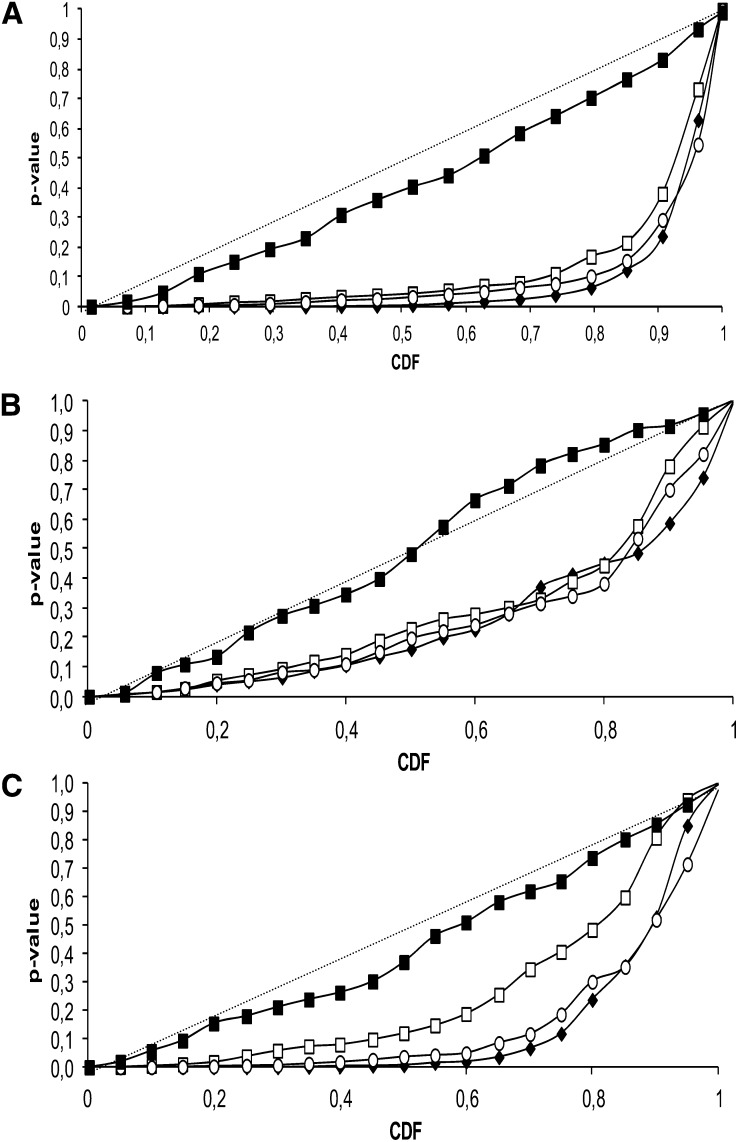
Cumulative density functions (CDF) using several alternative models of association. Model comparisons are performed for FW (A), LCN (B), and SSC (C). Associations are tested for all polymorphic sites with MAF >5% on 90 individuals. Naive GLM (black diamond) and K+Q models, with structure based on SSR markers (white squares), on 4 PCA axis (white circles) and on all STS markers (black squares) were tested. The diagonal indicates uniform distribution of *P* values under the expectation that random SNPs are unlinked to the polymorphisms controlling these traits (H0: no SNP effect).

FW and LCN were log-transformed to fit a normal distribution graphically, but LCN fitted a Poisson distribution. The three traits were correlated together (Figure S4). Broad-sense heritabilities were high: 0.94, 0.96, and 0.95 for SSC, FW and LCN, respectively. Genetic structure assessed by SSR markers had a significant effect on FW and SSC with R^2^ values of 0.24 and 0.12, respectively, whereas population structure accounted only for 5% of the LCN variation. For association mapping, the mixed model taking into account both genetic structure assessed with all STS (Q_STS_) and coancestry matrices (K+Q model) resulted in the best approximation of the expected cumulative distribution of *P* values, followed by the K+Q model with Q assessed with SSR markers (Q_SSR_), then the structured association model (Q model) and the simple model (GLM; Figure 5).

We also tested alternative models to take the structure into account. Taking in the MLM model the four main coordinates of significant axes of principal components analysis provided almost similar results to the naïve model (Figure 5) as well as using k = 4 structure model (data not shown). When we used Q_STS_ in the MLM model, the probability plot was much closer to the diagonal (Figure 5), suggesting that the correction for the structure was much better, and thus we present the associations obtained with this model with corrected *P* values less than 0.05 (Table 2). With this model, we detected 14, 3, and 3 associations with FW, LCN, and SSC, respectively (Table 2). Using just a subset of 265, we found that STS avoiding the loci involved in the main regions where significant associations were detected provided the same results (data not shown).

**Table 2:** Significant associations for fruit weight (FW), locule number (LCN), and soluble solid content (SSC) estimated with K+Q models on 90 accessions

Trait	Locus	Location*^a^*	Model A				MAF*^e^*	Model B
			*P* Value	Corrected *P* Value*^b^*	R^2^*^c^*	a*^d^*		Corrected *P* Value*^b^*
log(FW)	TD091-415	54cM	0.0012	0.004	0.10	10.0	0.18	ns
log(FW)	TD091-607	54cM	8.12×10^−04^	0.003	0.10	9.2	0.24	ns
log(FW)	TD049-528	72cM	6.04×10^−04^	0.002	0.11	9.5	0.48	ns
log(FW)	TD363-213	76cM	0.0019	0.005	0.07	9.6	0.39	ns
log(FW)	TD383-419	84cM-c2.13	7.56×10^−04^	0.003	0.12	12.1	0.11	ns
log(FW)	TD383-558	84cM-c2.13	6.36×10^−04^	0.002	0.13	11.3	0.13	ns
log(FW)	TD383-60	84cM-c2.13	6.36×10^−04^	0.002	0.13	11.3	0.13	ns
log(FW)	TD375-573	84cM-c2.14	0.0011	0.003	0.10	9.0	0.25	ns
log(FW)	TD133-115	84cM-c2.8	3.34×10^−04^	0.002	0.09	7.2	0.33	ns
log(FW)	TD133-395	84cM-c2.8	5.57×10^−04^	0.002	0.09	7.3	0.33	ns
log(FW)	TD387-452	84cM-c2.9	9.40×10^−07^	4.14×10^−05^	0.19	11.6	0.27	0.025
log(FW)	lcn2.1-686	86cM-c2.3	2.86×10^−05^	0.001	0.12	−11.7	0.38	ns
log(FW)	lcn2.1-692	86cM-c2.3	8.95×10^−06^	2.63×10^−04^	0.15	−12.7	0.37	ns
log(FW)	TD274-17	87.5cM-c3.13	9.32×10^−04^	0.003	0.08	8.9	0.26	ns
log(FW)	TD274-325	87.5cM-c3.13	4.76×10^−04^	0.002	0.10	9.8	0.23	ns
log(FW)	TD377-96	87.5cM-c3.14	0.0014	0.004	0.09	8.3	0.17	ns
log(FW)	TD377-97	87.5cM-c3.14	0.0023	0.005	0.08	8.5	0.16	ns
log(FW)	TD377-98	87.5cM-c3.14	0.0014	0.004	0.09	8.3	0.17	ns
log(FW)	TD377-91	87.5cM-c3.14	0.0013	0.004	0.09	8.2	0.17	ns
log(FW)	TD379-326	88cM-c3.11	4.42×10^−04^	0.002	0.12	14.4	0.15	0.001
log(FW)	TD380-256	89cM-c3.8	3.04×10^−04^	0.002	0.11	9.5	0.21	ns
log(FW)	TD380-526	89cM-c3.8	6.13×10^−08^	5.39×10^−06^	0.22	13.2	0.36	0.002
log(FW)	TD280-328	89cM-c3.9	4.54×10^−04^	0.002	0.10	10.5	0.48	ns
log(FW)	TD055-469	89.5cM-c3.7	9.46×10^−05^	0.001	0.13	8.3	0.26	ns
log(FW)	TD278-267	90cM-c3.3	1.73×10^−04^	0.002	0.11	12.0	0.21	0.023
log(FW)	TD278-21	90cM-c3.3	0.003 ns	0.02 ns	—	—	—	0.048
log(FW)	TD278-39	90cM-c3.3	5.23×10^−04^	0.002	0.10	15.0	0.15	0.030
log(FW)	TD278-444	90cM-c3.3	2.30×10^−04^	0.002	0.12	12.4	0.22	0.025
log(FW)	TD278-524	90cM-c3.3	3.81×10^−04^	0.002	0.12	11.9	0.20	0.030
log(FW)	TD300-257	90cM-c3.5	1.95×10^−04^	0.002	0.12	11.6	0.20	ns
log(FW)	TD300-41	90cM-c3.5	0.0011	0.003	0.11	9.2	0.33	ns
log(FW)	TD108-347	90.1cM	8.29×10^−04^	0.003	0.10	7.4	0.27	ns
log(FW)	TD056-134	116cM-c4.7	3.49×10^−04^	0.002	0.12	10.8	0.35	ns
log(FW)	TD369-493	116cM-c4.8	0.0025	0.005	0.09	11.1	0.26	ns
log(FW)	TD116-707	120cM-c4.3	4.90×10^−05^	0.001	0.16	8.1	0.45	0.023
log(FW)	TD117-164	120cM-c4.4	1.16×10^−04^	0.001	0.15	10.1	0.33	ns
log(FW)	TD117-176	120cM-c4.4	1.16×10^−04^	0.001	0.15	10.1	0.33	0.029
log(FW)	TD083-246	133cM	0.0013	0.004	0.09	10.3	0.48	0.033
log(LCN)	TD373-391	86cM-c2.12	2.14×10^−05^	0.002	0.21	−0.68	0.49	0.037
log(LCN)	lcn2.1-692	86cM-c2.3	5.93×10^−13^	1.85×10^−10^	0.44	−1.16	0.37	4.57×10^−09^
log(LCN)	lcn2.1-686	86cM-c2.3	5.32×10^−12^	8.30×10^−10^	0.44	−1.21	0.38	1.34×10^−08^
SSC	TD133-115	84cM-c2.8	1.87×10^−05^	7.12×10^−04^	0.16	−0.63	0.33	ns
SSC	TD133-395	84cM-c2.8	4.90×10^−05^	0.002	0.15	−0.58	0.33	ns
SSC	TD387-452	84cM-c2.9	3.88×10^−07^	5.89×10^−05^	0.24	−0.86	0.27	0.018
SSC	TD047-274	86cM-c2.5	3.96×10^−06^	2.01×10^−04^	0.19	−1.00	0.12	ns
SSC	TD120-212	86cM-c2.6	3.10×10^−04^	0.004	0.13	−0.58	0.33	ns
SSC	TD120-88	86cM-c2.6	2.22×10^−04^	0.003	0.13	−0.59	0.32	ns
SSC	TD140-180	87.5cM-c3.15	1.90×10^−04^	0.003	0.14	−0.73	0.21	ns
SSC	TD379-326	88cM-c3.11	0.008 ns	0.04 ns	—	—	—	0.045
SSC	TD380-256	89cM-c3.8	2.57×10^−04^	0.003	0.13	−0.65	0.21	ns
SSC	TD380-526	89cM-c3.8	1.27×10^−06^	9.68×10^−05^	0.21	−0.70	0.36	0.022
SSC	TD280-328	89cM-c3.9	1.64×10^−04^	0.003	0.14	−0.55	0.48	ns
SSC	TD055-469	89.5cM-c3.7	8.93×10^−05^	0.002	0.15	−0.67	0.26	ns
SSC	TD117-164	120cM-c4.4	1.52×10^−04^	0.003	0.14	−0.70	0.33	ns
SSC	TD117-176	120cM-c4.4	1.52×10^−04^	0.003	0.14	−0.70	0.33	ns

Model A: MLM model, with structure based on 20 SSR (only *P* values less than 0.005 are shown with indication on allele effect); model B: MLM model with structure based on all STS loci on chromosome 2 (*P* values less than 0.05 are shown). MAF, minimal allele frequencies; ns, nonsignificant.

a Nomenclature for the location is as follows: “genetic distance on expen2000 reference map”-“the number of contig”.”the fragment number on this contig”.

b *P* values are corrected following the Benjamini & Hochberg (2000) procedure (see *Materials and Methods*).

c R^2^ were calculated using Q model.

d Allele effects are indicated in grams for FW, mean number of locule for LCN, and °brix for SSC.

e MAFs are shown for each polymorphism.

Because the correction for structure when using Q_SSR_ was not fully satisfying with this model and many associations appeared significant, we only retained the most significant associations with adjusted *P* value lower than 0.005, although some association may still be false positive. With this model, we detected 37, 3, and 14 associations with FW, LCN, and SSC, respectively. Finally, taking into account both STS and SSR markers for the structure analysis gave the same results as the STS alone. For FW, LCN, and SSC, polymorphisms with the greatest *P* values explained a large part of the trait variation (22%, 44%, and 21%, respectively). As reference alleles are based on Heinz 1706, a genotype with large fruit and low SSC, allele effects were almost all positive for FW, whereas allele effects for SSC were all negative.

**Figure 6:**
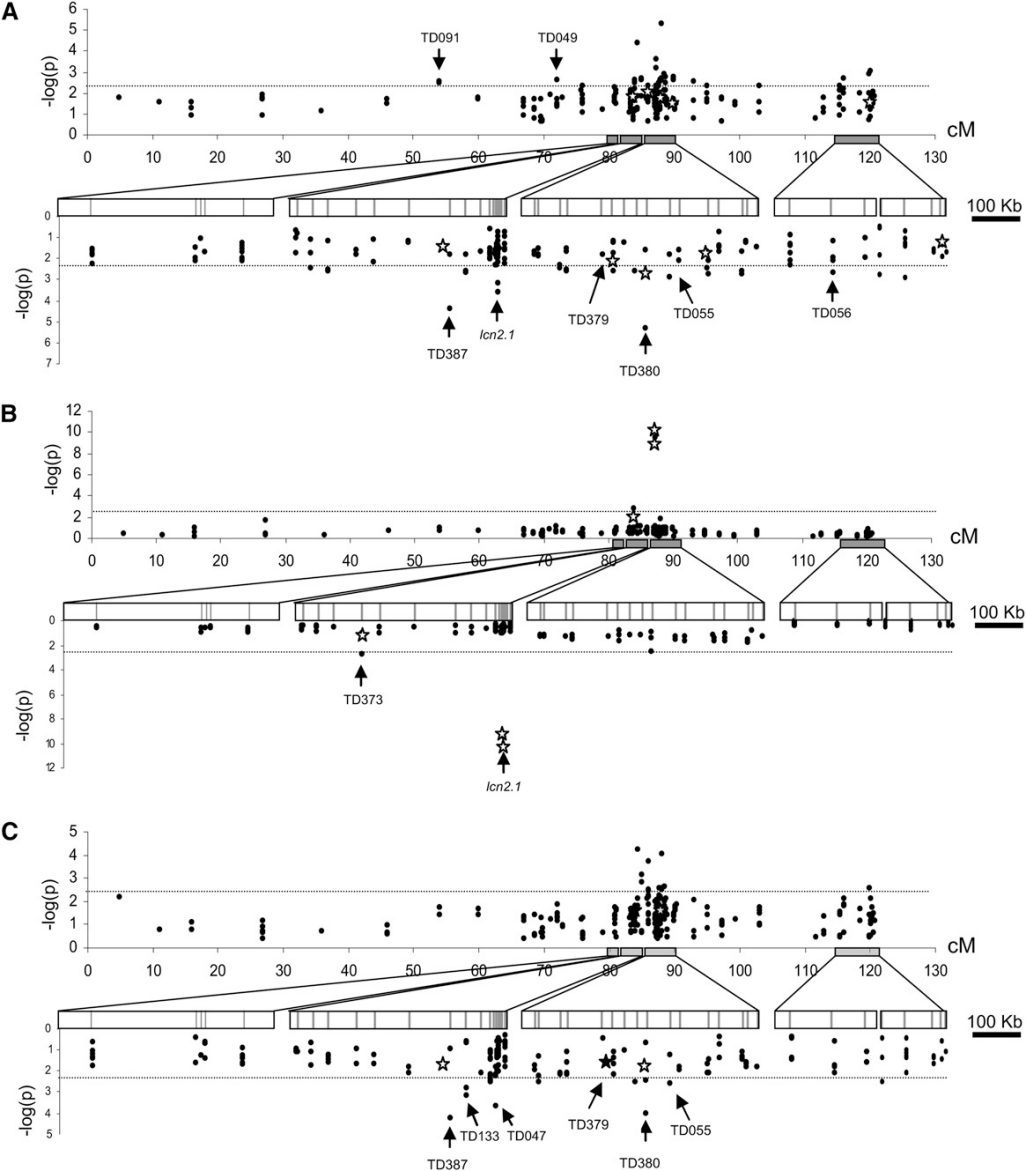
Plot of association *P* values over the chromosome 2. Associations are estimated for 90 accessions. K+Q model was used to screen for association between polymorphisms and (A) FW, (B) LCN, and (C) SSC. Stars indicate the associations detected with the structure assessed with all STS, and black dots the associations detected with 20 SSR markers. The upper part of each graph represents associations along genetic distance over the entire chromosome 2. The lower part shows associations for each physical contig. Arrows indicate the marker name of the most significant associations. Adjusted *P* values for multiple testing (see *Materials and Methods*) are shown.

Figure 6 shows the significant associations between the polymorphisms and the traits along the chromosome with both MLM models. Most of the polymorphisms found in association with one of the traits were part of a dense chromosome region. For FW, the two strongest associations involved TD380-526 (fragment TD380 polymorphic site at position 526) on Contig3 and TD387-452 on Contig2. The *r^2^* value for LD estimation between these two SNPs is 0.41 in the whole accession sample (Figure S5). Because other sites revealed similar level of LD and did not result in significant association, these two associations could correspond to two linked QTL on adjacent contigs. When only the 63 *S. l. cerasiforme* accessions were used for association analysis, TD387-452 was not associated with FW, but association with TD380-526 remained significant. A significant association for FW was detected with TD056-134, which corresponds to the 5′ region of the *fw2.2* QTL previously cloned by positional cloning (Nesbitt and Tanksley 2002). In addition, TD049-528 was associated with FW and colocated with FW2.1, a QTL for FW variation fine mapped in a biparental *S. l. esculentum* × *S. l. cerasiforme* progeny (Lecomte *et al.* 2004). Finally, we detected significant associations for FW with coding polymorphisms in the TD055 fragment corresponding to the *Ovate* gene.

For LCN, only three associations were significant (Table 2, Figure 6B). The greatest associations involved two SNPs that have been identified through map-based cloning as responsible for the LCN variation (Muños *et al.* 2011). LD between these two SNPs was extreme (*r^2^* = 0.95). The other significant association implicated TD373-391 on the same contig. TD373-391 showed the greatest *r^2^* with the *lcn2.1* SNPs (*r^2^* = 0.47). This association may thus result from the LD with the functional *lcn*2.1 SNPs (Figure S5).

For SSC, the strongest associations were found with TD380-526 and TD387-452 loci, which were also significantly associated with FW. These results could be a consequence of the high negative correlation between FW and SSC (r = −0.66). Several of the other polymorphisms showing associations when using QSSR were in significant LD (Figure S5).

When we screened for associations using only the 63 cherry tomato accessions, a group of accession chosen to limit the population structure (Ranc *et al.* 2008), the MLM model was very close to the naïve GLM model (Figure S5). Many associations were no more significant whatever the model. For FW, significant associations were detected with loci that were also detected in the whole collection, TD380-526, TD056-134 (fw2.2), TD116-707, and TD117-219. A new association was detected with TD138-61. For LCN, the two SNPs in the *lcn2.1* locus remained significant. For SSC, the main association with TD380-526 was significant, as well as two with TD120 markers (Table S5).

## Discussion

To assess the genetic diversity among tomato accessions and analyze the extent of LD, we sequenced 81 DNA fragments, covering 44 kb, in 90 accessions of wild and cultivated tomatoes. We detected 352 polymorphic loci (SNP or indel). The extent of LD varied according to the regions, scales, and associations between phenotypes and polymorphisms that were successfully detected.

### Power of *S. l. cerasiforme* for polymorphism discovery

The 63 *S. l. cerasiforme* accessions were previously sampled to maximize both genetic and phenotypic diversity. This sample captures 98% of SSR alleles identified in a larger sample of 144 cherry type accessions (Ranc *et al.* 2008). These accessions represent a large level of molecular variability that is almost identical to that of their wild progenitor, *S. pimpinellifolium*. In tomato, several studies were aimed at discovering SNPs and indels. Nesbitt and Tanksley (2002) searched for molecular polymorphisms in the *fw2.2* region within a collection of *S. l. esculentum* (N = 4) *and S. l. cerasiforme* (N = 39) accessions. They found only one SNP per 7 kbp within *S. l. esculentum* accessions and one SNP per 340 bp within the *S. l. cerasiforme* sample. Mining or resequencing ESTs is another strategy to discover SNPs. Using this method, Yang *et al.* (Yang *et al.* 2004) detected one SNP every 8500 bp in coding regions. Jimenez-Gomez and Maloof (2009) found more than 15,000 intraspecific polymorphisms in a set of 223,000 ESTs in *S. lycopersicum*. However, most of these polymorphisms have low allelic frequency in cultivated tomato. (Labate and Baldo 2005) reported a greater amount of polymorphic ESTs, but the studied accessions were described as highly variable compared with other *S. lycopersicum* accessions because of introgressions from wild relatives. Among the 1487 SNPs detected by Labate *et al.* (2009), only 162 were polymorphic in *S. lycopersicum* breeding germplasm, and most of them had minor allele frequency below 10%.

Van Deynze *et al.* (2007) increased the frequency of SNPs and indels compared with previous studies by focusing on gene introns. In the present study, the use of *S. l. cerasiforme* allowed us to detect 352 polymorphisms (SNP and indels) in 81 sequenced fragments. Four of the eleven monomorphic fragments (TD085, TD098, TD111, and TD384) contained only coding regions, which are less polymorphic. The difference in the polymorphism rate between species for noncoding regions may be a consequence of either (1) hitch-hiking of the region surrounding a selected polymorphism or (2) a demographic bottleneck during domestication associated with a reduction of the population effective size. *S. l. cerasiforme* suffered a decrease of its population effective size during domestication from *S. pimpinellifolium* (Bai and Lindhout 2007). The lack of diversity differences between *S. pimpinellifolium* and *S. l. cerasiforme* could be due to the greater number of accessions sequenced for the latter. The theta diversity statistic corrected for the unbalance in sample size and highlighted a higher molecular diversity in the wild sample. Molecular polymorphism is linked to the population effective size by the Watterson's estimate of the scaled mutation rate (per site) θ=4Neµ, where *Ne* is the population size and *µ* is the mutation rate. The transfer of the tomato from Mexico to Europe during the 16th century greatly reduced the effective population size of the tomato and subsequently decreased the amount of molecular diversity in *S. lycopersicum*. A selection pressure that targeted coding regions could explain the higher ratio between noncoding and coding polymorphisms for *S. lycopersicum*. The reduction of diversity could arise on the fragment targeted by selection but also on the region suffering genetic hitchhiking or background selection (Innan and Stephan 2003). Thus, a less drastic reduction in population size and continuous inter-mating with *S. pimpinellifolium* shaped a higher level of molecular diversity for *S. l. cerasiforme*.

### LD decay over genetic and physical distances

An ancient admixture increased the polymorphism level of cherry tomatoes and limited their overall LD. We reached minimal LD values (*r^2^* < 0.09) with distances greater than 13 cM, but extreme LD values were still found over 60 cM for a few marker pairs. Our results support those from van Berloo *et al.* (2008), who described an LD extent ranging from 15 to 20 cM using AFLP markers in a cherry tomato sample (N = 18). Nesbitt and Tanksley (2002) showed that LD in *S. l. cerasiforme* could be broken within 150 kb around *fw2.2*. With an average ratio of 750 kb/cM on the whole tomato genome (Tanksley *et al.* 1992), the results of LD decay over physical and genetic distances are not consistent. In our *S. l. cerasiforme* sample, some *r^2^* values were still extreme over hundreds of kb, but the drop estimated by nonlinear regression indicated that minimal LD is reached over 20 kb. *Arabidopsis thaliana* also showed a large extent of LD over the *FRI* locus. LD extends to 200 kb, corresponding to one cM in this species (Nordborg *et al.* 2002). This estimate is locus-specific and when studies are performed on the whole genome, LD decays within 10 kb on average (Kim *et al.* 2007). Nordborg (2000) estimated from simulations that LD should vanish over a scale of 10 kb for inbred species. Our results in the cherry tomato support these simulations. The results of LD decay over genetic distances in the tomato are similar to the LD pattern assessed in barley, another highly inbred crop (Zhang *et al.* 2009). In barley, large differences are observed in the LD decay pattern among cultivated accessions, landraces, and wild accessions (Caldwell *et al.* 2006). The greater LD extent for the crop compared with the wild ancestor or to *A. thaliana* could be due to a major bottleneck that fixed large haplotypes during domestication.

The LD pattern observed in the physical contigs is similar to haplotype blocks described in soybean landraces, which is also an inbred crop (Hyten *et al.* 2007). It is also similar to haplotype blocks in *A. thaliana* (Kim *et al.* 2007) and in humans (Daly *et al.* 2001). For the tomato, this LD pattern could be due to recent mutations with low frequencies (more than 50% of polymorphisms had MAF < 0.2). These polymorphism patterns may have evolved by lineage effects rather than by recombination and thus may decay in LD in a small region that is not correlated to distance. The high LD pattern described in the first part of Contig3 and Contig2 could have been shaped by selection. Clusters of QTL have been mapped in this region regarding LCN, fruit shape, FW, soluble solids, and sugar content (Lecomte *et al.* 2004). The selection of new advantageous mutations during domestication should have increased LD in domesticated accessions (Nordborg and Tavare 2002). In *A. thaliana*, LD blocks surrounding selected polymorphisms are significantly longer than blocks surrounding nonselected alleles (Kim *et al.* 2007). Finally, a recombination hotspot is likely responsible for the break in the LD pattern observed in Contig3. Mapping data offered direct confirmation of uneven distribution of recombination over Contig3, but the high density of polymorphisms detected in this study should be mapped on a large F2 population to confirm the presence of such a recombination hotspot (Drouaud 2006).

### Candidate genes are validated by association mapping

Our approach using a core collection was efficient in detecting association in several candidate gene regions. Recently, *lcn2.1* was identified by the map-based cloning approach as a QTN responsible for variation in the tomato LCN (Muños *et al.* 2011). We used information on *lcn2.1* to highlight any possible effect of these two SNPs on FW and SSC. A significant association was found between these two SNPs and FW. Muños *et al.* (2011) highlighted the role of this locus in tomato domestication and further FW increase. This association was the only one with negative allelic effect. The reference genotype, Heinz 1706, has large fruits with only two locules, whereas almost all other two-locule genotypes carry small fruit. The large number of these small-fruit accessions in the reference group induced a negative effect for FW. Nesbitt and Tanksley (2002) could not detect any association in a *S. l. cerasiforme* sample between FW and polymorphisms in the *fw2.2* region cloned previously. These authors concluded that genes other than *fw2.2* are responsible for the variation of FW in cherry tomatoes. The number of accessions (39 *S. l. cerasiforme*, 4 *S. l. esculentum* and 3 *S. pimpinellifolium*) was the principal limitation of the study. Using 90 accessions selected to represent the diversity of a larger collection, we found a significant association with a polymorphic site located in the promoter of the gene. This polymorphism could be responsible for the phenotype variation or could be in LD with the responsible one. The entire cloned region should be sequenced and tested for association before concluding.

### Association mapping for the discovery of new QTL and candidate genes

Many QTL related to fruit traits map to chromosome 2 (Causse *et al.* 2002, Labate *et al.*, 2007). These QTL and the QTL that were fine-mapped in the mapping population *S. l. cerasiforme* × S. *l. esculentum* for FW, LCN and SSC (Lecomte *et al.* 2004) were also identified by association mapping. The screening of polymorphisms on chromosome 2 with high-density markers allowed the detection of many new associations and identification of several putative new candidate genes. The number of significant associations found with FW can result from LD caused by strong selection on this phenotype (Bai and Lindhout 2007). TD380-526 showed the most significant association with FW. This fragment STS matched a predicted gene, Solyc02g085390.1.1, which is homologous to *A. thaliana*'s SNF2-like protein (AT5G66750). This gene has been characterized as an ATP-dependent helicase with chromatin remodeling activity. Chromatin remodeling proteins reconfigure protein–DNA interactions that accompany or induce changes in genome activity, such as gene expression (Kaya *et al.* 2001; Verbsky and Richards 2001). The other highly significantly associated fragment, TD387, has homology with a *S. lycopersicum* unigene (SGN-U596069) and matches the *S. lycopersicum* annotation Solyc02g084070.1.1. This gene has no homology with any gene of known function.

Another association was detected for FW with TD049-528. TD049 was tagged in the 3′ region of a gene coding for glyoxalase I (Solyc02g080630.1.1). This gene colocalizes with a QTL for FW variation in a mapping population derived from a *S. l. esculentum* x *S. l. cerasiforme* cross (Saliba-Colombani *et al.* 2001). Because of the putative impact of glyoxalase I protein on plant cell proliferation (Paulus *et al.* 1993), this gene represents a good candidate gene for FW variation. The two polymorphisms most significantly associated with FW were also associated with SSC. This could be due to the dilution effects of soluble sugars and acids according to fruit size (Prudent *et al.*, 2010). The two polymorphisms were no longer statistically associated with SSC when we added the FW effect as a covariate in the K+Q-model. We observed the same result for TD117 (Solyc02g091640.1.1, which codes for an Endoribonuclease E-like protein), which is genetically close to the *fw2.2* gene. The two other strongest associations, TD047 (promoter of Solyc02g083950.1.1, which codes for the WUSCHEL transcription factor) and TD133 (Solyc02g084030, which codes for a methionine sulfoxide reductase), are both located in the same region as TD120. Because TD047 and TD133 are separated by a distance of 2 cM, this region must be enriched in SNPs to locate precisely one or more responsible polymorphisms. TD055 mapped in a SSC QTL (brix2.2) described in the mapping population involving cherry tomato (Saliba-Colombani *et al.* 2001; Lecomte *et al.* 2004). TD055 was designed in the *Ovate* gene and showed association with SSC. *Ovate* is implicated in the modification of fruit shape, but no effect on SSC has yet been reported. This polymorphism could thus be in LD with the responsible polymorphism. SSC also showed significant association with TD140 (Solyc02g085100 0.1.1), which was identified as an aldose-1-epimerase. This enzyme catalyzes the transformation of alpha-D-glucose into beta-D-glucose and participates in glycolysis and gluconeogenesis. The aldose-1-epimerase thus represents a new candidate for SSC variation.

### Optimal conditions for genome-wide association studies in tomato

We highlighted the greater efficiency of the K+Q-model in dealing with type I error rates for association mapping in the tomato. Information on the estimated familial relatedness in our sample did not influence the results for association with FW because most of the false positives are also corrected with genetic structure information. The K+Q-model may prove its power in a sample of increased size as well as broader allelic diversity (Yu *et al.* 2006). A greater number of markers to detect structure may also reveal a more subtle structure. Taking in the MLM model the structure in 4 subgroups did not change the associations, neither using the coordinates of the first four axes of principal components analysis. The departure from the distribution of *P* values under the expectation that random SNPs are not linked to the polymorphisms controlling FW, SSC, and LCN indicates that our analysis did not succeed in correcting for the whole genetic structure. However, the number of polymorphisms tested was too small and nonrandomly spread over the genome. We then decided to focus only on highly significant associations to reduce the acceptance of false-positive associations. When we used in the MLM model the structure based on the STS detected on chromosome 2 (all or a subset excluding the positions with the main effects), the correction was much better (Figure 5), and many associations were no more significant (Table 2), confirming that many associations could be due to the structure. Nevertheless structure based on STS on chromosome 2 may capture a large part of the LD on that chromosome, and thus exclude interesting associations. Furthermore, the traits studied here have strongly evolved from wild to domesticated forms (as shown by the large part of variation explained by the structure). Correcting for the structure may thus hamper the discovery of relevant loci involved in domestication. For these reasons, we presented results of both models.

The core collection may be efficient in detecting polymorphisms with large effects on trait variation, but it will suffer a decrease of statistical power when dealing with low effect variants. A larger collection is necessary to map such genes with low effect. A higher power may be achieved by increasing the sample size rather than by increasing the number of polymorphisms (Long and Langley 1999). The density of markers needed for association analysis is estimated by LD decay over genetic or physical distance (Rafalski 2002). An *r^2^* value of 0.3 indicates a sufficiently strong LD to be useful for association mapping in human studies (Ardlie *et al.* 2002). In *S. l. cerasiforme* accessions, LD estimated values decayed below this value within 1 cM. One SNP per cM could thus be valuable for medium resolution genome-wide association. Nevertheless, many associations may not be detected with such low number of markers as for physical distances, even if extreme LD is still found over hundreds of kb, an estimate of LD decay indicates that LD is minimal after 20 kb. With a genome size of 950 Mb, a minimum set of 48,000 markers would thus be necessary to have a physical resolution for genome-wide association in tomato. This is a minimal number, which should be probably doubled to tag common polymorphisms in all regions. To validate these estimations based on LD, we looked at the number of significant associations for different marker densities. As expected, the number of SNPs in association with traits increased with densification of polymorphisms. Significant associations (*p* < 0.005) were found using a large mapping strategy (1 marker per 5 cM) for FW, but no association was found for LCN or SSC. The density of markers necessary for analysis will thus depend on the trait, the locus targeted, and the population studied. For example, it would not have been possible to physically map the *lcn*2.1 QTN using only LD because these two SNPs are in complete equilibrium with surrounding polymorphisms, except with TD373, which is located on the physical region of *lcn*2.1 (Muños *et al.* 2011).

Our results suggest that genome admixture of *S. l. cerasiforme* provides an interesting source of molecular diversity for the domesticated tomato. The design of our core collection was efficient enough to detect associations in all the candidate regions where QTL have been previously mapped. We highlighted the greater efficiency of the K+Q model in dealing with type I error rate even in a relatively small sample. Association mapping validated the polymorphisms discovered by positional cloning (*lcn2.1* and *fw2.2*) or fine mapping (*fw2.1*). The screening of polymorphisms along chromosome 2 with a high marker density allowed the detection of many new associations that were confirmed in a larger sample. We identified several putative new candidate genes. If we extrapolate our results to the whole genome, at least 50,000 SNPs will be necessary for high-resolution mapping in such a collection and the double would be more realistic to avoid SNP with low MAF. Due to the recent advances in next-generation sequencing technologies, the development of genomic tools (*i.e.* SNP-chip) of high to very high density will allow screening of the whole tomato genome for association with traits of interest.

## 

## Supplementary Material

Supporting Information
