# Supplementary material for: Genome-Wide Association Mapping in Tomato (Solanum lycopersicum) Is Possible Using Genome Admixture of Solanum lycopersicum var. cerasiforme
Source: G3 (Bethesda). 2012 Aug 1;2(8):853–64. doi: 10.1534/g3.112.002667 (PMC3411241; doi:10.1534/g3.112.002667)
Supplement: Supporting Information [file supp_2_8_853__index.html]

Supporting Information 

# Genome-Wide Association Mapping in Tomato (*Solanum lycopersicum*) Is Possible Using Genome Admixture of *Solanum lycopersicum* var. *cerasiforme*

## Supporting Information for Ranc *et al.*, 2012

**Files in this Data Supplement:**

- Supporting Information - Figures S1-S6 and Tables S1-S5 (PDF, 678 KB)
- Figure S1 - Comparison of different models for the analysis of linkage disequilibrium decay over genetic distances (PDF, 86 KB)
- Figure S2 - Graphical haplotypes of 90 accessions for markers located on physical contigs (PDF, 216 KB)
- Figure S3 - Genetic structure determination of the 90 accessions of wild and cultivated tomato (PDF, 82 KB)
- Figure S4 - Distribution and correlation of fruit weight (FW), locule number (LCN) and soluble solid content (SSC) for the 90 accessions (PDF, 160 KB)
- Figure S5 - Matrix of linkage disequilibrium between markers significantly associated with fruit weight (FW), fruit locule number (LCN) and soluble solids content (SSC) (PDF, 90 KB)
- Figure S6 - Cumulative density functions (CDF) using several alternative models of association for fruit weight on the subset of 63 cerasiforme tomato (PDF, 81 KB)
- Table S1 - Accessions used in the association study (PDF, 87 KB)
- Table S2 - Description of the 81 DNA fragments sequenced located on chromosome 2 (PDF, 103 KB)
- Table S4 - Polymorphism information (PDF, 149 KB)
- Table S5 - Significant associations detected when analysing only the 63 cherry tomato accessions with the MLM model involving either the structure based on SSR markers (Qssr) or the STS markers (Qsnp) (PDF, 62 KB)
- Table S3 - Genotype � All SNP (.xlsx, 135 KB)
