# Supplementary material for: Genome-Wide Association Mapping in Tomato (Solanum lycopersicum) Is Possible Using Genome Admixture of Solanum lycopersicum var. cerasiforme
Source: G3 (Bethesda). 2012 Aug 1;2(8):853–64. doi: 10.1534/g3.112.002667 (PMC3411241; doi:10.1534/g3.112.002667)
Supplement: Supporting Information [file supp_2.8.853_002667SI.pdf]

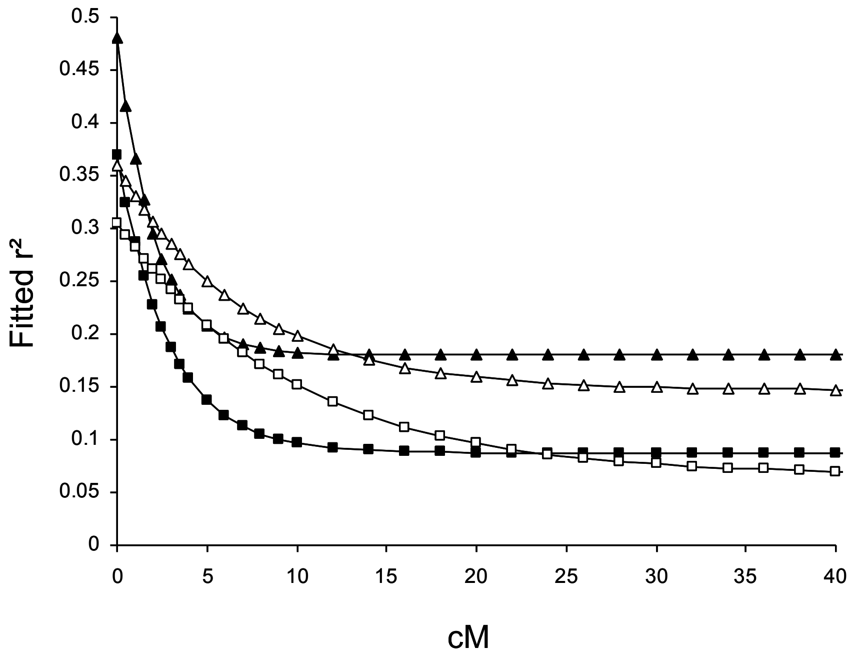

Figure S1 Comparison of different models for the analysis of linkage disequilibrium decay over genetic distances. Whole collection and *S. l. cerasiforme* collection are represented by triangle and square, respectively. One polymorphic site per fragment and all sites with MAF>5% survey strategies are represented in white and black, respectively.

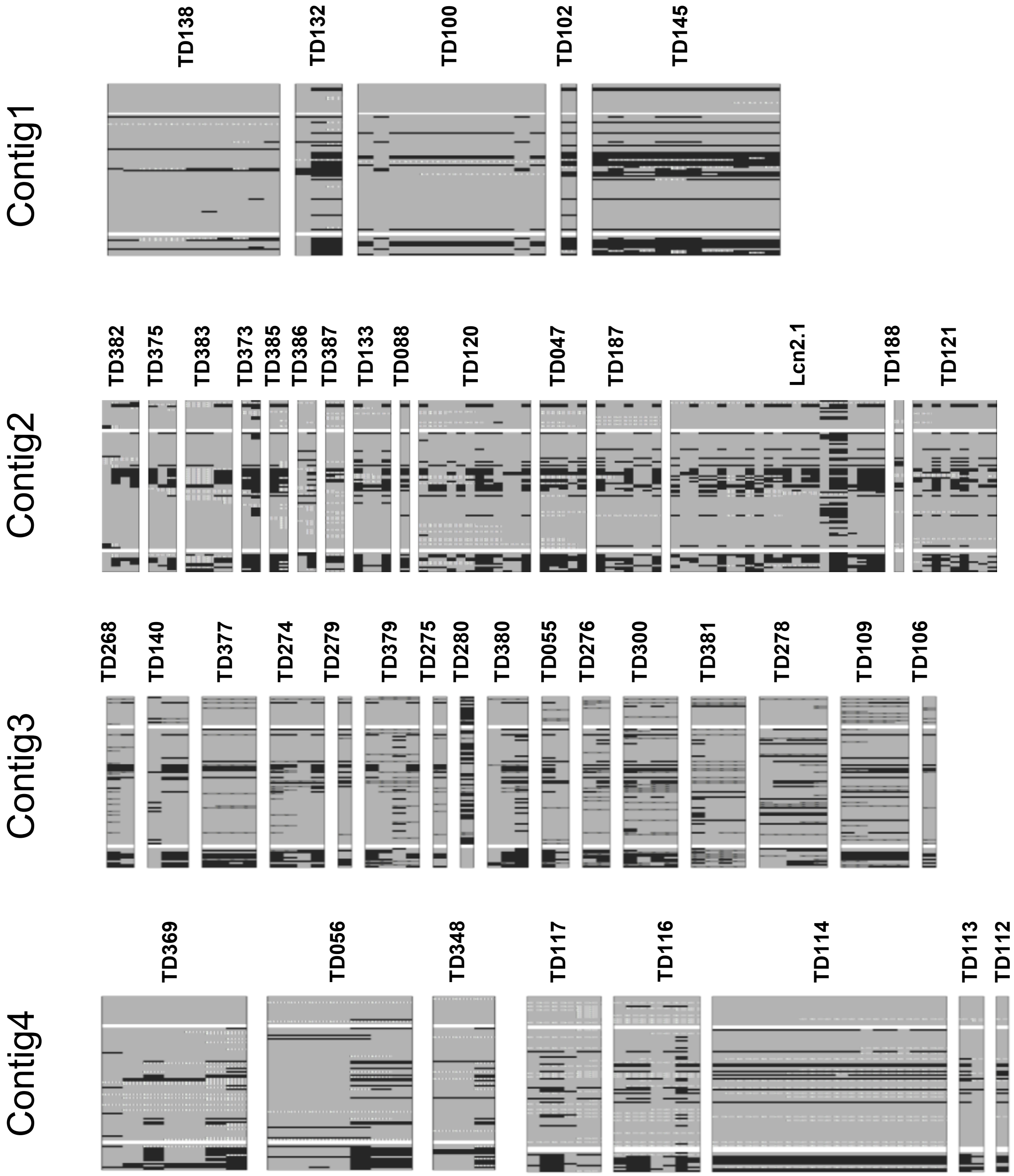

Figure S2 Graphical haplotypes of 90 accessions for markers located on physical contigs. Rows represent accessions and columns represent polymorphic sites. Fragment are separated by white rows. For each polymorphic site, most frequent allele is represented in light gray and the other allele is represented in black. Data failed are represented in white. The three species *S. lycopersicum, S. l. cerasiforme* and *S. pimpinellifolium* are separated by continuous white lines.

A

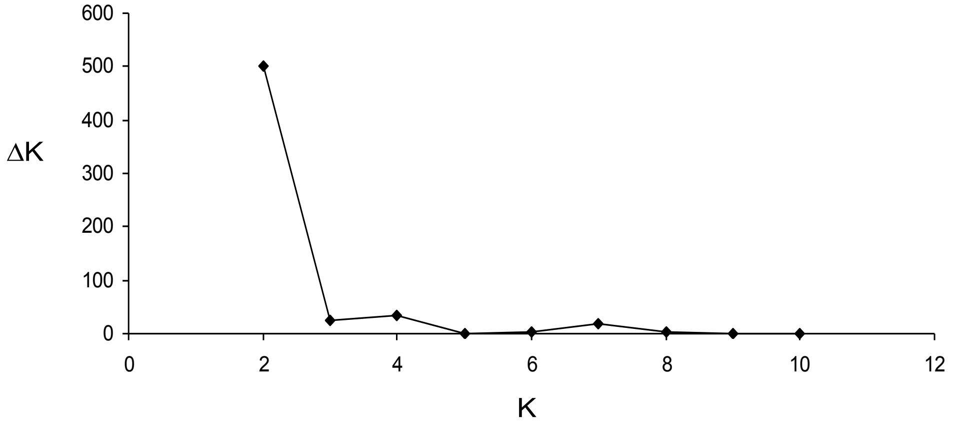

B

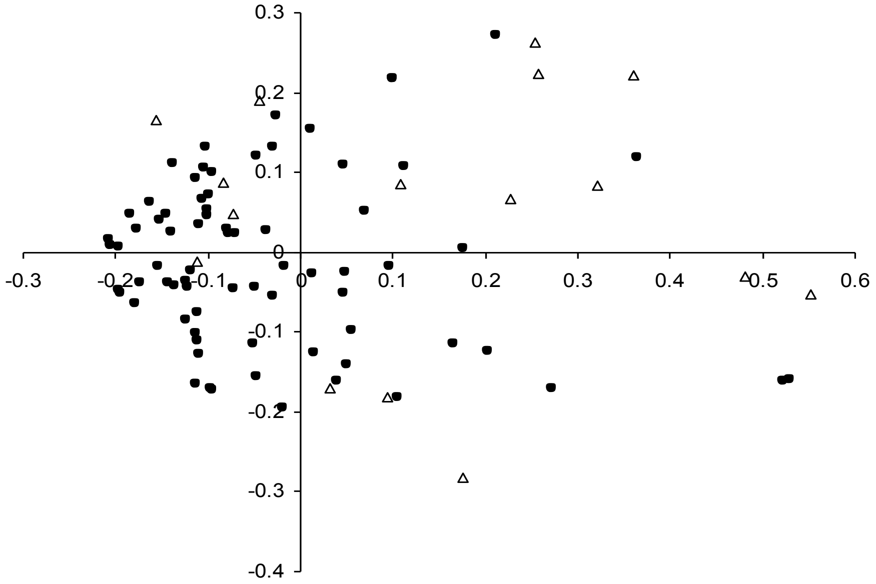

Figure S3 Genetic structure determination of the 90 accessions of wild and cultivated tomato. (A) Determination of the optimal number of sub-population (K) for 90 accessions following the method of Evanno et al. (2005). The rate of change of the posterior probability of the data given the number of clusters is plotted against K, the number of clusters. ΔK was calculated as |L"(K)|/s[Pr(x|k)] (see Materials and Methods). The first peak (K = 2) corresponds to the optimum number of clusters. (B) Principal coordinate analysis of the 90 accessions based on 20 SSR markers. The two groups identified by Structure software are represented by black square and white triangle.

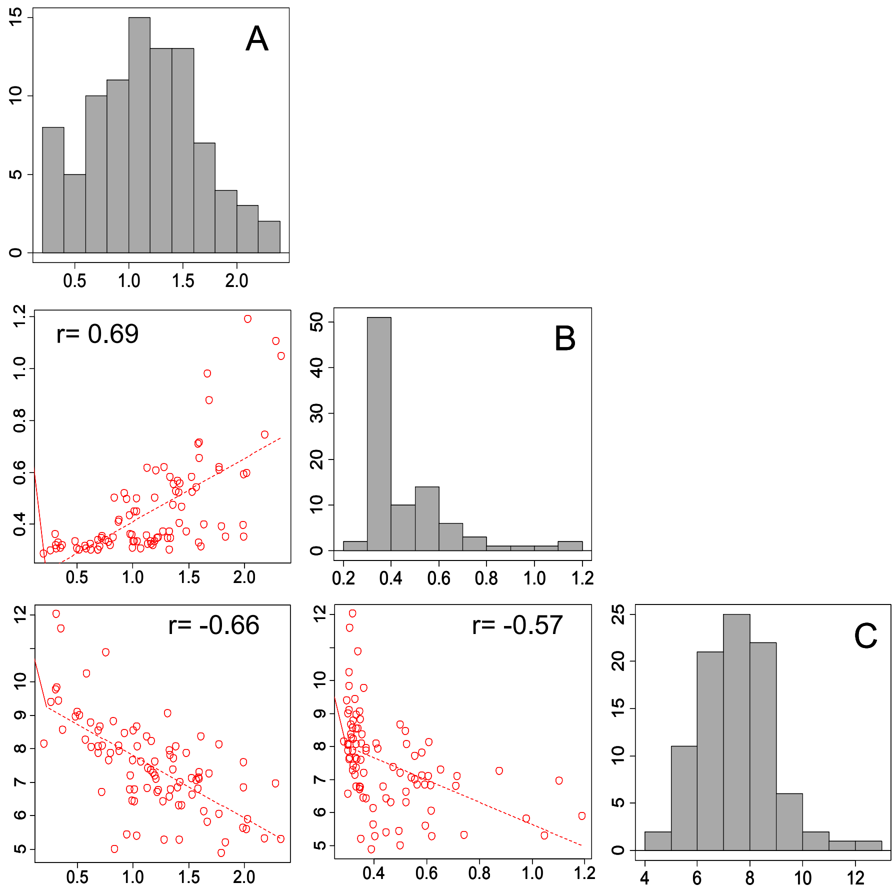

Figure S4 Distribution and correlation of fruit weight (FW), locule number (LCN) and soluble solid content (SSC) for the 90 accessions. Adjusted mean over two year of experiment for each accessions are used to show histogram distribution of traits for logarithm of Fruit Weight in gram (A), logarithm of Locule Number (B) and Soluble Solid Content in °brix (C). Scatter plot diagrams show correlation between traits and Spearman's rank correlation coefficient are indicated.

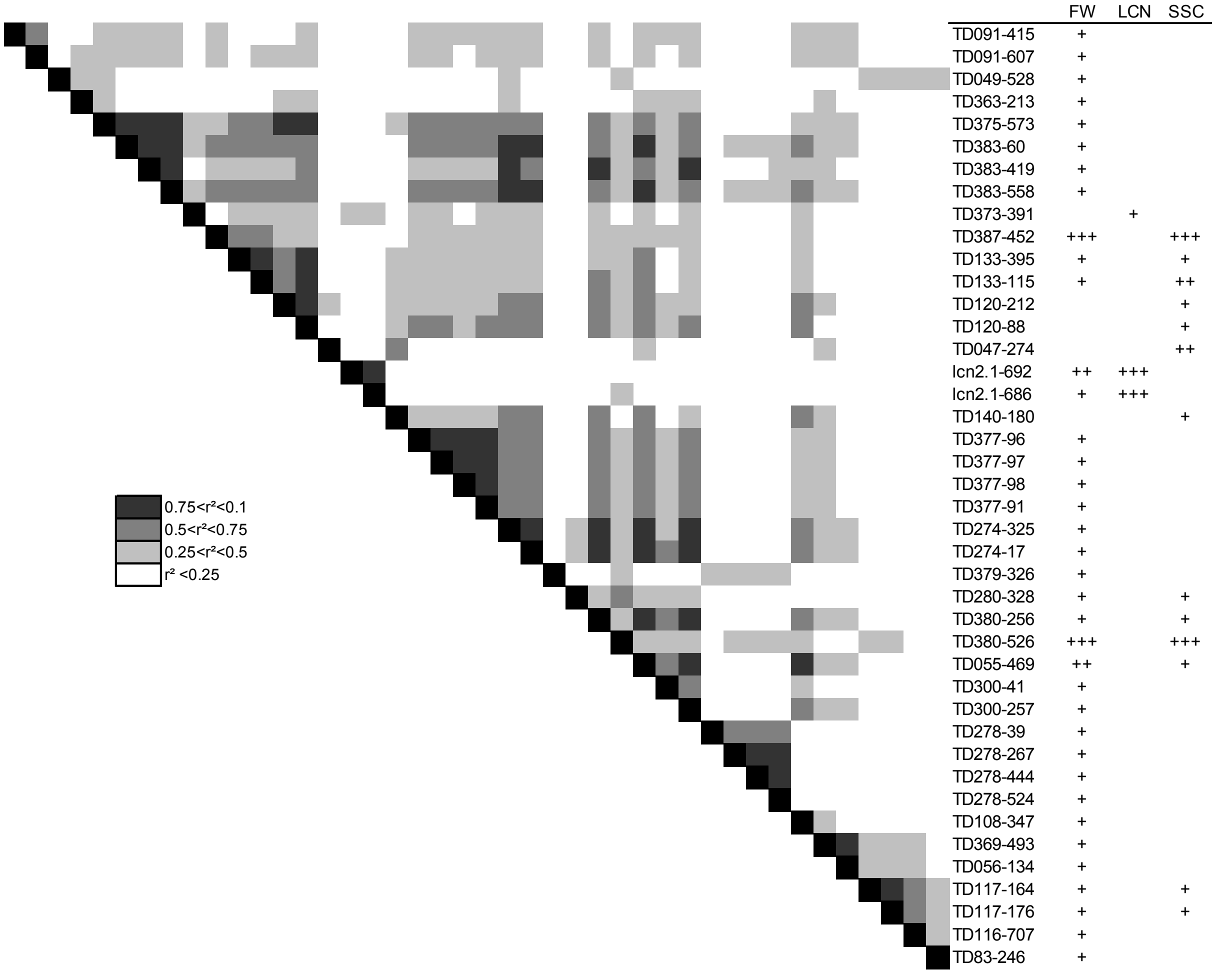

Figure S5 Matrix of linkage disequilibrium between markers significantly associated with fruit weight (FW), fruit locule number (LCN) and soluble solids content (SSC). Significant associations are indicated with the following symbol: +: <0.001; ++: <0.0001; +++: <0.00001.

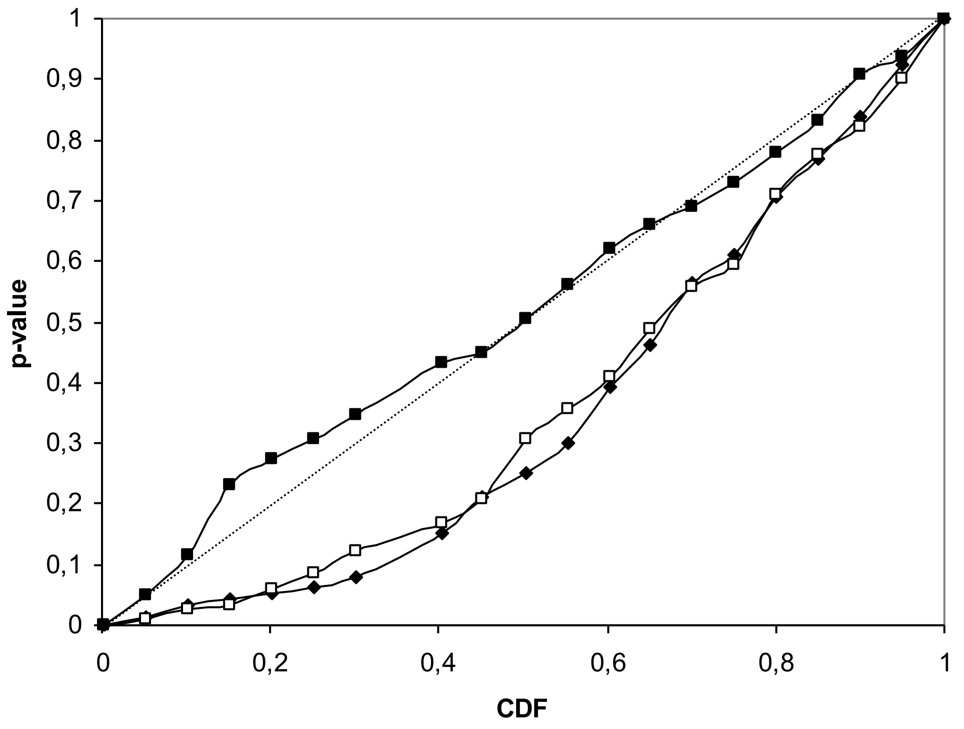

Figure S6 Cumulative density functions (CDF) using several alternative models of association for fruit weight on the subset of 63 cerasiforme tomato. Associations are tested for all polymorphic sites with MAF>5% on 63 individuals. Naive GLM (black diamond) and K+Q models, with structure based on SSR markers (white squares), on 4 PCA axis (white circles) and on all STS markers (black squares) were tested. The diagonal indicates uniform distribution of *p*-values under the expectation that random SNPs are unlinked to the polymorphisms controlling these traits (H0: no SNP effect).

Table S1 Accessions used in the association study

Accession Number	Accession Name	Species [a]	traits [c]			STRUCTURE results [c]	
			FW (g)	LCN	SSC (°brix)	pop1	pop2
CR001	Cervil	*S. l. cera*	5.80	2.18	10.88	0.393	0.607
CR002	Levovil	*S. l. esc*	109.13	3.91	6.83	0.188	0.812
CR003	Ferum	*S. l. esc*	109.66	2.24	7.59	0.092	0.908
CR004	M-82	*S. l. esc*	62.60	2.45	4.88	0.04	0.96
CR014	Clémentine	*S. l. cera*	5.40	2.25	8.08	0.012	0.988
CR020	San Marzano	*S. l. esc*	70.00	2.25	5.20	0.01	0.99
CR028	Plovdiv XXIVa	*S. l. cera*	41.58	2.05	8.35	0.012	0.988
CR031	Microtom	*S. l. esc*	6.91	3.17	5.00	0.017	0.983
CR032	Moneymaker	*S. l. esc*	99.54	2.49	5.63	0.008	0.992
CR056	Wva 700	*S. l. cera*	4.25	2.10	8.77	0.015	0.985
CR058	Wva 106	*S. l. cera*	9.69	2.72	6.78	0.99	0.01
CR062	LA 1478	*S. pimpi*	2.05	2.30	9.77	0.459	0.541
CR068	N° 108 Red Currant	*S. pimpi*	2.00	1.98	9.38	0.363	0.637
CR070	N° 2909 Lycopersicon sp.	*S. l. cera*	5.12	2.05	8.65	0.989	0.011
CR072	N° 2921 Lyc. Pimpinellifolium	*S. pimpi*	2.15	2.02	9.82	0.438	0.562
CR075	N° 4156 Blumen Strauss	*S. pimpi*	1.66	1.93	8.13	0.791	0.209
CR076	N° 135 Green Gage	*S. l. cera*	39.90	2.13	7.13	0.157	0.843
CR077	N°1565	*S. l. cera*	10.19	2.28	6.43	0.008	0.992
CR078	N° 2759 Enano	*S. l. cera*	34.84	3.33	6.62	0.135	0.865
CR079	N° 933	*S. l. cera*	33.64	3.82	7.12	0.009	0.991
CR093	N° 2257 Dikorastushii...	*S. l. cera*	23.55	3.58	7.70	0.058	0.942
CR094	N° 1011 Srednei Velichiny	*S. l. esc*	23.30	2.98	7.37	0.008	0.992
CR097	N° 347 Yablochnyi	*S. l. cera*	30.49	2.35	7.87	0.022	0.978
CR098	N° 795 Pescio	*S. l. cera*	22.11	3.82	7.80	0.015	0.985
CR101	N° 884 Alagabotskii	*S. l. cera*	24.49	3.35	8.07	0.02	0.98
CR102	N° 739	*S. l. cera*	54.13	7.52	7.25	0.412	0.588
CR106	LA 1025	*S. l. cera*	15.08	2.10	8.22	0.478	0.522
CR108	LA 1231	*S. l. cera*	4.99	2.18	8.53	0.346	0.654
CR110	LA 1307	*S. l. cera*	14.75	2.15	7.35	0.286	0.714
CR117	LA 1388	*S. l. cera*	16.89	4.05	7.10	0.009	0.991
CR118	LA 1420	*S. l. cera*	39.68	5.13	6.80	0.962	0.038

Accession Number	Accession Name	Species [a]	traits [c]			STRUCTURE results [c]	
			FW (g)	LCN	SSC (°brix)	pop1	pop2
CR122	LA 1456	*S. l. cera*	4.96	2.00	8.07	0.01	0.99
CR123	LA 1461	*S. l. cera*	3.93	2.02	10.23	0.332	0.668
CR124	LA 1464	*S. l. cera*	3.25	2.02	9.10	0.008	0.992
CR125	LA 1482	*S. l. cera*	9.66	2.30	7.20	0.023	0.977
CR129	LA 0147	*S. l. esc*	116.77	3.94	5.58	0.072	0.928
CR130	LA 0172	*S. l. cera*	37.68	3.48	7.05	0.398	0.602
CR133	LA 0409	*S. l. esc*	116.72	15.48	5.88	0.011	0.989
CR134	LA 0466	*S. l. esc*	208.89	12.71	6.95	0.039	0.961
CR136	LA 0473	*S. l. esc*	49.89	9.53	5.80	0.009	0.991
CR145	LA 1543	*S. l. cera*	11.27	2.15	8.07	0.053	0.947
CR149	LA 2095	*S. l. cera*	26.90	3.60	7.00	0.419	0.581
CR150	LA 2131	*S. l. cera*	40.36	4.51	7.28	0.025	0.975
CR152	LA 2307	*S. l. cera*	26.00	3.33	6.30	0.38	0.62
CR153	LA 2308	*S. l. cera*	27.70	2.92	6.30	0.01	0.99
CR155	LA 2402	*S. l. cera*	6.77	2.23	8.82	0.009	0.991
CR156	LA 2619	*S. l. cera*	13.77	4.13	6.82	0.93	0.07
CR158	LA 2675	*S. l. cera*	4.99	2.00	7.87	0.009	0.991
CR159	LA 2688	*S. l. cera*	4.34	2.00	8.03	0.467	0.533
CR163	LA 0400	*S. pimpi*	2.10	2.08	12.02	0.973	0.027
CR164	LA 0411	*S. pimpi*	3.14	2.15	8.95	0.884	0.116
CR169	LA 1371	*S. pimpi*	2.30	2.03	11.58	0.988	0.012
CR173	LA 1547	*S. pimpi*	3.42	2.00	8.98	0.992	0.008
CR186	LA 1689	*S. pimpi*	2.20	2.13	9.43	0.992	0.008
CR199	tomate Richter's	*S. l. cera*	3.82	2.07	8.27	0.988	0.012
CR202	CGN 18399	*S. l. cera*	6.45	2.08	7.87	0.985	0.015
CR203	LA 1589	*S. pimpi*	2.40	2.08	8.57	0.991	0.009
CR205	L. pimpinellifolium atypique, site 10 (F300045)	*S. l. cera*	10.42	2.15	6.78	0.994	0.006
CR234	Atom	*S. l. cera*	26.50	2.53	5.27	0.186	0.814
CR236	PI 365923	*S. l. cera*	15.32	2.08	7.27	0.057	0.943
CR238	PI 129088	*S. l. cera*	12.33	3.15	8.65	0.46	0.54
CR240	L 285	*S. l. cera*	15.96	2.16	7.60	0.158	0.842
CR244	Yellow Pear	*S. l. cera*	19.05	2.35	6.42	0.018	0.982

Accession Number	Accession Name	Species [a]	traits [c]			STRUCTURE results [c]	
			FW (g)	LCN	SSC (°brix)	pop1	pop2
CR249	Cherry Gold	*S. l. cera*	7.53	2.56	8.09	0.71	0.29
CR250	Cherry VFNT	*S. l. cera*	21.60	2.00	6.57	0.306	0.694
CR252	Droplet	*S. l. cera*	16.71	2.22	6.70	0.205	0.795
CR253	Monplaisir	*S. l. cera*	22.49	2.22	6.77	0.275	0.725
CR254	Farthest North	*S. l. cera*	8.91	3.13	5.43	0.375	0.625
CR256	Minibel	*S. l. cera*	19.45	4.17	5.27	0.029	0.971
CR258	Ohmiya Suncherry	*S. l. cera*	13.82	2.10	7.40	0.44	0.56
CR267	Tiny tim	*S. l. cera*	11.08	2.80	5.38	0.292	0.708
CR271	Celsior	*S. l. cera*	12.12	2.02	7.60	0.013	0.987
CR273	Orange Cocktail	*S. l. esc*	60.71	4.07	8.12	0.247	0.753
CR274	Marpha n°2	*S. l. cera*	8.54	3.30	8.47	0.009	0.991
CR275	Cerise Ildi	*S. l. cera*	7.65	2.60	7.92	0.008	0.992
CR279	Cerise Orange d'Uzès	*S. l. cera*	13.82	2.27	8.37	0.21	0.79
CR280	Cerise du sud ouest n° 2	*S. l. cera*	10.22	2.15	8.53	0.008	0.992
CR284	cerise rose	*S. l. cera*	10.45	2.80	6.42	0.104	0.896
CR287	Cisterno	*S. l. cera*	21.55	2.35	7.95	0.011	0.989
CR288	Criollo	*S. l. cera*	26.11	3.67	6.83	0.337	0.663
CR291	Pyriforme	*S. l. cera*	10.04	2.03	7.65	0.008	0.992
CR292	8 bis	*S. l. cera*	20.65	2.22	9.05	0.138	0.862
CR293	Costa Rica	*S. l. cera*	15.87	3.17	7.20	0.019	0.981
CR294	Phyra	*S. l. cera*	5.25	2.22	6.70	0.546	0.454
CR296	Poire jaune	*S. l. cera*	16.94	2.23	6.75	0.007	0.993
CR317	Heinz 1706	*S. l. esc*	43.70	2.50	6.12	0.008	0.992
CR321	Edkawy	*S. l. esc*	224.30	11.18	5.30	0.01	0.99
CR341	Cra 66	*S. l. esc*	40.45	5.18	7.10	0.186	0.814
CR354	Stupicke Polni Rane	*S. l. esc*	61.21	4.16	6.05	0.024	0.976
CR359	Muchamiel	*S. l. esc*	172.94	5.54	5.32	0.008	0.992

[a] Accessions are part of *S. l. cerasiforme* (*S. l. cera*), *S. l. esculentum* (*S. l. esc*) or *S. pimpinellifolium* (*S. pimpi*).
[b] Values for fruit weight (FW), locule number (LCN) and soluble solid content (SSC) are adjusted mean from two years of experiment.
[c] STRUCTURE software results are probability of membership in each subpopulation and are based on 20 SSR markers described in Ranc *et al.* 2008

Table S2 Description of the 81 DNA fragments sequenced located on chromosome 2

fragment name	F-primer sequence<br>R-primer sequence	Putative Function [a]	Unigene	BAC overgo [b]	Original marker name [c]	genetic position on chr. 2 [d]
TD018	CCGCCGCTCTTTCTTTGCT<br>TTCATGACTCCAGCTGGTC	pyrophosphate-fructose-6-phosphate 1-phosphotransferase beta	SGN-U583285	C02HBa0175O20	-	75.7cM (Expen2000)
TD047	GTTAACGTGGTGTAGGTGCA<br>AAGGTTGCAGGTACCTCTTGA	promoter of homeodomain transcription factor (WUSCHEL), putative	SGN-U589573	C02SLm0057H03; Le_HBa0139K19	-	88.0cM (Expen2000)
TD049	ACGGTCCTAATTGCTAACGCA<br>CTTGGGCCATAATGTAATTGTCT	lactoylglutathione lyase / glyoxalase I, putative	SGN-U578074	C02HBa0167J21	-	72cM (Expen2000)
TD055	AGTTTGAAGCTTCGGTTCTCC<br>TACCTACAGTAGGTGGGTGT	ovate protein	SGN-U582169	C02SLm0128E12	ovate	89.5cM (Expen2000)
TD056	GATTGCGCATTGAGATGCT<br>CGGGGGCAGATACATAGTGA	5' region of fw2.2 gene (Frag 4 : Nesbitt & Tanksley 2002)	no	C02HBa0208N01; BAC clone FW2.2	fw2.2	116cM (Expen2000)
TD083	TAGTGCCGGATCCGCTATG<br>TATTAGTCCCAGCCTTTGCA	sucrose-responsive element binding factor, myb family transcription factor, putative	SGN-U569474	no	cLEX-2-I9	133cM (Expen2000)
TD085	CCAACTTCAACTTGTTTGGGA<br>AGGGACTTCCATAATCATATC	homology with ubiquitin interaction motif-containing protein / LIM domain-containing protein	SGN-U584698	C02HBa0111M10	cLPT-1-A21	114cM (Expen2000)
TD086	GACCAGAGCGTGCTTCTTGA<br>TACCATTCTGGGAGCGGTAT	mitochondrial processing peptidase beta subunit, putative	SGN-U580309	C02SLm0025E22	cLET-5-L17	103cM (Expen2000)
TD088	TAGATGGAGTGGGTATTGTTGA<br>CAAAGTCGGTCTTAACACGACA	-	no	C02SLm0057H03	cTOF-13-F10	86cM (Expen2000)
TD090	ATGGATATGGTAATTGGAGGA<br>CATGAGTTGAGCTTCATGACT	transcription factor, myb-like protein, putative	SGN-U574375	C02HBa0059M17; C02HBa0031A21	T0266	67cM (Expen2000)

fragment name	F-primer sequence<br>R-primer sequence	Putative Function [a]	Unigene	BAC overgo [b]	Original marker name [c]	genetic position on chr. 2 [d]
TD091	TCTTGGCATTCGCACAGGAA<br>CCTGGGAGTTATGGGATCTT	expressed protein similar to UV-B and ozone similarly regulated protein	SGN-U580690	no	TG14	54cM (Expen2000)
TD092	GTAGAGTGTGGGAATATGGAG<br>CCACTAAGCAAAGCTAACCA	-	no	C02HBa0072A04	TG451	46cM (Expen2000)
TD093	TTGGGAGAGGACGAAGGA<br>CTCATCATAAGGCTCTTGCT	expressed protein similar to Glutathione S-transferase	SGN-U583311	C02HBa0320M09	T1698	36cM (Expen2000)
TD094	TTAACGTACTCGTTGCGTGC<br>ATTGGAAATCCCAACAGCCA	CHP-rich zinc finger protein, putative	SGN-U583902	C02HBa0209K17; C02HBa0025A22	cLED-19-B18	27cM (Expen2000)
TD095	GAAGAACATGAGAAGCAGCC<br>AGTTCCTACCCACAAGTATCA	expressed protein	SGN-U583803	C02HBa0163K16	CT140	16cM (Expen2000)
TD096	CAAACTACCCCCAGGTCCA<br>GTATCAATCTCGTCTCGGAGT	cinnamic acid 4-hydroxylase (C4H), putative	SGN-U581122	C06SLe0014B21.1	T0123	5cM (Expen2000)
TD098	CCAAGGCAGAGATAAACGTG<br>CCATGAGGTTCCTACACATC	Rho-GTPase-activating protein-related, putative	SGN-U580990	C02HBa0060J03	T0759	82cM (Expen2000)
TD100	ATCTCTCTGAGGGTTCAAGACAGG<br>TATATCAGCTCCATACTTCTTTGC	expressed protein	SGN-U567423	C02HBa0159F19; C02HBa0030D08	C2-At5g66090	83.2cM (Expen2000)
TD102	TCTGAAGAAGCTGAAGCAAGTAGAGC<br>TGCCAACTGACGAGCATAAGCTGC	expressed protein	SGN-U565307	C02HBa0159F19; C02HBa0030D08	C2_At3g01160	83.4cM (Expen2000)
TD106	CTGGCATGGGATGTAGTGC<br>GATGCAGAAATGTTCGAAGGC	CER6; very-long-chain fatty acid condensing enzyme (CUT1 homology)	SGN-U566767	C02HBa0164H08	T1535	89cM (Expen2000)
TD108	GTGAATTGTCCGGTTCTCGT<br>AATGCTCATCCCTTGTTTGC	expressed protein	SGN-U575206	C02HBa0134G09; C02HBa0011O23	U146494	90cM (Expen2000)

fragment name	F-primer sequence<br>R-primer sequence	Putative Function [a]	Unigene	BAC overgo [b]	Original marker name [c]	genetic position on chr. 2 [d]
TD109	GTCTACCCTGCTGCACAAC<br>CTGTTGAACTGTGGTCTCCA	RAD23; ubiquitin family protein	SGN-U569990	C02HBa0164H08	CT232	90.1cM (Expen2000)
TD110	CACCTCCCAGATCACAATG<br>CATGTGTGGTACATGCTCTG	chlorophyll a/b binding protein CP24 10B, putative; homology to peroxidase 42	SGN-U577555	C02SLm0008E03	T1395	72.0cM (Expen2000)
TD111	CAGTGGTGCTGAAGATGTTG<br>GTAAGCCCTTTAGAGCTCTC	asr; Adenylyl sulfate reductase (homology to APR1)	SGN-U580331	C02HBa0167J21	-	NA
TD112	GCGAGGATAACGGTGAGAAG<br>TGCCTTTGGAGACTCCTTGT	protein kinase, putative	SGN-U563886	CO2Hba0073P13	U153274	120cM (Expen2000)
TD113	TTCATTGATTTCTCCGCTGC<br>TGAACCACCAAGACGGGA	frk3, fructokinase 3	SGN-U570882	CO2Hba0073P13	-	120cM (Expen2000)
TD114	CTGTAAAGAGGGTGCCTAC<br>CGGTACTTGGTTCAAACCTG	expressed protein	SGN-U573852	CO2Hba0073P13	-	120cM (Expen2000)
TD116	CCTGGAATTCGTGCCTTGC<br>GATACCACTAAGTACAGCCTC	oligopeptidase A, putative	SGN-U565226	CO2Hba0073P13	-	120cM (Expen2000)
TD117	GACTCTTCTTTGGTGCTGC<br>CCAACTGCTTCGCTTCTCT	expressed protein	SGN-U598856	CO2Hba0073P13	-	120cM (Expen2000)
TD125	TCATGAGCAACCTGCATATG<br>TAGGCTCAATGTCATTGATCAC	cyclin family protein, similar to cyclin D3.1 protein	SGN-U583476	no	-	2-K(CL) (ILs pennellii)
TD120	ACCCAACACTCTAGCCCAACT<br>ATGGCCGTAATTTTCGTAATCATCA	-	no	Le_HBa0139K19	-	physical map Contig 2
TD121	GTAGTACGTATAGAATGGGTTGT<br>TAATGATGCGGCACTTGCTCA	-	no	C02SLm0057H03; Le_HBa0139K19	-	physical map Contig 2

fragment name	F-primer sequence<br>R-primer sequence	Putative Function [a]	Unigene	BAC overgo [b]	Original marker name [c]	genetic position on chr. 2 [d]
TD129	TGAATTTTGGGAAGTCTGGTT<br>TGCAACTCTACCTCTTTTTCAGC	nuclear matrix constituent protein, putative	SGN-U584279	no	TG151	126cM (Expen2000)
TD130	GGTTTTGACTTGACATGAAG<br>CTTATTCTCCAAACAATTGC	-	no	C02SLm0065M14; C02HBa0016A12	TG518	95cM (Expen2000)
TD132	CGATCGTGCATACTCTCGTG<br>GCTTCTACTGATGATCCTAC	-	no	C02SLe0127J16; C02HBa0159F19; C02HBa0030D08	TG278	81,2cM (Expen2000)
TD133	TTGGGCGACCACGCTGAATC<br>TTACCCACATCAGGACCTTGCC	peptide methionine sulfoxide reductase, putative	SGN-U576565	C02HBa0130B04	C2_At2g18030	83,1cM (Expen2000)
TD137	ACCTAGAGAGGACCTTCCAGAGCCC<br>AGGAATTCAGTGCCTTCAATGCAG	hydantoin utilization protein-related, putative	SGN-U562909	C02SLm0020J02	C2_At4g20070	29cM (Expen2000)
TD138	TTTTCACCAGTAGGAACATTCAAGGTAC<br>CAGGATAATAAACCATCATGCCACAA	Z15141; chitinase (endo-), acidic, 26 kD	SGN-U581507	C02HBa0238L13; C02HBa0013N18; C02SLe0031D11	cLET1A5	79cM (Expen2000)
TD139	ATCCTCTGCCCTTTCTTTCC<br>CAATTGCAGGGGATATGCTT	transport protein, ATPase, gamma chain, chloroplast, putative	SGN-U581255	C02HBa0167J21	T0562	71cM (Expen2000)
TD140	TTCAGGAATGGCATTTGCAAGTGTG<br>ACCATTGAATACAGCATCTGGTCGAAC	aldose 1-epimerase family protein, similar to apospory-associated protein C	SGN-U585203	C02HBa0213A01; C02HBa0030A21	C2_At5g66530	87.5cM (Expen2000)
TD145	GCCAATTTCTAGCGAACATG<br>TCTGTTTCTTCCAACACTAG	histidine kinase family, putative	SGN-U603568	C02SLm0020N19; C02HBa0009K06	TG191	83.30cM (Expen2000)
TD150	GACTCCAGAAGCGATTCTGT<br>CTTCGGCAACTCCTCTTAGC	Cnr; squamosa promoter-binding protein-like 3	SGN-U576708	no	-	60;0cM (Expen2000)
TD187	GCGGAGCTTGAAATCAGTAAC<br>AAGGCACGCATGAGATGATAA	-	no	C02SLm0057H03; Le_HBa0139K19	-	physical map Contig 2

fragment name	F-primer sequence<br>R-primer sequence	Putative Function [a]	Unigene	BAC overgo [b]	Original marker name [c]	genetic position on chr. 2 [d]
TD188	AAACTCACTTACCACTTTCC<br>CTCCTAGTAAGTCTCGATTC	-	no	C02SLm0057H03;<br>Le_HBa0139K19	-	physical map Contig 2
TD265	TGCTCCAAAGTGTGCTCATC<br>AGCGGTACCTTTCTCCTGGT	er60; ethylene-responsive catalase	SGN-U578479	no	T0200	81cM (Expen2000)
TD268	ATTGGCAAATGAGCTTGCTT<br>TTGCAAAAAGAACGGTTTCC	expressed protein	SGN-U585443	C02HBa0213A01;<br>C02HBa0030A21	HBa0030A21	88.5cM (Expen2000)
TD270	AATGCACATGCGTCACAAAT<br>TTGCACCCTCTCATCAACAG	chaperonin, putative (response to oxidative stress)	SGN-U576925	no	cLEM-23-E21	11.0cM (Expen2000)
TD272	CTACCTGGATCGCAATGGTT<br>TTAGGTGCAACAGCATCTCG	peptidyl-prolyl cis-trans isomerase, putative (protein folding)	SGN-U573199	C02HBa0236E02;<br>C02HBa0016A12	T1475	90.5cM (Expen2000)
TD274	GCTAAATCGAATGCCTGAGC<br>GGAAACCGCCAAAACTATCA	integral membrane protein / sugar transporter family protein, putative	SGN-U575594	C02HBa0074A14;<br>C02HBa0030A21	-	physical map Contig 3
TD275	GAGGGTGAGCGATTTATGGA<br>GTCCAGGGATCACAGCATCT	succinate dehydrogenase flavoprotein, putative (nergy pathways; carbohydrate metabolism; citric acid cycle; highly expressed in fruit)	SGN-U580353	C02SLm0128E12;<br>C02HBa0215M12;<br>C02HBa0023N04	-	physical map Contig 3
TD276	AGTTGACGTGTGGCTTACCC<br>CAGGCTTTTTCTCCTTGACG	SIN-like family protein, putative (transcription)	SGN-U572730	C02SLm0128E12;<br>C02HBa0106H06	-	physical map Contig 3
TD278	GCCGAACATGAGAAGGAGAG<br>CAGCTAACCATGACGAGCAA	flavodoxin family protein, putative	SGN-U576263	C02HBa0164H08	-	physical map Contig 3
TD279	AGCTCCTTACAGAGGCAGCA<br>ACCCAAGGGACAGCCTAGTT	contig_ovate16	no	C02HBa0215M12;<br>C02HBa0074A14	-	physical map Contig 3
TD280	ACATCCAAGCATGGGCTAAT<br>TGGGCACACAATGCTTAGAA	contig_ovate22	no	C02SLm0128E12;<br>C02HBa0215M12;<br>C02HBa0023N04	-	physical map Contig 3

fragment name	F-primer sequence<br>R-primer sequence	Putative Function [a]	Unigene	BAC overgo [b]	Original marker name [c]	genetic position on chr. 2 [d]
TD300	TGGATAGCACGTGAAATGGT<br>AATGGAAATCCAGGATCAGC	contig_ovate35	no	C02SLm0097L01;<br>C02HBa0164H08	-	physical map Contig 3
TD304	GTTCATTCTGGGGATGGGT<br>TGCAGCTATCCTTGCTTTG	expressed protein	SGN-U586574	C02HBa0111M10	C2_At1g19690	113.0cM (Expen2000)
TD305	TGGTGAATGGAGAAATGCAG<br>TGATGCCCACTTACACAAGC	expressed protein	SGN-U569081	C02HBa0136C06	C2_At1g20770	99.5cM (Expen2000)
TD316	GATGCTGCCTTATTTGCTC<br>CCATCTCAGGGTGTTTTGT	cellular repressor of E1A-stimulated genes (CREG) family, putative	SGN-U578677	C02SLe0128J14;<br>C02HBa0189G15	C2_At2g04690	97.30cM (Expen2000)
TD328	CCGTTGGTTGGATATTGCTT<br>AAAAGGCACCCCAAAAGAGT	regulator of chromosome condensation (RCC1) family protein, putative	SGN-U565338	C02SLe0092M23;<br>C02HBa0046M08	C2_At3g02300	111.80cM (Expen2000)
TD339	CTCATCTTCAACTTCCCTTCC<br>CATCAACCACTGAGCCAAC	inositol monophosphatase family protein, putative	SGN-U572028	C02HBa0161P02;<br>C02HBa0031A21	C2_At4g05090	69.80cM (Expen2000)
TD343	TCCGCCGTATCTAACCTATC<br>CTGTCCAGTAGTAGCATCCC	transducin / WD-40 repeat protein family, putative	SGN-U565169	C02SLm0114O11;<br>C02HBa0329G05	C2_At4g21520	69.70cM (Expen2000)
TD345	GAAGTGGAAGACCCACAAA<br>CCACTAGAGCCTCCATGTATC	NADPH quinone oxidoreductase-like protein, putative	SGN-U579777	C02HBa0090O01	C2_At4g21580	68.50cM (Expen2000)
TD348	ATTCGCCAGAAATGGATCAG<br>TGGTTGCAACACAATCATCA	expressed protein	SGN-U595227	C02HBa0208N01;<br>C02HBa0012A12	C2_At4g33985	118.50cM (Expen2000)
TD350	GAAAGGAAGCAACCCAATC<br>GCTTAATCCTCGACCAGACA	expressed protein	SGN-U563682	C02SLe0026H18	C2_At4g35560	78.50cM (Expen2000)
TD356	TATGTGGGCAACAAGTCAGC<br>CAAAAAGGAGACCGAACCAA	pyridoxal kinase, putative	SGN-U580571	C02SLe0054B08	C2_At5g37850	119.50cM (Expen2000)

fragment name	F-primer sequence<br>R-primer sequence	Putative Function [a]	Unigene	BAC overgo [b]	Original marker name [c]	genetic position on chr. 2 [d]
TD363	ACCCGTTCAGTCTCACATTTC<br>CCAATGCTATCCACCTTATCC	ribosomal protein L15 family protein, putative	SGN-U583446	C02HBa0144P17	C2_At5g64670	76.00cM (Expen2000)
TD369	TCCTGAGGACATTGGACACA<br>TGGCAGAAACCTCCATTCTT	weak homology with nodulation protein-related	SGN-U570126	BAC clone FW2.2	-	physical map Contig 4
TD373	CAAGCAGCCAAGATCTGTCA<br>TCCCATCTTCAAACCTGGTC	expressed protein	SGN-U581635	C02SLm0132H19; C02HBa0044J01	-	physical map Contig 2
TD374	AAGAGGAGAAGGCCCAGAAG<br>CTTTCTGTGTCCGAGGAAGC	expressed protein	SGN-U563261	C02HBa0044J01	-	physical map Contig 2
TD375	CGCGGTACACCGTCTTTTAT<br>TTCACATTTTCTGGCCTTCC	plastidic fructose-bisphosphate aldolase (photosynthesis; Calvin cycle; carbon fixation)	SGN-U580022	C02SLm0132H19	-	physical map Contig 3
TD376	AAGGGCCTTCAGATGAGGTT<br>CCGATTGCCTCTCTTAGTGC	vesicle tethering family protein, putative	SGN-U582526	C02HBa0074A14; C02HBa0215M12	-	physical map Contig 3
TD377	CAAGACGATGCGAAAGATGA<br>CAGCATTCATGGAATCATGC	-	no	C02HBa0074A14; C02HBa0030A21	-	physical map Contig 3
TD379	TTAAAAGATGGGGCATGAGG<br>ACGTCAAACTGGACCAGACC	-	no	C02SLm0128E12; C02HBa0215M12; C02HBa0023N04	-	physical map Contig 3
TD380	GCCTTGGAAACTCACGAAAG<br>GCGACAATATTTCGGGCTTA	chromatin remodeling complex subunit	no	C02SLm0128E12; C02HBa0215M12; C02HBa0023N04	-	physical map Contig 3
TD381	TTTGTTCCCCTGCGTAAGAG<br>GGGTATTTTAGGCCCTCGTC	-	no	C02HBa0164H08	-	physical map Contig 3
TD382	GCACGCCACGACAGTTACTA<br>ACGTTTTCTGCGCGAGTTAT	homology with retrotransposon Tork11	SGN-U594026	C02SLm0132H19	-	physical map Contig 2

fragment name	F-primer sequence<br>R-primer sequence	Putative Function [a]	Unigene	BAC overgo [b]	Original marker name [c]	genetic position on chr. 2 [d]
TD383	CTCCGTCCCTAGTTGTCCAC<br>CAGGCCATAATCCAAATGGT	acs8; 1-aminocyclopropane-1-carboxylate synthase	SGN-U565888	C02SLm0132H19	-	physical map Contig 2
TD384	CTGCAAGGGCTAGTTCAAGG<br>CGGGAGTGAGGTGTTTGAAT	putative receptor-like protein kinase gene	SGN-U603238	C02SLm0132H19	-	physical map Contig 2
TD385	AACAAAAGCACCACCAAAGG<br>AAAGGAGAGGCTCCGAGTTC	-	no	C02HBa0044J01	-	physical map Contig 2
TD386	TTAACAAGGGCGTGCACATA<br>CCCGTGCAATACCTTGATCT	-	no	C02HBa0044J01	-	physical map Contig 2
TD387	GAAAATGCAGGAGGAAACCA<br>ATGTGAATCCCGATAGCAACA	-	no	C02HBa0130B04; C02HBa0044J01	-	physical map Contig 2

[a] Putative functions of genes are given according to annotation of unigene (http://solgenomics.net/) or manual annotation.
[b] BAC overgo indicates sequence identity with BAC sequences available on genbank.
[c] Name of marker located on the reference map are indicated when available.
[d] Genetic distances are available from the Expen2000 reference map (http://solgenomics.net).

Table S3   Genotype – All SNP

Table S3 is available for download at http://www.g3journal.org/lookup/suppl/doi:10.1534/g3.112.002667/-/DC1 as an excel file.

Table S4 Polymorphism information

polymorphism name [a]	Allele in reference genotype	Second allele	Upstream sequence	Downstream sequence	Frequence of reference allele		
					S. l. cera (N=63)	*S. l. esc* (N=17)	*S. l. pimpi* (N=10)
lcn2.1-1023	C	T	TACGTATAAT	TAGACAAATA	0.86	0.88	0.70
lcn2.1-1073	A	T	AGTGTGATGG	GATAACGGAT	0.77	0.88	0.40
lcn2.1-1161	G	A	TGATGAAAAT	ACGGATGGAG	0.88	1.00	0.64
lcn2.1-1185	T	-	TGAGCATGAT	GAACGTTATT	0.74	0.88	0.10
lcn2.1-1362	A	G	CAGCCTCATA	TTAAATTACA	0.83	1.00	0.73
lcn2.1-1450	A	-	CAAAATAAAA	TTAGTTTTTC	0.91	1.00	0.90
lcn2.1-1480	A	G	AATTCAAATT	TGTTTAATGT	0.67	0.88	0.09
lcn2.1-1505	A	T	AAATATTTTT	AAAATTTTTT	0.83	1.00	0.82
lcn2.1-1536	G	A	CATATCACGA	AAATATCAGC	0.83	1.00	0.82
lcn2.1-1555	G	A	GCTTAAAATA	TTAATTTCTC	0.78	0.88	0.64
lcn2.1-1565	C	A	GTTAATTTCT	TCAATTTCAA	0.83	1.00	0.73
lcn2.1-1579	A	G	ATTTCAATTT	TTTGTCTTTA	0.83	1.00	0.64
lcn2.1-324	C	T	GTACAAATTA	GTTAACCAGA	0.69	0.88	0.27
lcn2.1-434	T	-	GTTTTTTTTT	GTTTTAAAAA	0.89	1.00	0.64
lcn2.1-53	A	G	TTAAATTAAT	ATTATTTTAA	0.69	0.88	0.09
lcn2.1-60	T	C	AATAATTATT	TAATTCAAAT	0.69	0.88	0.09
lcn2.1-686	T	C	TGGCATGATG	TTACTAATTG	0.60	0.44	1.00
lcn2.1-692	A	G	GATGTTTACT	ATTGGACAAT	0.62	0.44	1.00
lcn2.1-744	-	T	ATTTTTTTTT	GGACATATTT	0.19	0.31	0.00
lcn2.1-904	G	A	GGTTTGAAAT	TTGATGTGTT	0.74	0.88	0.40
lcn2.1-939	G	A	ATGTTTTTCA	AATTTTTTTT	0.78	0.88	0.40
lcn2.1-942	T	A	TTTTTCAGAA	TTTTTTTCGT	0.90	1.00	0.70
lcn2.1-964	G	A	TTCCTTGCTT	TTTTATGTGT	0.76	0.88	0.40
TD018-103	C	T	GAAGCCCTTT	AAAGTCGTTG	0.67	0.92	0.14
TD018-611	T	C	CAAACGCCAA	TAAGGAGGAT	0.78	1.00	0.14
TD047-220	C	T	TTTTTTAATA	GAGGAAATTT	0.73	0.80	0.09
TD047-274	ATTAAATTA ATGAAAGAT AA	----------------- ---	TTTTAAAATT	ATTAAATTAA	0.92	1.00	0.55
TD047-435	A	G	TCATGTAAAT	TTTTAAATAA	0.78	0.85	0.18
TD047-505	C	T	TATAACGATA	TTATAAAGTT	0.80	0.85	0.27
TD047-571	G	A	CATAACGAAC	ATTATTCAAG	0.74	0.85	0.09
TD047-573	---	GAT	AACGAACGAT	TATTCAAGA	0.74	0.83	0.09
TD049-339	C	A	GAGCTCATAT	CCATTGATCA	0.78	1.00	0.45
TD049-348	T	C	TCCCATTGAT	ACATACATGC	0.81	1.00	0.64
TD049-445	T	A	GAAATGAAGT	ATCTTGGTGT	0.81	1.00	0.64

polymorphism name [a]	Allele in reference genotype	Second allele	Upstream sequence	Downstream sequence	Frequence of reference allele		
					S. l. cera (N=63)	*S. l. esc* (N=17)	*S. l. pimpi* (N=10)
TD049-457	A	G	TCTTGGTGTT	AGTCCAGGAA	0.78	1.00	0.36
TD049-528	T	C	TTGACAACCT	CGCGCTTTTG	0.51	1.00	0.09
TD049-96	TCAAATTC	--------	GTTAGAGATG	ACATTGTAAC	0.78	1.00	0.45
TD055-418	A	G	GTACAGCGGG	TATTAAGCGG	0.93	1.00	0.80
TD055-469	T	A	GGAAACTGAA	CTTTAGTTTC	0.80	0.93	0.10
TD056-134	C	T	CTGACCCTCA	TCTCTTTTCT	0.68	0.93	0.09
TD056-155	A	T	TGGCATTATT	TCCAGAAAAG	0.74	0.93	0.27
TD056-252	AC	--	AAAAAACAAT	ACTCATTTAC	0.82	0.93	0.27
TD056-28	T	C	TTCATTTGAT	TGAAAACGAA	0.94	1.00	0.82
TD056-64	-	A	AGAATATTGT	ACGATTATAA	0.94	1.00	0.82
TD056-78	-	T	TTATAATTAG	TAAGCTTAAT	0.94	1.00	0.82
TD056-80	A	T	TATAATTAGT	AGCTTAATTT	0.94	1.00	0.82
TD083-222	C	A	GCCGATTCGA	TCAGTCGAAA	0.82	1.00	0.64
TD083-246	G	T	CCTTTTCAGT	GCTGCTTCCA	0.48	0.81	0.00
TD083-277	---	CCA	TCCTCCACCG	CCACCACCAC	0.92	1.00	0.64
TD083-366	G	C	CCTGATTTTG	GCAAAACTCA	0.97	1.00	0.64
TD083-404	G	T	CAAGGTATTT	TGCCGTTTAG	0.81	0.92	0.09
TD083-666	G	A	TGAATGGGAG	AAGAAACCGC	0.85	1.00	0.27
TD083-685	C	T	GCCTTTGTTT	ATTCTCTCTT	0.81	0.92	0.09
TD086_T7-434	A	G	ATAATCCTCA	TAAAATCTTA	0.88	1.00	0.73
TD086_T7-486	C	T	ACCTATGTTT	ATTGGATTCA	0.88	1.00	0.73
TD086_T7-523	A	G	TAAATAATAC	ATTAGATTTA	0.96	1.00	0.55
TD086_T7-671	G	A	TTAGCATCCA	GGAACTCAAC	0.95	1.00	0.45
TD086_T7-673	G	A	AGCATCCAGG	AACTCAACAA	0.95	1.00	0.45
TD086-312	A	T	AACTCGCCAC	CCCAACTTTA	1.00	1.00	0.64
TD088-204	G	T	AAAGGAAGAC	CTCCTTATTG	0.78	0.88	0.18
TD090-14	A	C	ATTTTCTTGT	TTAATTTATA	0.81	1.00	0.56
TD090-266	-	T	CTCATTTTTT	CTCTATAGGT	0.75	1.00	0.33
TD090-270	C	T	ATTTTTTCTC	ATAGGTTTGG	0.75	1.00	0.22
TD090-306	T	C	TATTTTGTCA	GTTCCTTAGA	0.92	0.92	0.89
TD090-357	C	T	TTTTTTGATT	AGGTGGTCTG	0.75	1.00	0.22
TD090-36	-	A	TTTTTTTTAA	GTTGTATTAT	0.75	1.00	0.33
TD090-625	A	T	TACTTCTCTT	TATCATCCCA	0.75	1.00	0.33
TD091-415	A	-	GGACAAAGGT	AAAAAAAAAA	0.86	1.00	0.30
TD091-607	T	C	TTTTGAGTTT	TAATCATTAC	0.80	1.00	0.10
TD092-450	C	G	ACATTGTATG	TAATTGCATG	0.80	1.00	0.36
TD092-484	A	G	CTTCTAAGGT	TTCAGCGAGA	0.91	1.00	1.00

polymorphism name [a]	Allele in reference genotype	Second allele	Upstream sequence	Downstream sequence	Frequence of reference allele		
					S. l. cera (N=63)	*S. l. esc* (N=17)	*S. l. pimpi* (N=10)
TD092-523	G	A	TTGTATGGAC	TCTCTTTTGA	0.80	1.00	0.27
TD093-148	C	T	AATAGAATCT	TAATAACATT	0.88	0.94	0.09
TD094-128	C	G	TCCCGAACAT	AATTAACTCT	0.80	0.94	0.55
TD094-131	T	C	CGAACATCAA	TAACTCTCAA	0.80	0.94	0.55
TD094-306	T	C	GCGTTATCTG	ATTTTACTCG	0.94	0.94	1.00
TD094-433	G	A	CGCATTTCGG	TGTGTTGCTG	0.94	1.00	0.90
TD094-447	T	C	GTTGCTGATA	TGTATATGAG	0.86	0.93	0.50
TD094-456	A	G	ATTGTATATG	GAAAGGCGGA	0.95	1.00	0.90
TD094-623	ATA	---	AGGTCGATGG	ATAATAATAA	0.89	0.93	0.20
TD094-626	ATA	---	TCGATGGATA	ATAATAATAA	0.94	1.00	0.60
TD095-15	G	A	GGTGAGCATC	TGCTCTGTTC	0.92	0.94	0.91
TD095-249	G	C	TGCTACGTAC	AAATGATAGG	0.84	0.88	0.18
TD095-329	G	A	GAATATGCTG	TAGAGGTGTT	0.97	1.00	0.73
TD096-184	T	C	CAAGAAATGT	GTTTTTGATA	0.89	0.94	0.40
TD096-205	G	T	TTTTTACAGG	AAGGGTCAAG	0.94	0.94	0.40
TD096-259	G	A	GGAAAATGAG	AGGATTATGA	0.89	0.94	0.40
TD096-313	G	T	AGTATAGAGG	GGGTGGGAGT	0.89	0.94	0.40
TD096-328	G	A	GGGAGTCTGA	GCTGCCAGTG	0.92	0.94	0.40
TD096-340	A	T	CTGCCAGTGT	GTTGAGGATG	0.92	0.94	0.40
TD096-376	A	G	AATCTGCTAC	AATGGGATTG	0.93	0.94	0.40
TD096-592	T	C	ACTTGAAGAT	TGTAAGGAGG	0.90	0.94	0.40
TD100-115	T	C	GTTTGCATTT	TTTTTACAAA	0.94	1.00	0.82
TD100-121	C	-	TTTTTTTTTA	AAAAAAGAAT	0.91	1.00	0.64
TD100-172	A	T	ATTCTGAAGT	GATGGTCTGA	0.92	1.00	0.64
TD100-176	---------	TATGACACC	AAGTAGATGG	TCTGATGGAA	0.92	1.00	0.64
TD100-179	C	T	AGTAGATGGT	TGATGGAAAA	0.92	1.00	0.64
TD100-212	A	T	CCATAAATGT	ATATGTGGTG	0.92	1.00	0.64
TD100-213	A	T	CATAAATGTA	TATGTGGTGA	0.92	1.00	0.64
TD100-387	A	G	GCGGCAGGTG	GGAGAGGGGG	0.92	1.00	0.64
TD100-388	G	A	CGGCAGGTGA	GAGAGGGGGG	0.94	1.00	0.82
TD100-569	G	T	TAAATGGGGC	TGCTGATGGC	0.92	1.00	0.64
TD100-68	A	T	ACTTAATCCT	TGGGAATTAT	0.91	1.00	0.64
TD102-263	G	A	CTGGTCTGGC	TTTTCCCAAT	0.72	0.88	0.18
TD106-219	C	T	TGAGAACCCA	AAAGGTGCTG	0.93	1.00	0.55
TD108-347	C	A	AGCTATGTAA	GAAAAATATG	0.80	0.92	0.18
TD109-216	T	C	CTCATCTATA	CAGCAGCTCT	0.82	0.92	0.40
TD109-329	T	C	AAATATTTCT	CGTTTAAGAT	0.82	0.91	0.40

polymorphism name [a]	Allele in reference genotype	Second allele	Upstream sequence	Downstream sequence	Frequence of reference allele		
					S. l. cera (N=63)	*S. l. esc* (N=17)	*S. l. pimpi* (N=10)
TD109-404	G	A	ATGCTTGAGA	TTGAGACTAC	0.82	0.91	0.40
TD109-488	A	G	GAGAATAAGG	CATTAGTGCT	0.84	1.00	0.38
TD109-498	T	C	ACATTAGTGC	TTGCAGAATG	0.82	1.00	0.38
TD110-49	T	G	CTGGAGTTGT	GCCTTGCTAG	0.83	1.00	0.45
TD110-8	T	C	TTGTTCT	GAAGATTTGC	0.84	1.00	0.45
TD112-525	G	T	AATTGATTAG	TAGAGTTTTG	0.85	1.00	0.36
TD113-132	G	T	AGTTCCTCTG	TTTTTTTACT	0.84	1.00	0.18
TD113-230	AGTCTTTCC	---------	CTTTGCTTTC	TCTTCCTAGA	0.97	1.00	0.64
TD114-102	TTAGGAGTA ACAATTG	----------------	TGATTGATTA	TTAGGAGTAA	0.85	1.00	0.36
TD114-151	G	A	AGCAAACAAT	CAAGGAAAGA	0.84	1.00	0.30
TD114-166	C	G	GAAAGAATTA	AGTTGTAATT	0.84	1.00	0.30
TD114-203	G	T	TTTTGCCAGT	CTATTGCCTC	0.84	1.00	0.30
TD114-207	T	A	GCCAGTGCTA	TGCCTCTTAA	0.84	1.00	0.30
TD114-217	--------------	ATTCTTTAA GAGAC	TGCCTCTTAA	AGATCATGGG	0.85	1.00	0.30
TD114-259	G	T	GTTAATCTTT	GTATTTTAAT	0.84	1.00	0.30
TD114-359	G	A	GAACCCGTAT	TGCAATGCTA	0.83	1.00	0.30
TD114-373	A	G	AATGCTAGAT	CACCTATAAT	0.83	1.00	0.30
TD114-392	C	T	ATAGCATGAA	ATCTATATAA	0.83	1.00	0.30
TD114-439	C	T	AATGGTAAAC	TGTAGTTTAC	0.83	1.00	0.30
TD114-464	A	G	TGTAGTGTTA	TGTTGCTTAG	0.85	1.00	0.30
TD114-507	C	T	ATCTTAGGTT	GTTGATAATA	0.83	1.00	0.30
TD114-551	T	C	TGCACCTTAA	GCAGAAATAA	0.86	1.00	0.30
TD114-598	G	T	GCCAAAAGTG	GTCGCGTGCC	0.82	1.00	0.30
TD114-604	G	A	AGTGGGTCGC	TGCCTTTAAT	0.82	1.00	0.30
TD114-613	A	C	CGTGCCTTTA	TGATTTTGTT	0.86	1.00	0.30
TD114-635	T	A	ATTATAGATC	ATGTGGCTTC	0.82	1.00	0.30
TD114-638	G	C	ATAGATCTAT	TGGCTTCTGT	0.82	1.00	0.30
TD114-648	T	C	GTGGCTTCTG	TTCGTAGTGG	0.82	1.00	0.30
TD116-260	G	T	TGGGGCTTTT	GCATCACAAT	0.81	0.91	0.10
TD116-283	C	A	TAATTTTCTA	TAAAATAATA	0.77	0.91	0.10
TD116-355	G	A	TTTTTTCCGT	TCTGTGATTT	0.98	1.00	0.90
TD116-393	G	C	TAAAGATCTC	TCTCTTTCGT	0.94	0.91	0.80
TD116-66	C	G	GTGATTTGAA	GTAGGAGGAG	0.89	1.00	0.40
TD116-707	A	G	GTGTCTTACT	CAAGATTCCC	0.59	0.90	0.00
TD116-745	C	T	TCCTCGGATA	GCTACTACTT	0.88	1.00	0.50
TD117-101	C	T	AAGATTTTTC	GTTGATCTAT	0.95	1.00	0.82

polymorphism name [a]	Allele in reference genotype	Second allele	Upstream sequence	Downstream sequence	Frequence of reference allele		
					S. l. cera (N=63)	*S. l. esc* (N=17)	*S. l. pimpi* (N=10)
TD117-164	T	C	ACTGATTTAT	ATCCTAACCA	0.72	1.00	0.09
TD117-176	T	A	TCCTAACCAT	TTATGGTATG	0.72	1.00	0.09
TD117-219	G	A	GGAGATTCTT	AATTGCTTTT	0.93	1.00	0.91
TD117-399	C	T	AGTTGGGTCA	GTTATGTTTA	0.94	1.00	0.80
TD117-420	G	A	TTGACTCAAA	TCTTCTTTGA	0.94	1.00	0.80
TD117-422	C	A	GACTCAAAGT	TTCTTTGACT	0.94	1.00	0.80
TD117-570	A	C	GATGTACAGT	GCTTATATTC	0.83	1.00	0.13
TD117-623	AC	--	TTGATTTATG	ACACTTTCCA	0.87	1.00	0.30
TD117-672	G	A	AATATGATGT	CGTGTCAAA	0.79	1.00	0.10
TD117-699	C	A	GCCAGTGACG	AGCATACTTC	0.79	1.00	0.10
TD120-172	A	T	TGTTTTTGTC	ATCGATGTAA	0.86	0.94	0.64
TD120-212	T	C	TACTTTTAAA	CGGATAAACC	0.69	0.88	0.18
TD120-283	-	T	TCGTTTGGTT	GGTTTGGTTT	0.68	0.88	0.09
TD120-309	C	T	ATTTTAAAAA	CGACTAGATT	0.89	0.87	0.67
TD120-310	C	T	TTTTAAAAAC	GACTAGATTG	0.87	1.00	0.78
TD120-333	A	T	TTTGGTTTTA	TTTTAATCAA	0.93	1.00	0.56
TD120-382	T	A	CCCCTAAATA	AATACGGATT	0.87	1.00	0.67
TD120-418	A	T	TTTTTTTTTT	AATGGTAATT	0.87	1.00	0.67
TD120-444	-	T	ATTATGGTGT	TTTTTAAAAA	0.67	0.85	0.25
TD120-445	-	T	TTATGGTGTT	TTTTAAAAA	0.71	0.85	0.25
TD120-88	G	T	AAACACTTTG	TCGTGTAATG	0.73	0.88	0.09
TD120-90	C	T	ACACTTTGGT	GTGTAATGAT	0.97	1.00	0.82
TD120-93	G	A	CTTTGGTCGT	TAATGATAAC	0.97	1.00	0.73
TD121-124	C	G	ATTTTTATAA	TATATTATTG	0.82	0.88	0.64
TD121-196	C	A	AAAGACGATT	TGTATTTAAG	0.92	1.00	0.91
TD121-218	T	G	GGAAACGAAC	GAGACCTTTT	0.90	0.88	0.82
TD121-236	A	G	TTTAGTTAAA	ATGAAAAATT	0.68	0.88	0.09
TD121-267	T	A	TCACCACAAA	CCTTGTTGGT	0.77	0.88	0.55
TD121-272	G	A	ACAAATCCTT	TTGGTGCGTC	0.93	1.00	0.64
TD121-278	G	A	CCTTGTTGGT	CGTCATTCAT	0.68	0.88	0.09
TD121-286	C	T	GTGCGTCATT	ATTATTAAAT	0.92	1.00	0.60
TD121-384	A	G	TTTAAAAATT	AACTGTTTAG	0.88	0.87	0.90
TD130-117	T	C	GACAAACATA	TGTAACGAGG	0.82	1.00	0.73
TD130-143	G	C	TTAAGATAGA	ATCAATTCCT	0.95	0.80	1.00
TD130-261	G	T	CATGTAACGA	ACTCAGGGAT	0.98	1.00	0.55
TD130-266	A	G	AACGAGACTC	GGGATTTAAA	0.98	1.00	0.55
TD130-430	C	A	GATCAACATC	ATAAATAAAT	0.77	0.93	0.09

polymorphism name [a]	Allele in reference genotype	Second allele	Upstream sequence	Downstream sequence	Frequence of reference allele		
					S. l. cera (N=63)	*S. l. esc* (N=17)	*S. l. pimpi* (N=10)
TD130-44	A	T	GATCTTAAGC	TGTCATATGA	0.77	0.93	0.09
TD132-122	C	A	GCATCTAATT	CACTCATGAG	0.90	1.00	1.00
TD132-167	G	T	AGGCAAAGAT	TTTGTGGATA	0.65	0.88	0.09
TD132-436	G	A	ACCCTAAAAA	GGGGGAAAGT	0.64	0.87	0.09
TD133-115	----------	CTGCGATTTG	AAAGCCTTTG	TTGGAACATT	0.72	0.88	0.09
TD133-305	T	C	TGGAAGTTAT	GTGTATGATT	0.92	1.00	0.91
TD133-390	T	C	TAAACTAGAG	ATGCAGAATC	0.92	1.00	0.91
TD133-395	A	C	TAGAGTATGC	GAATCCAGGA	0.73	0.87	0.09
TD138-114	T	C	TATTAGCTGC	GTATCTTTAG	0.92	1.00	0.73
TD138-121	T	G	TGCTGTATCT	TAGGGGATGG	0.92	1.00	0.64
TD138-123	A	T	CTGTATCTTT	GGGGATGGCG	0.92	1.00	0.73
TD138-28	G	A	TCGACTTTCC	CTGCAGTATT	0.95	1.00	0.82
TD138-39	C	T	CTGCAGTATT	GGACCCATCC	0.95	1.00	0.82
TD138-48	C	A	TCGGACCCAT	CCACATTCAA	0.94	1.00	0.73
TD138-49	C	T	CGGACCCATC	CACATTCAAA	0.94	1.00	0.73
TD138-50	C	T	GGACCCATCC	ACATTCAAAT	0.94	1.00	0.73
TD138-59	A	G	CCACATTCAA	TTGACCGTTA	0.94	1.00	0.73
TD138-61	T	C	ACATTCAAAT	GACCGTTAAT	0.92	1.00	0.73
TD138-62	G	C	CATTCAAATT	ACCGTTAATG	0.94	1.00	0.73
TD139-547	C	T	TCTCATTTTT	GTGAGTGAAG	0.75	1.00	0.27
TD140-123	C	A	TTGTTGGATA	CAAGTAGGAT	0.84	0.93	0.27
TD140-180	T	A	TATTGAACCT	AATCTGATAT	0.84	0.93	0.27
TD140-480	C	A	TGATCCCGAT	TGGACCAATA	0.08	0.31	0.00
TD145-232	A	G	CTATTACTCA	ATTCGTAAAT	0.66	0.88	0.09
TD145-289	T	C	TGGATCTCAG	AAGGTAAGAT	0.66	0.88	0.09
TD145-317	T	A	AACAACTCTT	TGATGATACT	0.73	0.88	0.27
TD145-328	T	A	TGATGATACT	TTCGAAAGAG	0.72	0.88	0.27
TD145-34	T	C	CGCTCTTCTA	TCTACATCTT	0.66	0.88	0.09
TD145-389	A	G	GAGAGATTAT	GCATAGATGA	0.72	0.88	0.27
TD145-45	A	G	TCTACATCTT	CCTCTGCGAA	0.73	0.88	0.27
TD145-454	A	G	TCATTACTCA	AAGAGTGTTT	0.73	0.88	0.27
TD145-466	A	G	AGAGTGTTTT	ATTAGACCTG	0.72	0.88	0.27
TD145-59	T	C	CTGCGAAAGA	TCATTCTCAG	0.73	0.88	0.27
TD145-9	C	G	ATTCATCA	TCCTTTGTTA	0.73	0.88	0.27
TD145-90	T	A	TAGTTTCTCA	ATGTACATTT	0.66	0.88	0.09
TD150-16	------------	ACAGTTCTTATT	TTTCTGATGA	TTATCCGTGC	0.83	1.00	0.30

polymorphism name [a]	Allele in reference genotype	Second allele	Upstream sequence	Downstream sequence	Frequence of reference allele		
					S. l. cera (N=63)	*S. l. esc* (N=17)	*S. l. pimpi* (N=10)
TD150-303	C	A	CTATGCATAT	ATTTATTTTA	0.85	1.00	0.30
TD150-337	G	C	AAAAGCAAAA	TGAGTCTTTT	0.85	1.00	0.30
TD150-531	---	TTC	TGATGATGAC	TTCTTAGAAT	0.71	1.00	0.10
TD187-41	A	G	TTTATATTTT	AATTTTCTTA	0.78	0.86	0.27
TD187-448	--------	TATAGTAG	AGAATATAGA	TAATATAGTA	0.90	1.00	0.73
TD187-472	G	A	GTTACCACTT	CCACATATAA	0.70	0.86	0.09
TD187-48	C	T	TTTAAATTTT	TTAATGAAAA	0.90	1.00	0.82
TD187-51	A	C	AAATTTTCTT	ATGAAAATTT	0.92	1.00	0.82
TD187-65	-	T	AAATTTACAC	TTTTTTTTAC	0.76	0.86	0.36
TD187-73	-	T	ACTTTTTTTT	ACCTCATTTA	0.76	0.86	0.36
TD188-201	G	C	CTTTTTTCTC	GATGTAAAGA	0.85	1.00	1.00
TD265-456	C	T	AATTACAGAT	GACTACTTCC	0.84	1.00	0.82
TD268-161	T	G	TATTTGTTAC	GTTGCAGTTA	0.90	0.94	0.55
TD268-431	G	C	TTCATCATAT	GTCGGAGGAC	0.86	1.00	0.27
TD270-301	G	A	GCTGTCTACC	AAAATATTTC	0.93	1.00	0.70
TD270-312	A	T	AAAATATTTC	CTCTTGAGGT	0.93	1.00	0.70
TD272-104	A	G	ACAAGGCGAT	AGAGAAGTCC	0.92	1.00	0.82
TD272-277	C	T	AATGGTGGTT	GTATTTTAAC	0.77	0.93	0.56
TD274-17	A	T	AGGTATTTAT	TTGTCTTAGT	0.79	0.94	0.18
TD274-222	C	G	TAAGTATCTA	AGATTGATAA	0.91	0.94	0.50
TD274-325	A	C	GAAAACACTT	CCTTCCTACC	0.83	0.94	0.20
TD274-38	C	T	ACATTTATTT	CGTTTTTTTC	0.95	1.00	0.73
TD275-240	C	T	AAATGCGAGA	CTTTATCTAA	0.89	0.94	0.64
TD276-20	C	T	ATTTTCTTTT	TTTTTCAGTT	0.89	1.00	0.38
TD276-97	T	C	GATGCCAAGA	GTGAGTTACA	0.80	1.00	0.13
TD278-21	G	A	ATCTTTATGA	TACAATCAGA	0.86	1.00	0.90
TD278-267	G	A	GTTAGCCAAC	TTAACCCTGT	0.78	1.00	0.55
TD278-39	A	G	AGAAGGAACG	AGAACTGTAC	0.80	1.00	0.90
TD278-444	A	G	TAGTTAGGTA	CCAACACTAT	0.77	1.00	0.50
TD278-524	C	T	TGTAGACGCT	GATCTTTCTC	0.79	1.00	0.50
TD279-253	C	A	CATTACTAGG	CAAACAAGAA	0.84	0.94	0.55
TD280-328	T	C	CTTTCGTTGG	ATCTAGCGTG	0.49	0.80	0.00
TD300-175	------------	TAATAATAA TAA	AGAGAAATAA	TAATAATAAT	0.81	0.92	0.40
TD300-257	G	A	GACAAAAGCA	AAAGAGAAAA	0.83	1.00	0.33
TD300-41	T	C	CACTCATATT	TAGAAATTTT	0.70	0.92	0.11
TD300-47	A	G	TATTTTAGAA	TTTTAAATTC	0.91	1.00	0.63

polymorphism name [a]	Allele in reference genotype	Second allele	Upstream sequence	Downstream sequence	Frequence of reference allele		
					S. l. cera (N=63)	*S. l. esc* (N=17)	*S. l. pimpi* (N=10)
TD304-235	A	G	CTCTACTGTT	TTGGAAGCTT	0.85	1.00	0.50
TD304-322	T	C	TATGGATCAA	CCTTTCGGAT	0.13	0.07	0.91
TD304-453	A	T	ACATAGTATA	TGAAAACAAC	0.85	1.00	0.45
TD304-514	T	G	CAAACCATGT	TTTATTTAAC	0.85	1.00	0.45
TD304-524	C	T	TTTTATTTAA	TAGGGAAGTG	0.88	1.00	0.60
TD305-236	T	C	GTTTAACAGA	TTGTAATGAT	0.90	0.93	0.40
TD305-355	T	A	AGAAGTTATT	TAGTATTGAC	0.81	0.80	0.10
TD316-112	G	C	ATTGATTGTT	TTGATTTTGC	0.75	0.88	0.11
TD316-143	C	T	ATGACCTAGA	AGATCGAGAT	0.75	0.88	0.11
TD316-197	T	A	TTTTTCTTGT	TCTTGTGTAG	0.77	0.88	0.11
TD316-206	A	G	TTTCTTGTGT	GCACAACTTC	0.77	0.88	0.11
TD316-23	A	T	AGTATGTAGT	GTTCTCTTTT	0.75	0.88	0.20
TD316-256	G	A	GTCTAATTTT	ACCTTGATAT	0.77	0.88	0.11
TD316-259	C	-	TAATTTTGAC	TTGATATGGA	0.77	0.88	0.11
TD316-268	G	A	CCTTGATATG	AGTGTTTAAA	0.77	0.88	0.11
TD316-269	A	C	CTTGATATGG	GTGTTTAAAA	0.77	0.88	0.11
TD316-270	G	T	TTGATATGGA	TGTTTAAAAG	0.77	0.88	0.11
TD316-272	G	T	GATATGGAGT	TTTAAAAGTA	0.77	0.88	0.11
TD316-274	T	A	TATGGAGTGT	TAAAAGTAAA	0.77	0.88	0.11
TD316-275	T	A	ATGGAGTGTT	AAAAGTAAAG	0.77	0.88	0.11
TD316-28	T	C	GTAGTAGTTC	CTTTTTTCTA	0.75	0.88	0.20
TD316-364	T	C	AAAAGTTGGA	TGAAGAGTGC	0.77	0.88	0.11
TD316-407	CAA	---	TTTGAAACAA	ACAAATTGAA	0.87	0.94	0.63
TD316-62	T	G	ATCTTTTACT	CGATTTGTTA	0.75	0.88	0.11
TD316-63	C	T	TCTTTTACTT	GATTTGTTAT	0.78	0.88	0.20
TD316-669	T	A	TATGGATGGA	TGAGATTATC	0.87	0.94	0.63
TD316-77	T	-	TTGTTATTTT	AGTCAACCCC	0.75	0.88	0.11
TD328-318	T	-	AAATGAATGA	GAATTCTCAT	0.91	0.94	0.50
TD328-95	G	T	CTCTATTTCT	ATTTCAATTG	0.90	0.94	0.50
TD339-293	A	C	GATTAATTAG	TAATTTCTCC	0.98	1.00	0.90
TD339-308	G	C	TTCTCCTTCA	TTCCAGTATA	0.98	1.00	0.90
TD339-321	C	T	CCAGTATATA	GACTGTTTGA	0.97	1.00	1.00
TD339-358	T	A	CAAAGTTAAT	TGAAGTGAAT	0.89	1.00	0.90
TD339-389	T	C	GGATTTAACA	AATTTTCAGA	0.89	1.00	0.90
TD339-81	G	A	TGTTGAATTC	TATATTCGAC	0.87	1.00	0.82
TD343-102	-	T	TCTGTTTTTT	CCCTATGTAG	0.71	0.94	0.09
TD343-133	T	-	TATTTTTTTT	CCTGGAATTA	0.86	0.94	0.55

polymorphism name [a]	Allele in reference genotype	Second allele	Upstream sequence	Downstream sequence	Frequence of reference allele		
					S. l. cera (N=63)	*S. l. esc* (N=17)	*S. l. pimpi* (N=10)
TD343-175	G	T	TTCCTCACTT	CTCTGATGAT	0.91	1.00	1.00
TD345-138	A	C	TCGGCAAGGA	GTTACTCGCT	0.94	1.00	0.82
TD345-195	A	T	CCATTTTTTT	AAAAAAGATG	0.89	1.00	0.82
TD345-208	A	T	AAAAGATGTC	ACTTTATAAT	0.94	1.00	0.80
TD345-241	A	T	TTAATTGTTG	TTGTTGTTTA	0.70	0.93	0.09
TD345-253	T	C	TGTTGTTTAT	CTCTTCATGT	0.94	1.00	0.82
TD345-463	TTTTAA	------	AAAGAGATAC	TTTTAATTTT	0.94	1.00	0.80
TD348-300	C	T	GTTAATTTTC	TTATTGAGTT	0.81	1.00	0.22
TD348-71	A	C	TCTTTCAAAG	CTTCCTCTGT	0.97	1.00	0.73
TD348-72	-	T	CTTTCAAAGA	CTTCCTCTGT	0.97	1.00	0.73
TD363-126	T	G	GAGTAAATAT	TCATTTTATA	0.83	0.93	0.90
TD363-170	A	T	GATAGACTAA	GGAATAATTG	0.79	1.00	0.30
TD363-213	C	T	TTTGTTAACG	ATAGAATCAA	0.61	0.93	0.11
TD363-241	A	T	TAGGACATGT	AAATGGGAGT	0.82	1.00	0.89
TD363-486	G	A	CATAGGAGTG	GGTTTTTACC	0.83	0.93	0.89
TD363-498	C	A	GTTTTTACCT	GTGCGCACTC	0.83	1.00	0.89
TD363-499	G	T	TTTTTACCTC	TGCGCACTCA	0.83	0.93	0.89
TD363-517	G	C	TCAAAGGGTA	CAGCTGTGGA	0.96	1.00	0.78
TD363-531	C	T	CTGTGGATTT	CCTTGATGTA	0.83	0.93	0.89
TD363-542	T	A	CCTTGATGTA	AAAAA	0.64	0.93	0.11
TD369-146	A	G	TGTAGGTACA	ATGAATATTG	0.97	1.00	1.00
TD369-23	A	-	GAGTGCTTCC	AAAATCACTG	0.93	1.00	0.91
TD369-328	T	C	TATGCCTTCC	TAGTGAAACT	0.86	1.00	0.20
TD369-340	A	G	AGTGAAACTG	AAAGTTTCAC	0.96	1.00	1.00
TD369-383	G	A	GAGATGATAG	TTTTTGTTAA	0.96	1.00	1.00
TD369-430	T	A	AAAAACTTCT	TATCTTTCAA	0.87	1.00	0.30
TD369-493	G	A	TTATAAGGAA	TCGTTGAGTA	0.77	1.00	0.11
TD373-140	T	C	CCTACATTCT	AAACCTTTTA	0.77	0.86	0.18
TD373-391	G	T	TGTTCTAATT	GGTTGATTAA	0.42	0.50	0.91
TD375-386	TTGAGCTAAT	----------	TTTAGCTAAT	GGATTGAGTG	0.94	1.00	0.90
TD375-573	T	C	ACATCTCTGG	GCAATCCTCT	0.78	0.93	0.30
TD375-98	G	T	CATGTAGTAC	AAGTGTCATA	0.86	0.94	0.70
TD377-91	-	T	GGTGGACTTA	TTTTAAGTAT	0.90	1.00	0.20
TD377-96	A	T	GACTTATTTT	AGTATTTTTT	0.90	1.00	0.20
TD377-97	A	T	ACTTATTTTA	GTATTTTTTT	0.90	1.00	0.30
TD377-98	G	T	CTTATTTTAA	ATTTTTTTTT	0.90	1.00	0.20

polymorphism name [a]	Allele in reference genotype	Second allele	Upstream sequence	Downstream sequence	Frequence of reference allele		
					S. l. cera (N=63)	*S. l. esc* (N=17)	*S. l. pimpi* (N=10)
TD379-180	-	A	AGAATTAAAA	TCATTATTTT	0.90	0.94	0.11
TD379-219	-	T	ATTATTATTA	TATATATACC	1.00	1.00	0.56
TD379-326	C	T	TATTGCAATT	GAAAAGGTTA	0.80	1.00	0.89
TD379-353	A	G	TTCCTATACT	TTCAAAAAGG	0.90	0.93	0.56
TD379-483	A	C	TTTTCCTAAC	CACTCAACCT	0.91	0.93	0.13
TD380-242	C	T	TAACCAACAA	ACCTACAAAC	1.00	1.00	0.82
TD380-256	A	G	TACAAACTAC	AAGTATACTT	0.85	0.94	0.20
TD380-526	T	C	AAAATATAGG	AACTCAGTAA	0.69	0.88	0.00
TD381-516	G	A	TTCAAATCTT	AAAATAAAAA	0.95	0.93	1.00
TD381-548	C	T	GCATAAAGGA	ACAGAATTTT	0.94	0.93	0.50
TD381-568	C	T	TAGAATTTCA	GTATTAATTT	0.94	0.93	1.00
TD381-8	C	T	TTGTCAC	CTTTTGTCTT	0.76	0.93	0.20
TD382-102	C	G	AGACATGCAT	ACTATACTTG	0.93	0.88	1.00
TD382-172	T	C	ACGTGTTTTG	TGATCTTGTG	0.81	0.86	0.44
TD382-251	T	A	ATACATAGAT	TGCCCTTAAA	0.92	0.88	0.82
TD382-37	G	A	TGCATGGACA	ATATGTCCCT	0.79	0.81	0.64
TD383-419	A	G	TACTAGAGAG	GTGTTTTTGT	0.92	1.00	0.33
TD383-558	A	G	AAATTGTATG	ACAAACATTA	0.92	1.00	0.17
TD383-60	A	G	CCATGTGTTT	GCCATAAAAT	0.92	1.00	0.17
TD383-679	T	C	TACAATTTTA	CTTTTAACTT	0.85	0.88	0.60
TD383-684	T	-	TTTTATCTTT	AACTTTAAAA	0.84	0.88	0.60
TD385-209	T	A	TCAAACCAAG	AAAGCATCAA	0.73	0.88	0.09
TD385-613	T	C	GAGAATGTTA	ACATGGATGT	0.74	0.86	0.11
TD386-130	C	G	ATTTAATTAA	AAGTAATTTT	0.88	0.83	0.44
TD386-201	A	G	AGAGAAGCAC	GGTCCTCAAT	0.90	0.83	0.44
TD387-339	G	A	ATCTGCTTTG	TATTTCTTGT	0.93	1.00	0.82
TD387-452	C	T	AACTGTCAAA	CATGTGTAGA	0.78	1.00	0.10

[a] Polymorphism name are described with the 'name of the fragment' dash 'position of the SNP'.
[b] Reference allele is allele of the sequence deposited at GenBank

Table S5 Significant associations detected when analysing only the 63 cherry tomato accessions with the MLM model involving either the structure based on SSR markers (Qssr) or the STS markers (Qsnp). Only associations with corrected p-values <0.5 with either model are shown.

		MLM K+Q (Qssr)		MLM K+Q (Qsnp)	
**trait**	**Locus**	**pvalue**	**corrected p-value**	**pvalue**	**corrected p-value**
**FW**	TD380-526	$4.43X10^{-05}$	0.016	0.0055	ns
	TD056-134	$3.56X10^{-04}$	0.036	0.0026	ns
	TD116-707	$4.07X10^{-04}$	0.037	0.0040	ns
	TD117-219	$2.11X10^{-04}$	0.038	0.0023	ns
	TD138-61	$6.04X10^{-04}$	0.043	0.0425	ns
**LCN**	lcn2.1-692	$9.31X10^{-10}$	$3.43X10^{-07}$	$2.17x10^{-11}$	$8.03x10^{-09}$
	lcn2.1-686	$8.77X10^{-09}$	$1.61X10^{-06}$	$1.66x10^{-10}$	$3.08x10^{-08}$
**SSC**	TD120-90	$8.35X10^{-04}$	ns	$2.44x10^{-04}$	0.044
	TD120-93	$8.35X10^{-04}$	ns	$2.44x10^{-04}$	0.044
	TD380-526	$6.61X10^{-04}$	ns	$4.14x10^{-04}$	0.049

ns: non significant (p > 0.05)
