# Supplementary material for: Genome-Wide Association Mapping in Tomato (Solanum lycopersicum) Is Possible Using Genome Admixture of Solanum lycopersicum var. cerasiforme
Source: G3 (Bethesda). 2012 Aug 1;2(8):853–64. doi: 10.1534/g3.112.002667 (PMC3411241; doi:10.1534/g3.112.002667)
Supplement: Supporting Information [file supp_2.8.853_FigureS1.pdf]

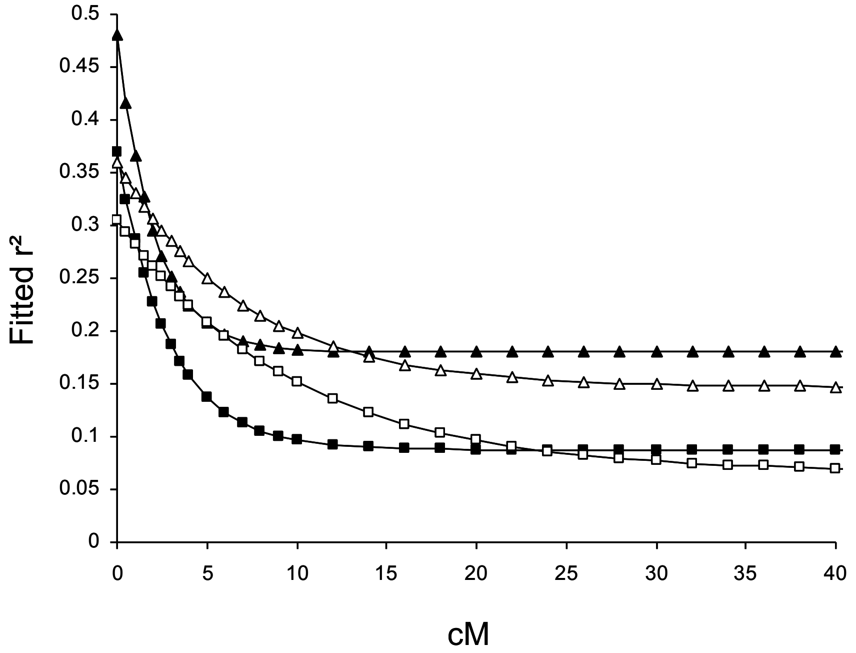

Figure S1 Comparison of different models for the analysis of linkage disequilibrium decay over genetic distances. Whole collection and *S. l. cerasiforme* collection are represented by triangle and square, respectively. One polymorphic site per fragment and all sites with MAF>5% survey strategies are represented in white and black, respectively.
