# Supplementary material for: Genome-Wide Association Mapping in Tomato (Solanum lycopersicum) Is Possible Using Genome Admixture of Solanum lycopersicum var. cerasiforme
Source: G3 (Bethesda). 2012 Aug 1;2(8):853–64. doi: 10.1534/g3.112.002667 (PMC3411241; doi:10.1534/g3.112.002667)
Supplement: Supporting Information [file supp_2.8.853_FigureS2.pdf]

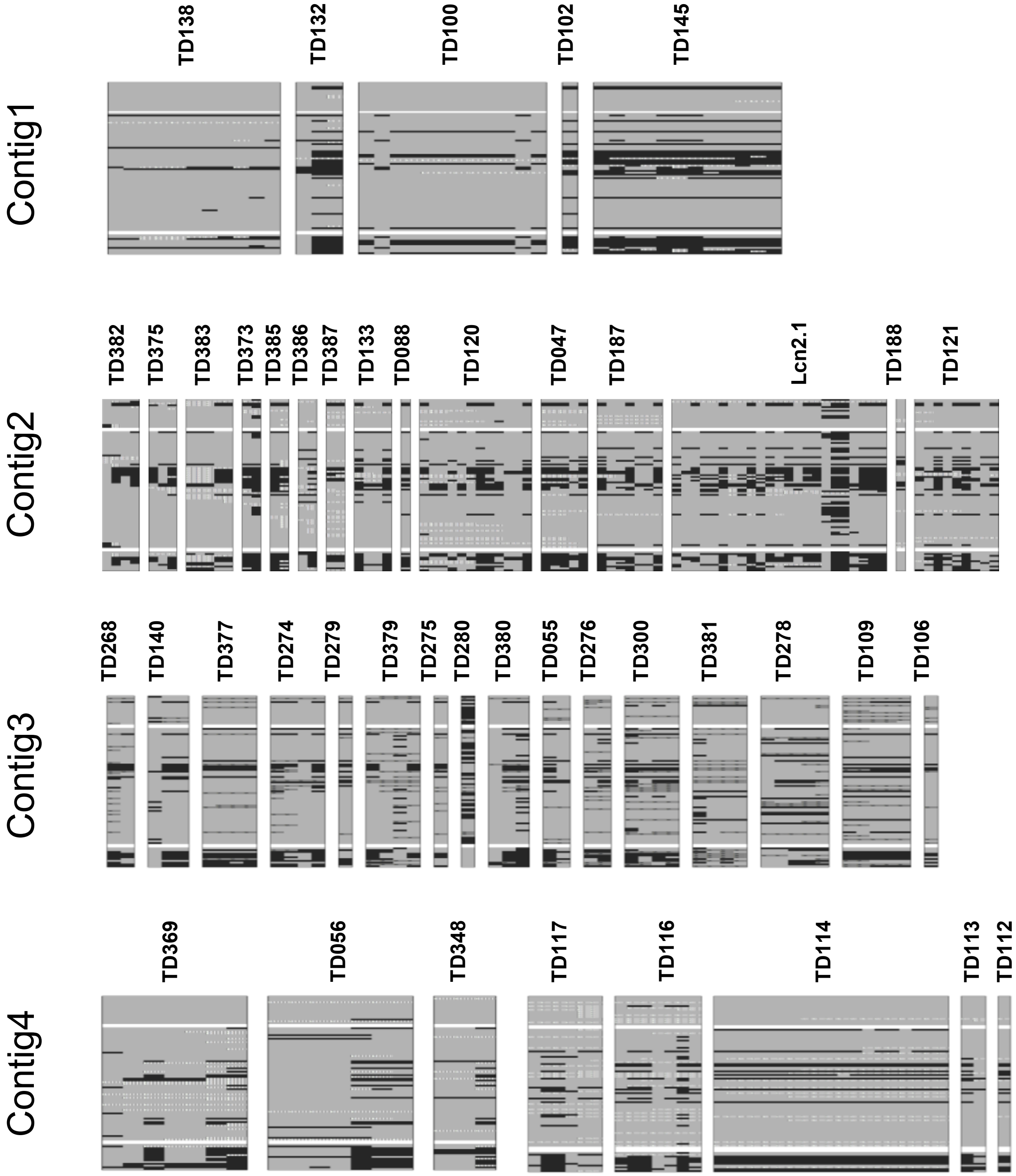

Figure S2 Graphical haplotypes of 90 accessions for markers located on physical contigs. Rows represent accessions and columns represent polymorphic sites. Fragment are separated by white rows. For each polymorphic site, most frequent allele is represented in light gray and the other allele is represented in black. Data failed are represented in white. The three species *S. lycopersicum, S. l. cerasiforme* and *S. pimpinellifolium* are separated by continuous white lines.
