# Supplementary material for: Genome-Wide Association Mapping in Tomato (Solanum lycopersicum) Is Possible Using Genome Admixture of Solanum lycopersicum var. cerasiforme
Source: G3 (Bethesda). 2012 Aug 1;2(8):853–64. doi: 10.1534/g3.112.002667 (PMC3411241; doi:10.1534/g3.112.002667)
Supplement: Supporting Information [file supp_2.8.853_FigureS3.pdf]

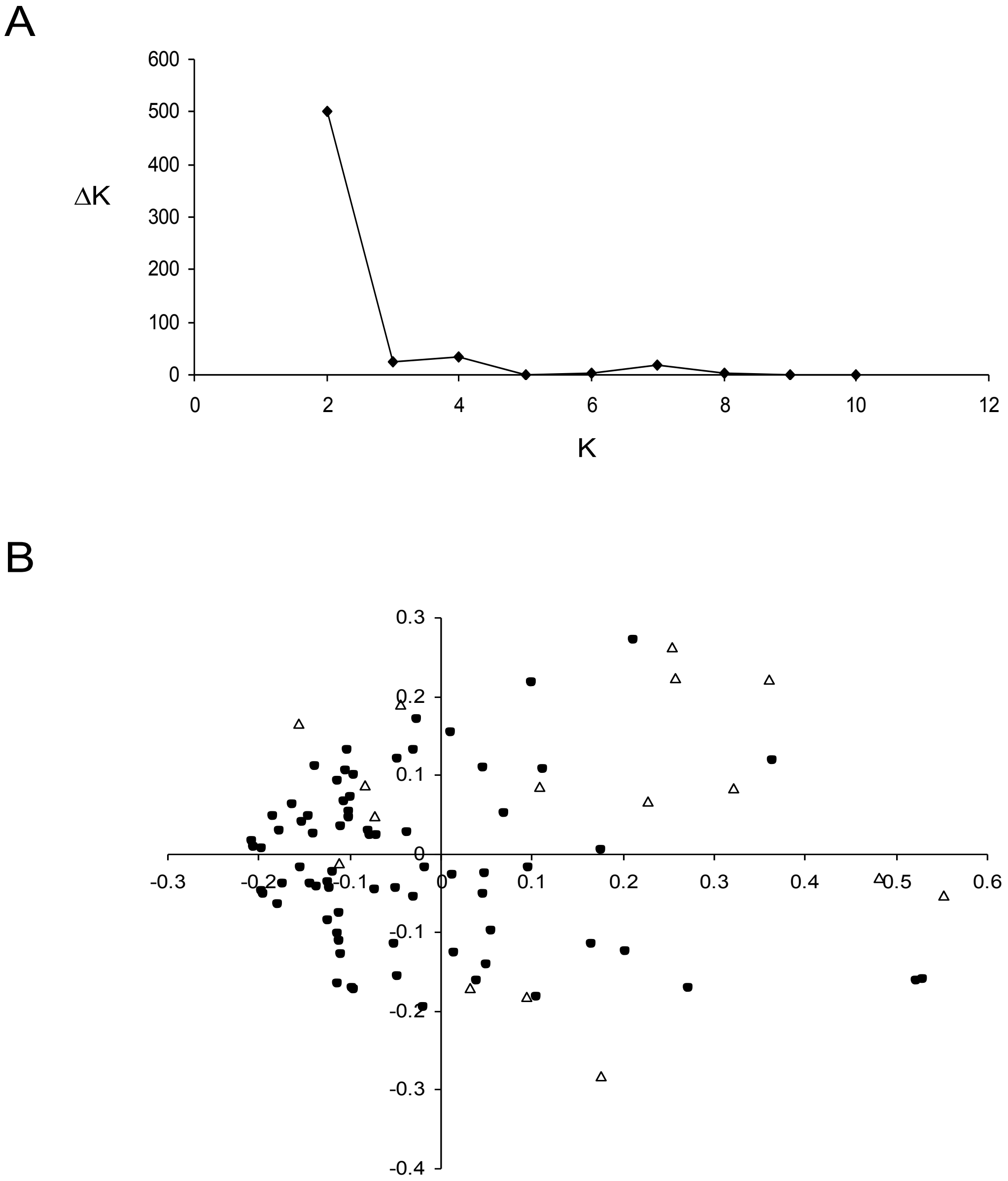

Figure S3 Genetic structure determination of the 90 accessions of wild and cultivated tomato. (A) Determination of the optimal number of sub-population (K) for 90 accessions following the method of Evanno et al. (2005). The rate of change of the posterior probability of the data given the number of clusters is plotted against K, the number of clusters. ΔK was calculated as |L"(K)|/s[Pr(x|k)] (see Materials and Methods). The first peak (K = 2) corresponds to the optimum number of clusters. (B) Principal coordinate analysis of the 90 accessions based on 20 SSR markers. The two groups identified by Structure software are represented by black square and white triangle.
