# Supplementary material for: Genome-Wide Association Mapping in Tomato (Solanum lycopersicum) Is Possible Using Genome Admixture of Solanum lycopersicum var. cerasiforme
Source: G3 (Bethesda). 2012 Aug 1;2(8):853–64. doi: 10.1534/g3.112.002667 (PMC3411241; doi:10.1534/g3.112.002667)
Supplement: Supporting Information [file supp_2.8.853_FigureS4.pdf]

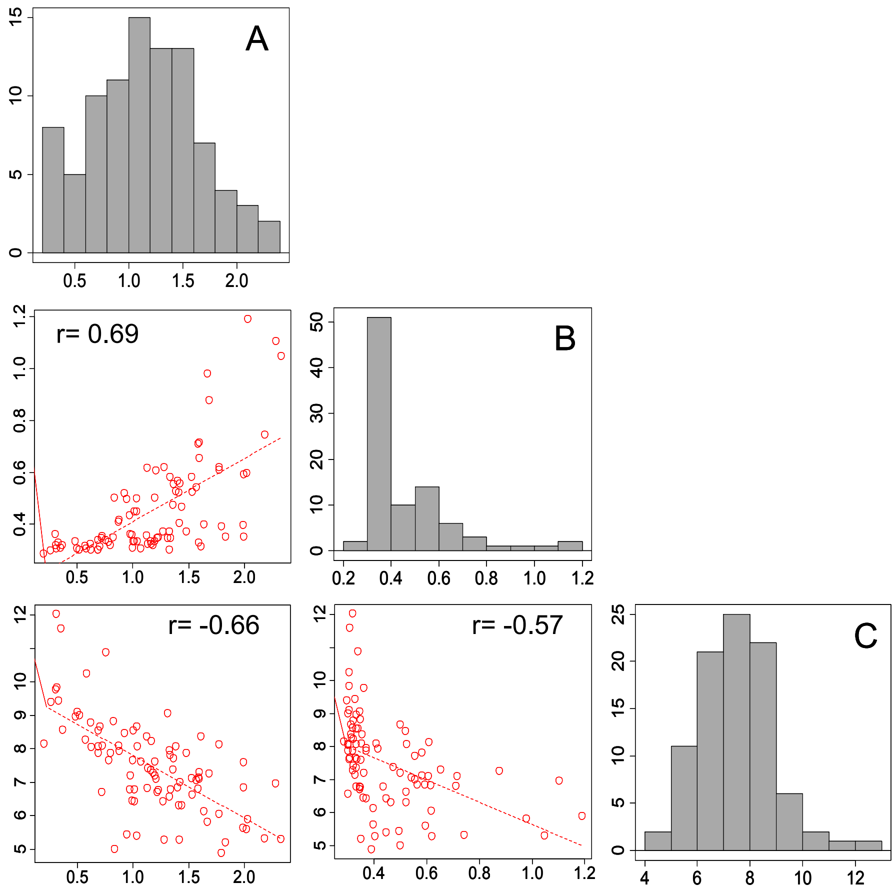

Figure S4 Distribution and correlation of fruit weight (FW), locule number (LCN) and soluble solid content (SSC) for the 90 accessions. Adjusted mean over two year of experiment for each accessions are used to show histogram distribution of traits for logarithm of Fruit Weight in gram (A), logarithm of Locule Number (B) and Soluble Solid Content in °brix (C). Scatter plot diagrams show correlation between traits and Spearman's rank correlation coefficient are indicated.
