# Supplementary material for: Genome-Wide Association Mapping in Tomato (Solanum lycopersicum) Is Possible Using Genome Admixture of Solanum lycopersicum var. cerasiforme
Source: G3 (Bethesda). 2012 Aug 1;2(8):853–64. doi: 10.1534/g3.112.002667 (PMC3411241; doi:10.1534/g3.112.002667)
Supplement: Supporting Information [file supp_2.8.853_FigureS5.pdf]

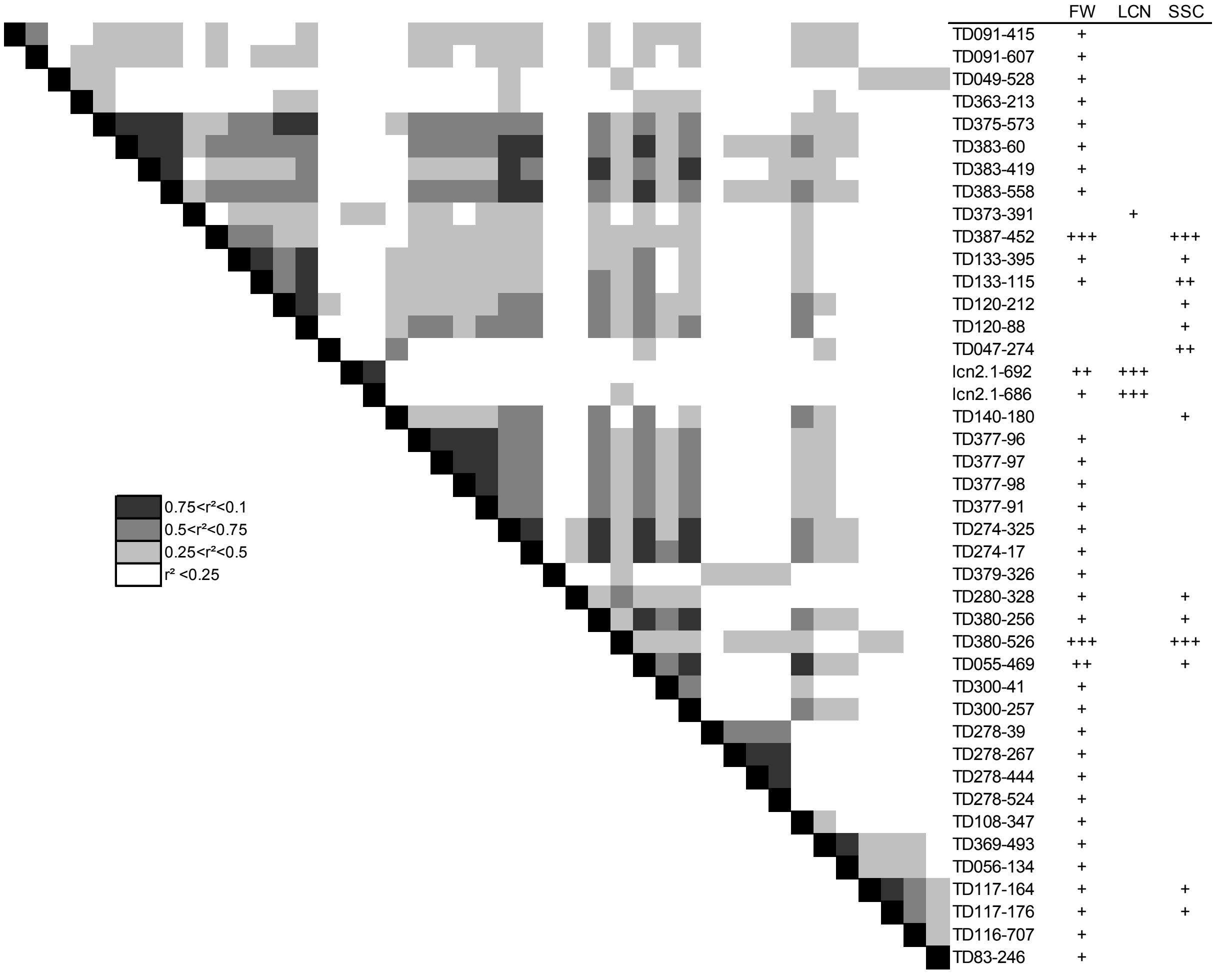


**Figure S5** Matrix of linkage disequilibrium between markers significantly associated with fruit weight (FW), fruit locule number (LCN) and soluble solids content (SSC). Significant associations are indicated with the following symbol: +: <0.001; ++: <0.0001; +++: <0.00001.
