# Supplementary material for: Genome-Wide Association Mapping in Tomato (Solanum lycopersicum) Is Possible Using Genome Admixture of Solanum lycopersicum var. cerasiforme
Source: G3 (Bethesda). 2012 Aug 1;2(8):853–64. doi: 10.1534/g3.112.002667 (PMC3411241; doi:10.1534/g3.112.002667)
Supplement: Supporting Information [file supp_2.8.853_FigureS6.pdf]

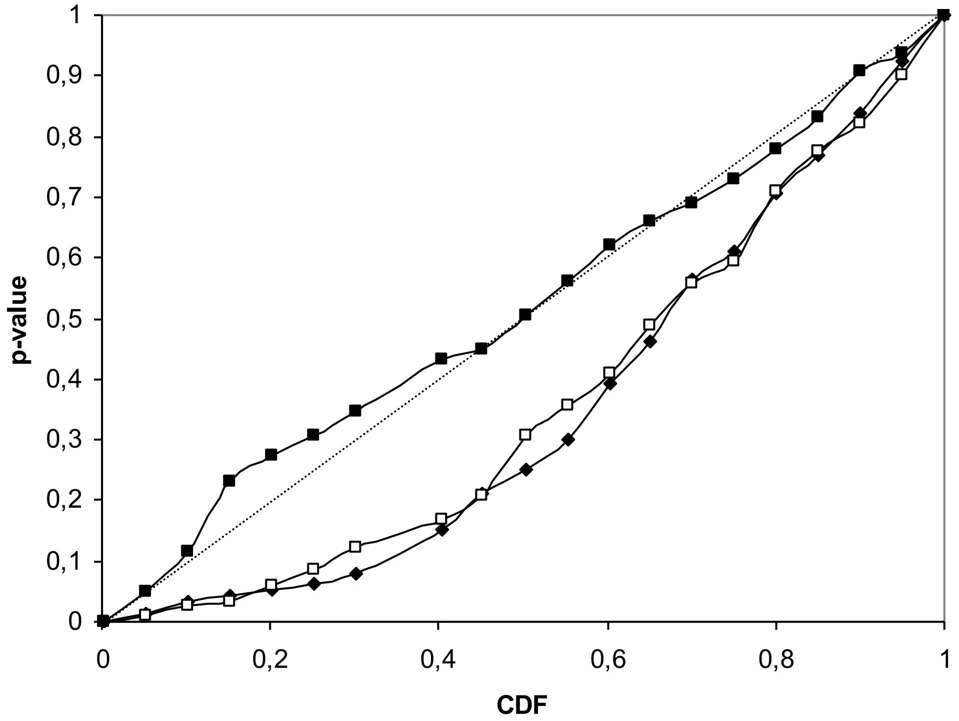

Figure S6 Cumulative density functions (CDF) using several alternative models of association for fruit weight on the subset of 63 cerasiforme tomato. Associations are tested for all polymorphic sites with MAF>5% on 63 individuals. Naive GLM (black diamond) and K+Q models, with structure based on SSR markers (white squares), on 4 PCA axis (white circles) and on all STS markers (black squares) were tested. The diagonal indicates uniform distribution of *p*-values under the expectation that random SNPs are unlinked to the polymorphisms controlling these traits (H0: no SNP effect).
