# Supplementary material for: Genome-Wide Association Mapping in Tomato (Solanum lycopersicum) Is Possible Using Genome Admixture of Solanum lycopersicum var. cerasiforme
Source: G3 (Bethesda). 2012 Aug 1;2(8):853–64. doi: 10.1534/g3.112.002667 (PMC3411241; doi:10.1534/g3.112.002667)
Supplement: Supporting Information [file supp_2.8.853_TableS1.pdf]

Table S1 Accessions used in the association study

Accession Number	Accession Name	Species [a]	traits [c]			STRUCTURE results [c]	
			FW (g)	LCN	SSC (°brix)	pop1	pop2
CR001	Cervil	*S. l. cera*	5.80	2.18	10.88	0.393	0.607
CR002	Levovil	*S. l. esc*	109.13	3.91	6.83	0.188	0.812
CR003	Ferum	*S. l. esc*	109.66	2.24	7.59	0.092	0.908
CR004	M-82	*S. l. esc*	62.60	2.45	4.88	0.04	0.96
CR014	Clémentine	*S. l. cera*	5.40	2.25	8.08	0.012	0.988
CR020	San Marzano	*S. l. esc*	70.00	2.25	5.20	0.01	0.99
CR028	Plovdiv XXIVa	*S. l. cera*	41.58	2.05	8.35	0.012	0.988
CR031	Microtom	*S. l. esc*	6.91	3.17	5.00	0.017	0.983
CR032	Moneymaker	*S. l. esc*	99.54	2.49	5.63	0.008	0.992
CR056	Wva 700	*S. l. cera*	4.25	2.10	8.77	0.015	0.985
CR058	Wva 106	*S. l. cera*	9.69	2.72	6.78	0.99	0.01
CR062	LA 1478	*S. pimpi*	2.05	2.30	9.77	0.459	0.541
CR068	N° 108 Red Currant	*S. pimpi*	2.00	1.98	9.38	0.363	0.637
CR070	N° 2909 Lycopersicon sp.	*S. l. cera*	5.12	2.05	8.65	0.989	0.011
CR072	N° 2921 Lyc. Pimpinellifolium	*S. pimpi*	2.15	2.02	9.82	0.438	0.562
CR075	N° 4156 Blumen Strauss	*S. pimpi*	1.66	1.93	8.13	0.791	0.209
CR076	N° 135 Green Gage	*S. l. cera*	39.90	2.13	7.13	0.157	0.843
CR077	N°1565	*S. l. cera*	10.19	2.28	6.43	0.008	0.992
CR078	N° 2759 Enano	*S. l. cera*	34.84	3.33	6.62	0.135	0.865
CR079	N° 933	*S. l. cera*	33.64	3.82	7.12	0.009	0.991
CR093	N° 2257 Dikorastushii...	*S. l. cera*	23.55	3.58	7.70	0.058	0.942
CR094	N° 1011 Srednei Velichiny	*S. l. esc*	23.30	2.98	7.37	0.008	0.992
CR097	N° 347 Yablochnyi	*S. l. cera*	30.49	2.35	7.87	0.022	0.978
CR098	N° 795 Pescio	*S. l. cera*	22.11	3.82	7.80	0.015	0.985
CR101	N° 884 Alagabotskii	*S. l. cera*	24.49	3.35	8.07	0.02	0.98
CR102	N° 739	*S. l. cera*	54.13	7.52	7.25	0.412	0.588
CR106	LA 1025	*S. l. cera*	15.08	2.10	8.22	0.478	0.522
CR108	LA 1231	*S. l. cera*	4.99	2.18	8.53	0.346	0.654
CR110	LA 1307	*S. l. cera*	14.75	2.15	7.35	0.286	0.714
CR117	LA 1388	*S. l. cera*	16.89	4.05	7.10	0.009	0.991
CR118	LA 1420	*S. l. cera*	39.68	5.13	6.80	0.962	0.038

Accession Number	Accession Name	Species [a]	traits [c]			STRUCTURE results [c]	
			FW (g)	LCN	SSC (°brix)	pop1	pop2
CR122	LA 1456	*S. l. cera*	4.96	2.00	8.07	0.01	0.99
CR123	LA 1461	*S. l. cera*	3.93	2.02	10.23	0.332	0.668
CR124	LA 1464	*S. l. cera*	3.25	2.02	9.10	0.008	0.992
CR125	LA 1482	*S. l. cera*	9.66	2.30	7.20	0.023	0.977
CR129	LA 0147	*S. l. esc*	116.77	3.94	5.58	0.072	0.928
CR130	LA 0172	*S. l. cera*	37.68	3.48	7.05	0.398	0.602
CR133	LA 0409	*S. l. esc*	116.72	15.48	5.88	0.011	0.989
CR134	LA 0466	*S. l. esc*	208.89	12.71	6.95	0.039	0.961
CR136	LA 0473	*S. l. esc*	49.89	9.53	5.80	0.009	0.991
CR145	LA 1543	*S. l. cera*	11.27	2.15	8.07	0.053	0.947
CR149	LA 2095	*S. l. cera*	26.90	3.60	7.00	0.419	0.581
CR150	LA 2131	*S. l. cera*	40.36	4.51	7.28	0.025	0.975
CR152	LA 2307	*S. l. cera*	26.00	3.33	6.30	0.38	0.62
CR153	LA 2308	*S. l. cera*	27.70	2.92	6.30	0.01	0.99
CR155	LA 2402	*S. l. cera*	6.77	2.23	8.82	0.009	0.991
CR156	LA 2619	*S. l. cera*	13.77	4.13	6.82	0.93	0.07
CR158	LA 2675	*S. l. cera*	4.99	2.00	7.87	0.009	0.991
CR159	LA 2688	*S. l. cera*	4.34	2.00	8.03	0.467	0.533
CR163	LA 0400	*S. pimpi*	2.10	2.08	12.02	0.973	0.027
CR164	LA 0411	*S. pimpi*	3.14	2.15	8.95	0.884	0.116
CR169	LA 1371	*S. pimpi*	2.30	2.03	11.58	0.988	0.012
CR173	LA 1547	*S. pimpi*	3.42	2.00	8.98	0.992	0.008
CR186	LA 1689	*S. pimpi*	2.20	2.13	9.43	0.992	0.008
CR199	tomate Richter's	*S. l. cera*	3.82	2.07	8.27	0.988	0.012
CR202	CGN 18399	*S. l. cera*	6.45	2.08	7.87	0.985	0.015
CR203	LA 1589	*S. pimpi*	2.40	2.08	8.57	0.991	0.009
CR205	L. pimpinellifolium atypique, site 10 (F300045)	*S. l. cera*	10.42	2.15	6.78	0.994	0.006
CR234	Atom	*S. l. cera*	26.50	2.53	5.27	0.186	0.814
CR236	PI 365923	*S. l. cera*	15.32	2.08	7.27	0.057	0.943
CR238	PI 129088	*S. l. cera*	12.33	3.15	8.65	0.46	0.54
CR240	L 285	*S. l. cera*	15.96	2.16	7.60	0.158	0.842
CR244	Yellow Pear	*S. l. cera*	19.05	2.35	6.42	0.018	0.982

Accession Number	Accession Name	Species [a]	traits [c]			STRUCTURE results [c]	
			FW (g)	LCN	SSC (°brix)	pop1	pop2
CR249	Cherry Gold	*S. l. cera*	7.53	2.56	8.09	0.71	0.29
CR250	Cherry VFNT	*S. l. cera*	21.60	2.00	6.57	0.306	0.694
CR252	Droplet	*S. l. cera*	16.71	2.22	6.70	0.205	0.795
CR253	Monplaisir	*S. l. cera*	22.49	2.22	6.77	0.275	0.725
CR254	Farthest North	*S. l. cera*	8.91	3.13	5.43	0.375	0.625
CR256	Minibel	*S. l. cera*	19.45	4.17	5.27	0.029	0.971
CR258	Ohmiya Suncherry	*S. l. cera*	13.82	2.10	7.40	0.44	0.56
CR267	Tiny tim	*S. l. cera*	11.08	2.80	5.38	0.292	0.708
CR271	Celsior	*S. l. cera*	12.12	2.02	7.60	0.013	0.987
CR273	Orange Cocktail	*S. l. esc*	60.71	4.07	8.12	0.247	0.753
CR274	Marpha n°2	*S. l. cera*	8.54	3.30	8.47	0.009	0.991
CR275	Cerise Ildi	*S. l. cera*	7.65	2.60	7.92	0.008	0.992
CR279	Cerise Orange d'Uzès	*S. l. cera*	13.82	2.27	8.37	0.21	0.79
CR280	Cerise du sud ouest n° 2	*S. l. cera*	10.22	2.15	8.53	0.008	0.992
CR284	cerise rose	*S. l. cera*	10.45	2.80	6.42	0.104	0.896
CR287	Cisterno	*S. l. cera*	21.55	2.35	7.95	0.011	0.989
CR288	Criollo	*S. l. cera*	26.11	3.67	6.83	0.337	0.663
CR291	Pyriforme	*S. l. cera*	10.04	2.03	7.65	0.008	0.992
CR292	8 bis	*S. l. cera*	20.65	2.22	9.05	0.138	0.862
CR293	Costa Rica	*S. l. cera*	15.87	3.17	7.20	0.019	0.981
CR294	Phyra	*S. l. cera*	5.25	2.22	6.70	0.546	0.454
CR296	Poire jaune	*S. l. cera*	16.94	2.23	6.75	0.007	0.993
CR317	Heinz 1706	*S. l. esc*	43.70	2.50	6.12	0.008	0.992
CR321	Edkawy	*S. l. esc*	224.30	11.18	5.30	0.01	0.99
CR341	Cra 66	*S. l. esc*	40.45	5.18	7.10	0.186	0.814
CR354	Stupicke Polni Rane	*S. l. esc*	61.21	4.16	6.05	0.024	0.976
CR359	Muchamiel	*S. l. esc*	172.94	5.54	5.32	0.008	0.992

[a] Accessions are part of *S. l. cerasiforme* (*S. l. cera*), *S. l. esculentum* (*S. l. esc*) or *S. pimpinellifolium* (*S. pimpi*).
[b] Values for fruit weight (FW), locule number (LCN) and soluble solid content (SSC) are adjusted mean from two years of experiment.
[c] STRUCTURE software results are probability of membership in each subpopulation and are based on 20 SSR markers described in Ranc *et al.* 2008
