# Supplementary material for: Genome-Wide Association Mapping in Tomato (Solanum lycopersicum) Is Possible Using Genome Admixture of Solanum lycopersicum var. cerasiforme
Source: G3 (Bethesda). 2012 Aug 1;2(8):853–64. doi: 10.1534/g3.112.002667 (PMC3411241; doi:10.1534/g3.112.002667)
Supplement: Supporting Information [file supp_2.8.853_TableS2.pdf]

Table S2   Description of the 81 DNA fragments sequenced located on chromosome 2

fragment name	F-primer sequence<br>R-primer sequence	Putative Function [a]	Unigene	BAC overgo [b]	Original marker name [c]	genetic position on chr. 2 [d]
TD018	CCGCCGCTCTTTCTTTGCT<br>TTCATGACTCCAGCTGGTC	pyrophosphate-fructose-6-phosphate 1-phosphotransferase beta	SGN-U583285	C02HBa0175O20	-	75.7cM (Expen2000)
TD047	GTTAACGTGGTGTAGGTGCA<br>AAGGTTGCAGGTACCTCTTGA	promoter of homeodomain transcription factor (WUSCHEL), putative	SGN-U589573	C02SLm0057H03; Le_HBa0139K19	-	88.0cM (Expen2000)
TD049	ACGGTCCTAATTGCTAACGCA<br>CTTGGGCCATAATGTAATTGTCT	lactoylglutathione lyase / glyoxalase I, putative	SGN-U578074	C02HBa0167J21	-	72cM (Expen2000)
TD055	AGTTTGAAGCTTCGGTTCTCC<br>TACCTACAGTAGGTGGGTGT	ovate protein	SGN-U582169	C02SLm0128E12	ovate	89.5cM (Expen2000)
TD056	GATTGCGCATTGAGATGCT<br>CGGGGGCAGATACATAGTGA	5' region of fw2.2 gene (Frag 4 : Nesbitt & Tanksley 2002)	no	C02HBa0208N01; BAC clone FW2.2	fw2.2	116cM (Expen2000)
TD083	TAGTGCCGGATCCGCTATG<br>TATTAGTCCCAGCCTTTGCA	sucrose-responsive element binding factor, myb family transcription factor, putative	SGN-U569474	no	cLEX-2-I9	133cM (Expen2000)
TD085	CCAACTTCAACTTGTTTGGGA<br>AGGGACTTCCATAATCATATC	homology with ubiquitin interaction motif-containing protein / LIM domain-containing protein	SGN-U584698	C02HBa0111M10	cLPT-1-A21	114cM (Expen2000)
TD086	GACCAGAGCGTGCTTCTTGA<br>TACCATTCTGGGAGCGGTAT	mitochondrial processing peptidase beta subunit, putative	SGN-U580309	C02SLm0025E22	cLET-5-L17	103cM (Expen2000)
TD088	TAGATGGAGTGGGTATTGTTGA<br>CAAAGTCGGTCTTAACACGACA	-	no	C02SLm0057H03	cTOF-13-F10	86cM (Expen2000)
TD090	ATGGATATGGTAATTGGAGGA<br>CATGAGTTGAGCTTCATGACT	transcription factor, myb-like protein, putative	SGN-U574375	C02HBa0059M17; C02HBa0031A21	T0266	67cM (Expen2000)

fragment name	F-primer sequence<br>R-primer sequence	Putative Function [a]	Unigene	BAC overgo [b]	Original marker name [c]	genetic position on chr. 2 [d]
TD091	TCTTGGCATTCGCACAGGAA<br>CCTGGGAGTTATGGGATCTT	expressed protein similar to UV-B and ozone similarly regulated protein	SGN-U580690	no	TG14	54cM (Expen2000)
TD092	GTAGAGTGTGGGAATATGGAG<br>CCACTAAGCAAAGCTAACCA	-	no	C02HBa0072A04	TG451	46cM (Expen2000)
TD093	TTGGGAGAGGACGAAGGA<br>CTCATCATAAGGCTCTTGCT	expressed protein similar to Glutathione S-transferase	SGN-U583311	C02HBa0320M09	T1698	36cM (Expen2000)
TD094	TTAACGTACTCGTTGCGTGC<br>ATTGGAAATCCCAACAGCCA	CHP-rich zinc finger protein, putative	SGN-U583902	C02HBa0209K17; C02HBa0025A22	cLED-19-B18	27cM (Expen2000)
TD095	GAAGAACATGAGAAGCAGCC<br>AGTTCCTACCCACAAGTATCA	expressed protein	SGN-U583803	C02HBa0163K16	CT140	16cM (Expen2000)
TD096	CAAACTACCCCCAGGTCCA<br>GTATCAATCTCGTCTCGGAGT	cinnamic acid 4-hydroxylase (C4H), putative	SGN-U581122	C06SLe0014B21.1	T0123	5cM (Expen2000)
TD098	CCAAGGCAGAGATAAACGTG<br>CCATGAGGTTCCTACACATC	Rho-GTPase-activating protein-related, putative	SGN-U580990	C02HBa0060J03	T0759	82cM (Expen2000)
TD100	ATCTCTCTGAGGGTTCAAGACAGG<br>TATATCAGCTCCATACTTCTTTGC	expressed protein	SGN-U567423	C02HBa0159F19; C02HBa0030D08	C2-At5g66090	83.2cM (Expen2000)
TD102	TCTGAAGAAGCTGAAGCAAGTAGAGC<br>TGCCAACTGACGAGCATAAGCTGC	expressed protein	SGN-U565307	C02HBa0159F19; C02HBa0030D08	C2_At3g01160	83.4cM (Expen2000)
TD106	CTGGCATGGGATGTAGTGC<br>GATGCAGAAATGTTCGAAGGC	CER6; very-long-chain fatty acid condensing enzyme (CUT1 homology)	SGN-U566767	C02HBa0164H08	T1535	89cM (Expen2000)
TD108	GTGAATTGTCCGGTTCTCGT<br>AATGCTCATCCCTTGTTTGC	expressed protein	SGN-U575206	C02HBa0134G09; C02HBa0011O23	U146494	90cM (Expen2000)

fragment name	F-primer sequence<br>R-primer sequence	Putative Function [a]	Unigene	BAC overgo [b]	Original marker name [c]	genetic position on chr. 2 [d]
TD109	GTCTACCCTGCTGCACAAC<br>CTGTTGAACTGTGGTCTCCA	RAD23; ubiquitin family protein	SGN-U569990	C02HBa0164H08	CT232	90.1cM (Expen2000)
TD110	CACCTCCCAGATCACAATG<br>CATGTGTGGTACATGCTCTG	chlorophyll a/b binding protein CP24 10B, putative; homology to peroxidase 42	SGN-U577555	C02SLm0008E03	T1395	72.0cM (Expen2000)
TD111	CAGTGGTGCTGAAGATGTTG<br>GTAAGCCCTTTAGAGCTCTC	asr; Adenylyl sulfate reductase (homology to APR1)	SGN-U580331	C02HBa0167J21	-	NA
TD112	GCGAGGATAACGGTGAGAAG<br>TGCCTTTGGAGACTCCTTGT	protein kinase, putative	SGN-U563886	CO2Hba0073P13	U153274	120cM (Expen2000)
TD113	TTCATTGATTTCTCCGCTGC<br>TGAACCACCAAGACGGGA	frk3, fructokinase 3	SGN-U570882	CO2Hba0073P13	-	120cM (Expen2000)
TD114	CTGTAAAGAGGGTGCCTAC<br>CGGTACTTGGTTCAAACCTG	expressed protein	SGN-U573852	CO2Hba0073P13	-	120cM (Expen2000)
TD116	CCTGGAATTCGTGCCTTGC<br>GATACCACTAAGTACAGCCTC	oligopeptidase A, putative	SGN-U565226	CO2Hba0073P13	-	120cM (Expen2000)
TD117	GACTCTTCTTTGGTGCTGC<br>CCAACTGCTTCGCTTCTCT	expressed protein	SGN-U598856	CO2Hba0073P13	-	120cM (Expen2000)
TD125	TCATGAGCAACCTGCATATG<br>TAGGCTCAATGTCATTGATCAC	cyclin family protein, similar to cyclin D3.1 protein	SGN-U583476	no	-	2-K(CL) (ILs pennellii)
TD120	ACCCAACACTCTAGCCCAACT<br>ATGGCCGTAATTTTCGTAATCATCA	-	no	Le_HBa0139K19	-	physical map Contig 2
TD121	GTAGTACGTATAGAATGGGTTGT<br>TAATGATGCGGCACTTGCTCA	-	no	C02SLm0057H03; Le_HBa0139K19	-	physical map Contig 2

fragment name	F-primer sequence<br>R-primer sequence	Putative Function [a]	Unigene	BAC overgo [b]	Original marker name [c]	genetic position on chr. 2 [d]
TD129	TGAATTTTGGGAAGTCTGGTT<br>TGCAACTCTACCTCTTTTTCAGC	nuclear matrix constituent protein, putative	SGN-U584279	no	TG151	126cM (Expen2000)
TD130	GGTTTTGACTTGACATGAAG<br>CTTATTCTCCAAACAATTGC	-	no	C02SLm0065M14; C02HBa0016A12	TG518	95cM (Expen2000)
TD132	CGATCGTGCATACTCTCGTG<br>GCTTCTACTGATGATCCTAC	-	no	C02SLe0127J16; C02HBa0159F19; C02HBa0030D08	TG278	81,2cM (Expen2000)
TD133	TTGGGCGACCACGCTGAATC<br>TTACCCACATCAGGACCTTGCC	peptide methionine sulfoxide reductase, putative	SGN-U576565	C02HBa0130B04	C2_At2g18030	83,1cM (Expen2000)
TD137	ACCTAGAGAGGACCTTCCAGAGCCC<br>AGGAATTCAGTGCCTTCAATGCAG	hydantoin utilization protein-related, putative	SGN-U562909	C02SLm0020J02	C2_At4g20070	29cM (Expen2000)
TD138	TTTTCACCAGTAGGAACATTCAAGGTAC<br>CAGGATAATAAACCATCATGCCACAA	Z15141; chitinase (endo-), acidic, 26 kD	SGN-U581507	C02HBa0238L13; C02HBa0013N18; C02SLe0031D11	cLET1A5	79cM (Expen2000)
TD139	ATCCTCTGCCCTTTCTTTCC<br>CAATTGCAGGGGATATGCTT	transport protein, ATPase, gamma chain, chloroplast, putative	SGN-U581255	C02HBa0167J21	T0562	71cM (Expen2000)
TD140	TTCAGGAATGGCATTTGCAAGTGTG<br>ACCATTGAATACAGCATCTGGTCGAAC	aldose 1-epimerase family protein, similar to apospory-associated protein C	SGN-U585203	C02HBa0213A01; C02HBa0030A21	C2_At5g66530	87.5cM (Expen2000)
TD145	GCCAATTTCTAGCGAACATG<br>TCTGTTTCTTCCAACACTAG	histidine kinase family, putative	SGN-U603568	C02SLm0020N19; C02HBa0009K06	TG191	83.30cM (Expen2000)
TD150	GACTCCAGAAGCGATTCTGT<br>CTTCGGCAACTCCTCTTAGC	Cnr; squamosa promoter-binding protein-like 3	SGN-U576708	no	-	60;0cM (Expen2000)
TD187	GCGGAGCTTGAAATCAGTAAC<br>AAGGCACGCATGAGATGATAA	-	no	C02SLm0057H03; Le_HBa0139K19	-	physical map Contig 2

fragment name	F-primer sequence<br>R-primer sequence	Putative Function [a]	Unigene	BAC overgo [b]	Original marker name [c]	genetic position on chr. 2 [d]
TD188	AAACTCACTTACCACTTTCC<br>CTCCTAGTAAGTCTCGATTC	-	no	C02SLm0057H03;<br>Le_HBa0139K19	-	physical map Contig 2
TD265	TGCTCCAAAGTGTGCTCATC<br>AGCGGTACCTTTCTCCTGGT	er60; ethylene-responsive catalase	SGN-U578479	no	T0200	81cM (Expen2000)
TD268	ATTGGCAAATGAGCTTGCTT<br>TTGCAAAAAGAACGGTTTCC	expressed protein	SGN-U585443	C02HBa0213A01;<br>C02HBa0030A21	HBa0030A21	88.5cM (Expen2000)
TD270	AATGCACATGCGTCACAAAT<br>TTGCACCCTCTCATCAACAG	chaperonin, putative (response to oxidative stress)	SGN-U576925	no	cLEM-23-E21	11.0cM (Expen2000)
TD272	CTACCTGGATCGCAATGGTT<br>TTAGGTGCAACAGCATCTCG	peptidyl-prolyl cis-trans isomerase, putative (protein folding)	SGN-U573199	C02HBa0236E02;<br>C02HBa0016A12	T1475	90.5cM (Expen2000)
TD274	GCTAAATCGAATGCCTGAGC<br>GGAAACCGCCAAAACTATCA	integral membrane protein / sugar transporter family protein, putative	SGN-U575594	C02HBa0074A14;<br>C02HBa0030A21	-	physical map Contig 3
TD275	GAGGGTGAGCGATTTATGGA<br>GTCCAGGGATCACAGCATCT	succinate dehydrogenase flavoprotein, putative (nergy pathways; carbohydrate metabolism; citric acid cycle; highly expressed in fruit)	SGN-U580353	C02SLm0128E12;<br>C02HBa0215M12;<br>C02HBa0023N04	-	physical map Contig 3
TD276	AGTTGACGTGTGGCTTACCC<br>CAGGCTTTTTCTCCTTGACG	SIN-like family protein, putative (transcription)	SGN-U572730	C02SLm0128E12;<br>C02HBa0106H06	-	physical map Contig 3
TD278	GCCGAACATGAGAAGGAGAG<br>CAGCTAACCATGACGAGCAA	flavodoxin family protein, putative	SGN-U576263	C02HBa0164H08	-	physical map Contig 3
TD279	AGCTCCTTACAGAGGCAGCA<br>ACCCAAGGGACAGCCTAGTT	contig_ovate16	no	C02HBa0215M12;<br>C02HBa0074A14	-	physical map Contig 3
TD280	ACATCCAAGCATGGGCTAAT<br>TGGGCACACAATGCTTAGAA	contig_ovate22	no	C02SLm0128E12;<br>C02HBa0215M12;<br>C02HBa0023N04	-	physical map Contig 3

fragment name	F-primer sequence<br>R-primer sequence	Putative Function [a]	Unigene	BAC overgo [b]	Original marker name [c]	genetic position on chr. 2 [d]
TD300	TGGATAGCACGTGAAATGGT<br>AATGGAAATCCAGGATCAGC	contig_ovate35	no	C02SLm0097L01;<br>C02HBa0164H08	-	physical map Contig 3
TD304	GTTCATTCTGGGGATGGGT<br>TGCAGCTATCCTTGCTTTG	expressed protein	SGN-U586574	C02HBa0111M10	C2_At1g19690	113.0cM (Expen2000)
TD305	TGGTGAATGGAGAAATGCAG<br>TGATGCCCACTTACACAAGC	expressed protein	SGN-U569081	C02HBa0136C06	C2_At1g20770	99.5cM (Expen2000)
TD316	GATGCTGCCTTATTTGCTC<br>CCATCTCAGGGTGTTTTGT	cellular repressor of E1A-stimulated genes (CREG) family, putative	SGN-U578677	C02SLe0128J14;<br>C02HBa0189G15	C2_At2g04690	97.30cM (Expen2000)
TD328	CCGTTGGTTGGATATTGCTT<br>AAAAGGCACCCCAAAAGAGT	regulator of chromosome condensation (RCC1) family protein, putative	SGN-U565338	C02SLe0092M23;<br>C02HBa0046M08	C2_At3g02300	111.80cM (Expen2000)
TD339	CTCATCTTCAACTTCCCTTCC<br>CATCAACCACTGAGCCAAC	inositol monophosphatase family protein, putative	SGN-U572028	C02HBa0161P02;<br>C02HBa0031A21	C2_At4g05090	69.80cM (Expen2000)
TD343	TCCGCCGTATCTAACCTATC<br>CTGTCCAGTAGTAGCATCCC	transducin / WD-40 repeat protein family, putative	SGN-U565169	C02SLm0114O11;<br>C02HBa0329G05	C2_At4g21520	69.70cM (Expen2000)
TD345	GAAGTGGAAGACCCACAAA<br>CCACTAGAGCCTCCATGTATC	NADPH quinone oxidoreductase-like protein, putative	SGN-U579777	C02HBa0090O01	C2_At4g21580	68.50cM (Expen2000)
TD348	ATTCGCCAGAAATGGATCAG<br>TGGTTGCAACACAATCATCA	expressed protein	SGN-U595227	C02HBa0208N01;<br>C02HBa0012A12	C2_At4g33985	118.50cM (Expen2000)
TD350	GAAAGGAAGCAACCCAATC<br>GCTTAATCCTCGACCAGACA	expressed protein	SGN-U563682	C02SLe0026H18	C2_At4g35560	78.50cM (Expen2000)
TD356	TATGTGGGCAACAAGTCAGC<br>CAAAAAGGAGACCGAACCAA	pyridoxal kinase, putative	SGN-U580571	C02SLe0054B08	C2_At5g37850	119.50cM (Expen2000)

fragment name	F-primer sequence<br>R-primer sequence	Putative Function [a]	Unigene	BAC overgo [b]	Original marker name [c]	genetic position on chr. 2 [d]
TD363	ACCCGTTCAGTCTCACATTTC<br>CCAATGCTATCCACCTTATCC	ribosomal protein L15 family protein, putative	SGN-U583446	C02HBa0144P17	C2_At5g64670	76.00cM (Expen2000)
TD369	TCCTGAGGACATTGGACACA<br>TGGCAGAAACCTCCATTCTT	weak homology with nodulation protein-related	SGN-U570126	BAC clone FW2.2	-	physical map Contig 4
TD373	CAAGCAGCCAAGATCTGTCA<br>TCCCATCTTCAAACCTGGTC	expressed protein	SGN-U581635	C02SLm0132H19; C02HBa0044J01	-	physical map Contig 2
TD374	AAGAGGAGAAGGCCCAGAAG<br>CTTTCTGTGTCCGAGGAAGC	expressed protein	SGN-U563261	C02HBa0044J01	-	physical map Contig 2
TD375	CGCGGTACACCGTCTTTTAT<br>TTCACATTTTCTGGCCTTCC	plastidic fructose-bisphosphate aldolase (photosynthesis; Calvin cycle; carbon fixation)	SGN-U580022	C02SLm0132H19	-	physical map Contig 3
TD376	AAGGGCCTTCAGATGAGGTT<br>CCGATTGCCTCTCTTAGTGC	vesicle tethering family protein, putative	SGN-U582526	C02HBa0074A14; C02HBa0215M12	-	physical map Contig 3
TD377	CAAGACGATGCGAAAGATGA<br>CAGCATTCATGGAATCATGC	-	no	C02HBa0074A14; C02HBa0030A21	-	physical map Contig 3
TD379	TTAAAAGATGGGGCATGAGG<br>ACGTCAAACTGGACCAGACC	-	no	C02SLm0128E12; C02HBa0215M12; C02HBa0023N04	-	physical map Contig 3
TD380	GCCTTGGAAACTCACGAAAG<br>GCGACAATATTTCGGGCTTA	chromatin remodeling complex subunit	no	C02SLm0128E12; C02HBa0215M12; C02HBa0023N04	-	physical map Contig 3
TD381	TTTGTTCCCCTGCGTAAGAG<br>GGGTATTTTAGGCCCTCGTC	-	no	C02HBa0164H08	-	physical map Contig 3
TD382	GCACGCCACGACAGTTACTA<br>ACGTTTTCTGCGCGAGTTAT	homology with retrotransposon Tork11	SGN-U594026	C02SLm0132H19	-	physical map Contig 2

fragment name	F-primer sequence<br>R-primer sequence	Putative Function [a]	Unigene	BAC overgo [b]	Original marker name [c]	genetic position on chr. 2 [d]
TD383	CTCCGTCCCTAGTTGTCCAC<br>CAGGCCATAATCCAAATGGT	acs8; 1-aminocyclopropane-1-carboxylate synthase	SGN-U565888	C02SLm0132H19	-	physical map Contig 2
TD384	CTGCAAGGGCTAGTTCAAGG<br>CGGGAGTGAGGTGTTTGAAT	putative receptor-like protein kinase gene	SGN-U603238	C02SLm0132H19	-	physical map Contig 2
TD385	AACAAAAGCACCACCAAAGG<br>AAAGGAGAGGCTCCGAGTTC	-	no	C02HBa0044J01	-	physical map Contig 2
TD386	TTAACAAGGGCGTGCACATA<br>CCCGTGCAATACCTTGATCT	-	no	C02HBa0044J01	-	physical map Contig 2
TD387	GAAAATGCAGGAGGAAACCA<br>ATGTGAATCCCGATAGCAACA	-	no	C02HBa0130B04;<br>C02HBa0044J01	-	physical map Contig 2

[a] Putative functions of genes are given according to annotation of unigene (http://solgenomics.net/) or manual annotation.
[b] BAC overgo indicates sequence identity with BAC sequences available on genbank.
[c] Name of marker located on the reference map are indicated when available.
[d] Genetic distances are available from the Expen2000 reference map (http://solgenomics.net).
