# Supplementary material for: Genome-Wide Association Mapping in Tomato (Solanum lycopersicum) Is Possible Using Genome Admixture of Solanum lycopersicum var. cerasiforme
Source: G3 (Bethesda). 2012 Aug 1;2(8):853–64. doi: 10.1534/g3.112.002667 (PMC3411241; doi:10.1534/g3.112.002667)
Supplement: Supporting Information [file supp_2.8.853_TableS4.pdf]

Table S4 Polymorphism information

polymorphism name [a]	Allele in reference genotype	Second allele	Upstream sequence	Downstream sequence	Frequence of reference allele		
					S. l. cera (N=63)	*S. l. esc* (N=17)	*S. l. pimpi* (N=10)
lcn2.1-1023	C	T	TACGTATAAT	TAGACAAATA	0.86	0.88	0.70
lcn2.1-1073	A	T	AGTGTGATGG	GATAACGGAT	0.77	0.88	0.40
lcn2.1-1161	G	A	TGATGAAAAT	ACGGATGGAG	0.88	1.00	0.64
lcn2.1-1185	T	-	TGAGCATGAT	GAACGTTATT	0.74	0.88	0.10
lcn2.1-1362	A	G	CAGCCTCATA	TTAAATTACA	0.83	1.00	0.73
lcn2.1-1450	A	-	CAAAATAAAA	TTAGTTTTTC	0.91	1.00	0.90
lcn2.1-1480	A	G	AATTCAAATT	TGTTTAATGT	0.67	0.88	0.09
lcn2.1-1505	A	T	AAATATTTTT	AAAATTTTTT	0.83	1.00	0.82
lcn2.1-1536	G	A	CATATCACGA	AAATATCAGC	0.83	1.00	0.82
lcn2.1-1555	G	A	GCTTAAAATA	TTAATTTCTC	0.78	0.88	0.64
lcn2.1-1565	C	A	GTTAATTTCT	TCAATTTCAA	0.83	1.00	0.73
lcn2.1-1579	A	G	ATTTCAATTT	TTTGTCTTTA	0.83	1.00	0.64
lcn2.1-324	C	T	GTACAAATTA	GTTAACCAGA	0.69	0.88	0.27
lcn2.1-434	T	-	GTTTTTTTTT	GTTTTAAAAA	0.89	1.00	0.64
lcn2.1-53	A	G	TTAAATTAAT	ATTATTTTAA	0.69	0.88	0.09
lcn2.1-60	T	C	AATAATTATT	TAATTCAAAT	0.69	0.88	0.09
lcn2.1-686	T	C	TGGCATGATG	TTACTAATTG	0.60	0.44	1.00
lcn2.1-692	A	G	GATGTTTACT	ATTGGACAAT	0.62	0.44	1.00
lcn2.1-744	-	T	ATTTTTTTTT	GGACATATTT	0.19	0.31	0.00
lcn2.1-904	G	A	GGTTTGAAAT	TTGATGTGTT	0.74	0.88	0.40
lcn2.1-939	G	A	ATGTTTTTCA	AATTTTTTTT	0.78	0.88	0.40
lcn2.1-942	T	A	TTTTTCAGAA	TTTTTTTCGT	0.90	1.00	0.70
lcn2.1-964	G	A	TTCCTTGCTT	TTTTATGTGT	0.76	0.88	0.40
TD018-103	C	T	GAAGCCCTTT	AAAGTCGTTG	0.67	0.92	0.14
TD018-611	T	C	CAAACGCCAA	TAAGGAGGAT	0.78	1.00	0.14
TD047-220	C	T	TTTTTTAATA	GAGGAAATTT	0.73	0.80	0.09
TD047-274	ATTAAATTA ATGAAAGAT AA	----------------- ---	TTTTAAAATT	ATTAAATTAA	0.92	1.00	0.55
TD047-435	A	G	TCATGTAAAT	TTTTAAATAA	0.78	0.85	0.18
TD047-505	C	T	TATAACGATA	TTATAAAGTT	0.80	0.85	0.27
TD047-571	G	A	CATAACGAAC	ATTATTCAAG	0.74	0.85	0.09
TD047-573	---	GAT	AACGAACGAT	TATTCAAGA	0.74	0.83	0.09
TD049-339	C	A	GAGCTCATAT	CCATTGATCA	0.78	1.00	0.45
TD049-348	T	C	TCCCATTGAT	ACATACATGC	0.81	1.00	0.64
TD049-445	T	A	GAAATGAAGT	ATCTTGGTGT	0.81	1.00	0.64

polymorphism name [a]	Allele in reference genotype	Second allele	Upstream sequence	Downstream sequence	Frequence of reference allele		
					S. l. cera (N=63)	*S. l. esc* (N=17)	*S. l. pimpi* (N=10)
TD049-457	A	G	TCTTGGTGTT	AGTCCAGGAA	0.78	1.00	0.36
TD049-528	T	C	TTGACAACCT	CGCGCTTTTG	0.51	1.00	0.09
TD049-96	TCAAATTC	--------	GTTAGAGATG	ACATTGTAAC	0.78	1.00	0.45
TD055-418	A	G	GTACAGCGGG	TATTAAGCGG	0.93	1.00	0.80
TD055-469	T	A	GGAAACTGAA	CTTTAGTTTC	0.80	0.93	0.10
TD056-134	C	T	CTGACCCTCA	TCTCTTTTCT	0.68	0.93	0.09
TD056-155	A	T	TGGCATTATT	TCCAGAAAAG	0.74	0.93	0.27
TD056-252	AC	--	AAAAAACAAT	ACTCATTTAC	0.82	0.93	0.27
TD056-28	T	C	TTCATTTGAT	TGAAAACGAA	0.94	1.00	0.82
TD056-64	-	A	AGAATATTGT	ACGATTATAA	0.94	1.00	0.82
TD056-78	-	T	TTATAATTAG	TAAGCTTAAT	0.94	1.00	0.82
TD056-80	A	T	TATAATTAGT	AGCTTAATTT	0.94	1.00	0.82
TD083-222	C	A	GCCGATTCGA	TCAGTCGAAA	0.82	1.00	0.64
TD083-246	G	T	CCTTTTCAGT	GCTGCTTCCA	0.48	0.81	0.00
TD083-277	---	CCA	TCCTCCACCG	CCACCACCAC	0.92	1.00	0.64
TD083-366	G	C	CCTGATTTTG	GCAAAACTCA	0.97	1.00	0.64
TD083-404	G	T	CAAGGTATTT	TGCCGTTTAG	0.81	0.92	0.09
TD083-666	G	A	TGAATGGGAG	AAGAAACCGC	0.85	1.00	0.27
TD083-685	C	T	GCCTTTGTTT	ATTCTCTCTT	0.81	0.92	0.09
TD086_T7-434	A	G	ATAATCCTCA	TAAAATCTTA	0.88	1.00	0.73
TD086_T7-486	C	T	ACCTATGTTT	ATTGGATTCA	0.88	1.00	0.73
TD086_T7-523	A	G	TAAATAATAC	ATTAGATTTA	0.96	1.00	0.55
TD086_T7-671	G	A	TTAGCATCCA	GGAACTCAAC	0.95	1.00	0.45
TD086_T7-673	G	A	AGCATCCAGG	AACTCAACAA	0.95	1.00	0.45
TD086-312	A	T	AACTCGCCAC	CCCAACTTTA	1.00	1.00	0.64
TD088-204	G	T	AAAGGAAGAC	CTCCTTATTG	0.78	0.88	0.18
TD090-14	A	C	ATTTTCTTGT	TTAATTTATA	0.81	1.00	0.56
TD090-266	-	T	CTCATTTTTT	CTCTATAGGT	0.75	1.00	0.33
TD090-270	C	T	ATTTTTTCTC	ATAGGTTTGG	0.75	1.00	0.22
TD090-306	T	C	TATTTTGTCA	GTTCCTTAGA	0.92	0.92	0.89
TD090-357	C	T	TTTTTTGATT	AGGTGGTCTG	0.75	1.00	0.22
TD090-36	-	A	TTTTTTTTAA	GTTGTATTAT	0.75	1.00	0.33
TD090-625	A	T	TACTTCTCTT	TATCATCCCA	0.75	1.00	0.33
TD091-415	A	-	GGACAAAGGT	AAAAAAAAAA	0.86	1.00	0.30
TD091-607	T	C	TTTTGAGTTT	TAATCATTAC	0.80	1.00	0.10
TD092-450	C	G	ACATTGTATG	TAATTGCATG	0.80	1.00	0.36
TD092-484	A	G	CTTCTAAGGT	TTCAGCGAGA	0.91	1.00	1.00

polymorphism name [a]	Allele in reference genotype	Second allele	Upstream sequence	Downstream sequence	Frequence of reference allele		
					S. l. cera (N=63)	*S. l. esc* (N=17)	*S. l. pimpi* (N=10)
TD092-523	G	A	TTGTATGGAC	TCTCTTTTGA	0.80	1.00	0.27
TD093-148	C	T	AATAGAATCT	TAATAACATT	0.88	0.94	0.09
TD094-128	C	G	TCCCGAACAT	AATTAACTCT	0.80	0.94	0.55
TD094-131	T	C	CGAACATCAA	TAACTCTCAA	0.80	0.94	0.55
TD094-306	T	C	GCGTTATCTG	ATTTTACTCG	0.94	0.94	1.00
TD094-433	G	A	CGCATTTCGG	TGTGTTGCTG	0.94	1.00	0.90
TD094-447	T	C	GTTGCTGATA	TGTATATGAG	0.86	0.93	0.50
TD094-456	A	G	ATTGTATATG	GAAAGGCGGA	0.95	1.00	0.90
TD094-623	ATA	---	AGGTCGATGG	ATAATAATAA	0.89	0.93	0.20
TD094-626	ATA	---	TCGATGGATA	ATAATAATAA	0.94	1.00	0.60
TD095-15	G	A	GGTGAGCATC	TGCTCTGTTC	0.92	0.94	0.91
TD095-249	G	C	TGCTACGTAC	AAATGATAGG	0.84	0.88	0.18
TD095-329	G	A	GAATATGCTG	TAGAGGTGTT	0.97	1.00	0.73
TD096-184	T	C	CAAGAAATGT	GTTTTTGATA	0.89	0.94	0.40
TD096-205	G	T	TTTTTACAGG	AAGGGTCAAG	0.94	0.94	0.40
TD096-259	G	A	GGAAAATGAG	AGGATTATGA	0.89	0.94	0.40
TD096-313	G	T	AGTATAGAGG	GGGTGGGAGT	0.89	0.94	0.40
TD096-328	G	A	GGGAGTCTGA	GCTGCCAGTG	0.92	0.94	0.40
TD096-340	A	T	CTGCCAGTGT	GTTGAGGATG	0.92	0.94	0.40
TD096-376	A	G	AATCTGCTAC	AATGGGATTG	0.93	0.94	0.40
TD096-592	T	C	ACTTGAAGAT	TGTAAGGAGG	0.90	0.94	0.40
TD100-115	T	C	GTTTGCATTT	TTTTTACAAA	0.94	1.00	0.82
TD100-121	C	-	TTTTTTTTTA	AAAAAAGAAT	0.91	1.00	0.64
TD100-172	A	T	ATTCTGAAGT	GATGGTCTGA	0.92	1.00	0.64
TD100-176	---------	TATGACACC	AAGTAGATGG	TCTGATGGAA	0.92	1.00	0.64
TD100-179	C	T	AGTAGATGGT	TGATGGAAAA	0.92	1.00	0.64
TD100-212	A	T	CCATAAATGT	ATATGTGGTG	0.92	1.00	0.64
TD100-213	A	T	CATAAATGTA	TATGTGGTGA	0.92	1.00	0.64
TD100-387	A	G	GCGGCAGGTG	GGAGAGGGGG	0.92	1.00	0.64
TD100-388	G	A	CGGCAGGTGA	GAGAGGGGGG	0.94	1.00	0.82
TD100-569	G	T	TAAATGGGGC	TGCTGATGGC	0.92	1.00	0.64
TD100-68	A	T	ACTTAATCCT	TGGGAATTAT	0.91	1.00	0.64
TD102-263	G	A	CTGGTCTGGC	TTTTCCCAAT	0.72	0.88	0.18
TD106-219	C	T	TGAGAACCCA	AAAGGTGCTG	0.93	1.00	0.55
TD108-347	C	A	AGCTATGTAA	GAAAAATATG	0.80	0.92	0.18
TD109-216	T	C	CTCATCTATA	CAGCAGCTCT	0.82	0.92	0.40
TD109-329	T	C	AAATATTTCT	CGTTTAAGAT	0.82	0.91	0.40

polymorphism name [a]	Allele in reference genotype	Second allele	Upstream sequence	Downstream sequence	Frequence of reference allele		
					S. l. cera (N=63)	*S. l. esc* (N=17)	*S. l. pimpi* (N=10)
TD109-404	G	A	ATGCTTGAGA	TTGAGACTAC	0.82	0.91	0.40
TD109-488	A	G	GAGAATAAGG	CATTAGTGCT	0.84	1.00	0.38
TD109-498	T	C	ACATTAGTGC	TTGCAGAATG	0.82	1.00	0.38
TD110-49	T	G	CTGGAGTTGT	GCCTTGCTAG	0.83	1.00	0.45
TD110-8	T	C	TTGTTCT	GAAGATTTGC	0.84	1.00	0.45
TD112-525	G	T	AATTGATTAG	TAGAGTTTTG	0.85	1.00	0.36
TD113-132	G	T	AGTTCCTCTG	TTTTTTTACT	0.84	1.00	0.18
TD113-230	AGTCTTTCC	---------	CTTTGCTTTC	TCTTCCTAGA	0.97	1.00	0.64
TD114-102	TTAGGAGTA ACAATTG	----------------	TGATTGATTA	TTAGGAGTAA	0.85	1.00	0.36
TD114-151	G	A	AGCAAACAAT	CAAGGAAAGA	0.84	1.00	0.30
TD114-166	C	G	GAAAGAATTA	AGTTGTAATT	0.84	1.00	0.30
TD114-203	G	T	TTTTGCCAGT	CTATTGCCTC	0.84	1.00	0.30
TD114-207	T	A	GCCAGTGCTA	TGCCTCTTAA	0.84	1.00	0.30
TD114-217	--------------	ATTCTTTAA GAGAC	TGCCTCTTAA	AGATCATGGG	0.85	1.00	0.30
TD114-259	G	T	GTTAATCTTT	GTATTTTAAT	0.84	1.00	0.30
TD114-359	G	A	GAACCCGTAT	TGCAATGCTA	0.83	1.00	0.30
TD114-373	A	G	AATGCTAGAT	CACCTATAAT	0.83	1.00	0.30
TD114-392	C	T	ATAGCATGAA	ATCTATATAA	0.83	1.00	0.30
TD114-439	C	T	AATGGTAAAC	TGTAGTTTAC	0.83	1.00	0.30
TD114-464	A	G	TGTAGTGTTA	TGTTGCTTAG	0.85	1.00	0.30
TD114-507	C	T	ATCTTAGGTT	GTTGATAATA	0.83	1.00	0.30
TD114-551	T	C	TGCACCTTAA	GCAGAAATAA	0.86	1.00	0.30
TD114-598	G	T	GCCAAAAGTG	GTCGCGTGCC	0.82	1.00	0.30
TD114-604	G	A	AGTGGGTCGC	TGCCTTTAAT	0.82	1.00	0.30
TD114-613	A	C	CGTGCCTTTA	TGATTTTGTT	0.86	1.00	0.30
TD114-635	T	A	ATTATAGATC	ATGTGGCTTC	0.82	1.00	0.30
TD114-638	G	C	ATAGATCTAT	TGGCTTCTGT	0.82	1.00	0.30
TD114-648	T	C	GTGGCTTCTG	TTCGTAGTGG	0.82	1.00	0.30
TD116-260	G	T	TGGGGCTTTT	GCATCACAAT	0.81	0.91	0.10
TD116-283	C	A	TAATTTTCTA	TAAAATAATA	0.77	0.91	0.10
TD116-355	G	A	TTTTTTCCGT	TCTGTGATTT	0.98	1.00	0.90
TD116-393	G	C	TAAAGATCTC	TCTCTTTCGT	0.94	0.91	0.80
TD116-66	C	G	GTGATTTGAA	GTAGGAGGAG	0.89	1.00	0.40
TD116-707	A	G	GTGTCTTACT	CAAGATTCCC	0.59	0.90	0.00
TD116-745	C	T	TCCTCGGATA	GCTACTACTT	0.88	1.00	0.50
TD117-101	C	T	AAGATTTTTC	GTTGATCTAT	0.95	1.00	0.82

polymorphism name [a]	Allele in reference genotype	Second allele	Upstream sequence	Downstream sequence	Frequence of reference allele		
					S. l. cera (N=63)	*S. l. esc* (N=17)	*S. l. pimpi* (N=10)
TD117-164	T	C	ACTGATTTAT	ATCCTAACCA	0.72	1.00	0.09
TD117-176	T	A	TCCTAACCAT	TTATGGTATG	0.72	1.00	0.09
TD117-219	G	A	GGAGATTCTT	AATTGCTTTT	0.93	1.00	0.91
TD117-399	C	T	AGTTGGGTCA	GTTATGTTTA	0.94	1.00	0.80
TD117-420	G	A	TTGACTCAAA	TCTTCTTTGA	0.94	1.00	0.80
TD117-422	C	A	GACTCAAAGT	TTCTTTGACT	0.94	1.00	0.80
TD117-570	A	C	GATGTACAGT	GCTTATATTC	0.83	1.00	0.13
TD117-623	AC	--	TTGATTTATG	ACACTTTCCA	0.87	1.00	0.30
TD117-672	G	A	AATATGATGT	CGTGTCAAA	0.79	1.00	0.10
TD117-699	C	A	GCCAGTGACG	AGCATACTTC	0.79	1.00	0.10
TD120-172	A	T	TGTTTTTGTC	ATCGATGTAA	0.86	0.94	0.64
TD120-212	T	C	TACTTTTAAA	CGGATAAACC	0.69	0.88	0.18
TD120-283	-	T	TCGTTTGGTT	GGTTTGGTTT	0.68	0.88	0.09
TD120-309	C	T	ATTTTAAAAA	CGACTAGATT	0.89	0.87	0.67
TD120-310	C	T	TTTTAAAAAC	GACTAGATTG	0.87	1.00	0.78
TD120-333	A	T	TTTGGTTTTA	TTTTAATCAA	0.93	1.00	0.56
TD120-382	T	A	CCCCTAAATA	AATACGGATT	0.87	1.00	0.67
TD120-418	A	T	TTTTTTTTTT	AATGGTAATT	0.87	1.00	0.67
TD120-444	-	T	ATTATGGTGT	TTTTTAAAAA	0.67	0.85	0.25
TD120-445	-	T	TTATGGTGTT	TTTTAAAAA	0.71	0.85	0.25
TD120-88	G	T	AAACACTTTG	TCGTGTAATG	0.73	0.88	0.09
TD120-90	C	T	ACACTTTGGT	GTGTAATGAT	0.97	1.00	0.82
TD120-93	G	A	CTTTGGTCGT	TAATGATAAC	0.97	1.00	0.73
TD121-124	C	G	ATTTTTATAA	TATATTATTG	0.82	0.88	0.64
TD121-196	C	A	AAAGACGATT	TGTATTTAAG	0.92	1.00	0.91
TD121-218	T	G	GGAAACGAAC	GAGACCTTTT	0.90	0.88	0.82
TD121-236	A	G	TTTAGTTAAA	ATGAAAAATT	0.68	0.88	0.09
TD121-267	T	A	TCACCACAAA	CCTTGTTGGT	0.77	0.88	0.55
TD121-272	G	A	ACAAATCCTT	TTGGTGCGTC	0.93	1.00	0.64
TD121-278	G	A	CCTTGTTGGT	CGTCATTCAT	0.68	0.88	0.09
TD121-286	C	T	GTGCGTCATT	ATTATTAAAT	0.92	1.00	0.60
TD121-384	A	G	TTTAAAAATT	AACTGTTTAG	0.88	0.87	0.90
TD130-117	T	C	GACAAACATA	TGTAACGAGG	0.82	1.00	0.73
TD130-143	G	C	TTAAGATAGA	ATCAATTCCT	0.95	0.80	1.00
TD130-261	G	T	CATGTAACGA	ACTCAGGGAT	0.98	1.00	0.55
TD130-266	A	G	AACGAGACTC	GGGATTTAAA	0.98	1.00	0.55
TD130-430	C	A	GATCAACATC	ATAAATAAAT	0.77	0.93	0.09

polymorphism name [a]	Allele in reference genotype	Second allele	Upstream sequence	Downstream sequence	Frequence of reference allele		
					S. l. cera (N=63)	*S. l. esc* (N=17)	*S. l. pimpi* (N=10)
TD130-44	A	T	GATCTTAAGC	TGTCATATGA	0.77	0.93	0.09
TD132-122	C	A	GCATCTAATT	CACTCATGAG	0.90	1.00	1.00
TD132-167	G	T	AGGCAAAGAT	TTTGTGGATA	0.65	0.88	0.09
TD132-436	G	A	ACCCTAAAAA	GGGGGAAAGT	0.64	0.87	0.09
TD133-115	----------	CTGCGATTTG	AAAGCCTTTG	TTGGAACATT	0.72	0.88	0.09
TD133-305	T	C	TGGAAGTTAT	GTGTATGATT	0.92	1.00	0.91
TD133-390	T	C	TAAACTAGAG	ATGCAGAATC	0.92	1.00	0.91
TD133-395	A	C	TAGAGTATGC	GAATCCAGGA	0.73	0.87	0.09
TD138-114	T	C	TATTAGCTGC	GTATCTTTAG	0.92	1.00	0.73
TD138-121	T	G	TGCTGTATCT	TAGGGGATGG	0.92	1.00	0.64
TD138-123	A	T	CTGTATCTTT	GGGGATGGCG	0.92	1.00	0.73
TD138-28	G	A	TCGACTTTCC	CTGCAGTATT	0.95	1.00	0.82
TD138-39	C	T	CTGCAGTATT	GGACCCATCC	0.95	1.00	0.82
TD138-48	C	A	TCGGACCCAT	CCACATTCAA	0.94	1.00	0.73
TD138-49	C	T	CGGACCCATC	CACATTCAAA	0.94	1.00	0.73
TD138-50	C	T	GGACCCATCC	ACATTCAAAT	0.94	1.00	0.73
TD138-59	A	G	CCACATTCAA	TTGACCGTTA	0.94	1.00	0.73
TD138-61	T	C	ACATTCAAAT	GACCGTTAAT	0.92	1.00	0.73
TD138-62	G	C	CATTCAAATT	ACCGTTAATG	0.94	1.00	0.73
TD139-547	C	T	TCTCATTTTT	GTGAGTGAAG	0.75	1.00	0.27
TD140-123	C	A	TTGTTGGATA	CAAGTAGGAT	0.84	0.93	0.27
TD140-180	T	A	TATTGAACCT	AATCTGATAT	0.84	0.93	0.27
TD140-480	C	A	TGATCCCGAT	TGGACCAATA	0.08	0.31	0.00
TD145-232	A	G	CTATTACTCA	ATTCGTAAAT	0.66	0.88	0.09
TD145-289	T	C	TGGATCTCAG	AAGGTAAGAT	0.66	0.88	0.09
TD145-317	T	A	AACAACTCTT	TGATGATACT	0.73	0.88	0.27
TD145-328	T	A	TGATGATACT	TTCGAAAGAG	0.72	0.88	0.27
TD145-34	T	C	CGCTCTTCTA	TCTACATCTT	0.66	0.88	0.09
TD145-389	A	G	GAGAGATTAT	GCATAGATGA	0.72	0.88	0.27
TD145-45	A	G	TCTACATCTT	CCTCTGCGAA	0.73	0.88	0.27
TD145-454	A	G	TCATTACTCA	AAGAGTGTTT	0.73	0.88	0.27
TD145-466	A	G	AGAGTGTTTT	ATTAGACCTG	0.72	0.88	0.27
TD145-59	T	C	CTGCGAAAGA	TCATTCTCAG	0.73	0.88	0.27
TD145-9	C	G	ATTCATCA	TCCTTTGTTA	0.73	0.88	0.27
TD145-90	T	A	TAGTTTCTCA	ATGTACATTT	0.66	0.88	0.09
TD150-16	------------	ACAGTTCTTATT	TTTCTGATGA	TTATCCGTGC	0.83	1.00	0.30

polymorphism name [a]	Allele in reference genotype	Second allele	Upstream sequence	Downstream sequence	Frequence of reference allele		
					S. l. cera (N=63)	*S. l. esc* (N=17)	*S. l. pimpi* (N=10)
TD150-303	C	A	CTATGCATAT	ATTTATTTTA	0.85	1.00	0.30
TD150-337	G	C	AAAAGCAAAA	TGAGTCTTTT	0.85	1.00	0.30
TD150-531	---	TTC	TGATGATGAC	TTCTTAGAAT	0.71	1.00	0.10
TD187-41	A	G	TTTATATTTT	AATTTTCTTA	0.78	0.86	0.27
TD187-448	--------	TATAGTAG	AGAATATAGA	TAATATAGTA	0.90	1.00	0.73
TD187-472	G	A	GTTACCACTT	CCACATATAA	0.70	0.86	0.09
TD187-48	C	T	TTTAAATTTT	TTAATGAAAA	0.90	1.00	0.82
TD187-51	A	C	AAATTTTCTT	ATGAAAATTT	0.92	1.00	0.82
TD187-65	-	T	AAATTTACAC	TTTTTTTTAC	0.76	0.86	0.36
TD187-73	-	T	ACTTTTTTTT	ACCTCATTTA	0.76	0.86	0.36
TD188-201	G	C	CTTTTTTCTC	GATGTAAAGA	0.85	1.00	1.00
TD265-456	C	T	AATTACAGAT	GACTACTTCC	0.84	1.00	0.82
TD268-161	T	G	TATTTGTTAC	GTTGCAGTTA	0.90	0.94	0.55
TD268-431	G	C	TTCATCATAT	GTCGGAGGAC	0.86	1.00	0.27
TD270-301	G	A	GCTGTCTACC	AAAATATTTC	0.93	1.00	0.70
TD270-312	A	T	AAAATATTTC	CTCTTGAGGT	0.93	1.00	0.70
TD272-104	A	G	ACAAGGCGAT	AGAGAAGTCC	0.92	1.00	0.82
TD272-277	C	T	AATGGTGGTT	GTATTTTAAC	0.77	0.93	0.56
TD274-17	A	T	AGGTATTTAT	TTGTCTTAGT	0.79	0.94	0.18
TD274-222	C	G	TAAGTATCTA	AGATTGATAA	0.91	0.94	0.50
TD274-325	A	C	GAAAACACTT	CCTTCCTACC	0.83	0.94	0.20
TD274-38	C	T	ACATTTATTT	CGTTTTTTTC	0.95	1.00	0.73
TD275-240	C	T	AAATGCGAGA	CTTTATCTAA	0.89	0.94	0.64
TD276-20	C	T	ATTTTCTTTT	TTTTTCAGTT	0.89	1.00	0.38
TD276-97	T	C	GATGCCAAGA	GTGAGTTACA	0.80	1.00	0.13
TD278-21	G	A	ATCTTTATGA	TACAATCAGA	0.86	1.00	0.90
TD278-267	G	A	GTTAGCCAAC	TTAACCCTGT	0.78	1.00	0.55
TD278-39	A	G	AGAAGGAACG	AGAACTGTAC	0.80	1.00	0.90
TD278-444	A	G	TAGTTAGGTA	CCAACACTAT	0.77	1.00	0.50
TD278-524	C	T	TGTAGACGCT	GATCTTTCTC	0.79	1.00	0.50
TD279-253	C	A	CATTACTAGG	CAAACAAGAA	0.84	0.94	0.55
TD280-328	T	C	CTTTCGTTGG	ATCTAGCGTG	0.49	0.80	0.00
TD300-175	------------	TAATAATAA TAA	AGAGAAATAA	TAATAATAAT	0.81	0.92	0.40
TD300-257	G	A	GACAAAAGCA	AAAGAGAAAA	0.83	1.00	0.33
TD300-41	T	C	CACTCATATT	TAGAAATTTT	0.70	0.92	0.11
TD300-47	A	G	TATTTTAGAA	TTTTAAATTC	0.91	1.00	0.63

polymorphism name [a]	Allele in reference genotype	Second allele	Upstream sequence	Downstream sequence	Frequence of reference allele		
					S. l. cera (N=63)	*S. l. esc* (N=17)	*S. l. pimpi* (N=10)
TD304-235	A	G	CTCTACTGTT	TTGGAAGCTT	0.85	1.00	0.50
TD304-322	T	C	TATGGATCAA	CCTTTCGGAT	0.13	0.07	0.91
TD304-453	A	T	ACATAGTATA	TGAAAACAAC	0.85	1.00	0.45
TD304-514	T	G	CAAACCATGT	TTTATTTAAC	0.85	1.00	0.45
TD304-524	C	T	TTTTATTTAA	TAGGGAAGTG	0.88	1.00	0.60
TD305-236	T	C	GTTTAACAGA	TTGTAATGAT	0.90	0.93	0.40
TD305-355	T	A	AGAAGTTATT	TAGTATTGAC	0.81	0.80	0.10
TD316-112	G	C	ATTGATTGTT	TTGATTTTGC	0.75	0.88	0.11
TD316-143	C	T	ATGACCTAGA	AGATCGAGAT	0.75	0.88	0.11
TD316-197	T	A	TTTTTCTTGT	TCTTGTGTAG	0.77	0.88	0.11
TD316-206	A	G	TTTCTTGTGT	GCACAACTTC	0.77	0.88	0.11
TD316-23	A	T	AGTATGTAGT	GTTCTCTTTT	0.75	0.88	0.20
TD316-256	G	A	GTCTAATTTT	ACCTTGATAT	0.77	0.88	0.11
TD316-259	C	-	TAATTTTGAC	TTGATATGGA	0.77	0.88	0.11
TD316-268	G	A	CCTTGATATG	AGTGTTTAAA	0.77	0.88	0.11
TD316-269	A	C	CTTGATATGG	GTGTTTAAAA	0.77	0.88	0.11
TD316-270	G	T	TTGATATGGA	TGTTTAAAAG	0.77	0.88	0.11
TD316-272	G	T	GATATGGAGT	TTTAAAAGTA	0.77	0.88	0.11
TD316-274	T	A	TATGGAGTGT	TAAAAGTAAA	0.77	0.88	0.11
TD316-275	T	A	ATGGAGTGTT	AAAAGTAAAG	0.77	0.88	0.11
TD316-28	T	C	GTAGTAGTTC	CTTTTTTCTA	0.75	0.88	0.20
TD316-364	T	C	AAAAGTTGGA	TGAAGAGTGC	0.77	0.88	0.11
TD316-407	CAA	---	TTTGAAACAA	ACAAATTGAA	0.87	0.94	0.63
TD316-62	T	G	ATCTTTTACT	CGATTTGTTA	0.75	0.88	0.11
TD316-63	C	T	TCTTTTACTT	GATTTGTTAT	0.78	0.88	0.20
TD316-669	T	A	TATGGATGGA	TGAGATTATC	0.87	0.94	0.63
TD316-77	T	-	TTGTTATTTT	AGTCAACCCC	0.75	0.88	0.11
TD328-318	T	-	AAATGAATGA	GAATTCTCAT	0.91	0.94	0.50
TD328-95	G	T	CTCTATTTCT	ATTTCAATTG	0.90	0.94	0.50
TD339-293	A	C	GATTAATTAG	TAATTTCTCC	0.98	1.00	0.90
TD339-308	G	C	TTCTCCTTCA	TTCCAGTATA	0.98	1.00	0.90
TD339-321	C	T	CCAGTATATA	GACTGTTTGA	0.97	1.00	1.00
TD339-358	T	A	CAAAGTTAAT	TGAAGTGAAT	0.89	1.00	0.90
TD339-389	T	C	GGATTTAACA	AATTTTCAGA	0.89	1.00	0.90
TD339-81	G	A	TGTTGAATTC	TATATTCGAC	0.87	1.00	0.82
TD343-102	-	T	TCTGTTTTTT	CCCTATGTAG	0.71	0.94	0.09
TD343-133	T	-	TATTTTTTTT	CCTGGAATTA	0.86	0.94	0.55

polymorphism name [a]	Allele in reference genotype	Second allele	Upstream sequence	Downstream sequence	Frequence of reference allele		
					S. l. cera (N=63)	*S. l. esc* (N=17)	*S. l. pimpi* (N=10)
TD343-175	G	T	TTCCTCACTT	CTCTGATGAT	0.91	1.00	1.00
TD345-138	A	C	TCGGCAAGGA	GTTACTCGCT	0.94	1.00	0.82
TD345-195	A	T	CCATTTTTTT	AAAAAAGATG	0.89	1.00	0.82
TD345-208	A	T	AAAAGATGTC	ACTTTATAAT	0.94	1.00	0.80
TD345-241	A	T	TTAATTGTTG	TTGTTGTTTA	0.70	0.93	0.09
TD345-253	T	C	TGTTGTTTAT	CTCTTCATGT	0.94	1.00	0.82
TD345-463	TTTTAA	------	AAAGAGATAC	TTTTAATTTT	0.94	1.00	0.80
TD348-300	C	T	GTTAATTTTC	TTATTGAGTT	0.81	1.00	0.22
TD348-71	A	C	TCTTTCAAAG	CTTCCTCTGT	0.97	1.00	0.73
TD348-72	-	T	CTTTCAAAGA	CTTCCTCTGT	0.97	1.00	0.73
TD363-126	T	G	GAGTAAATAT	TCATTTTATA	0.83	0.93	0.90
TD363-170	A	T	GATAGACTAA	GGAATAATTG	0.79	1.00	0.30
TD363-213	C	T	TTTGTTAACG	ATAGAATCAA	0.61	0.93	0.11
TD363-241	A	T	TAGGACATGT	AAATGGGAGT	0.82	1.00	0.89
TD363-486	G	A	CATAGGAGTG	GGTTTTTACC	0.83	0.93	0.89
TD363-498	C	A	GTTTTTACCT	GTGCGCACTC	0.83	1.00	0.89
TD363-499	G	T	TTTTTACCTC	TGCGCACTCA	0.83	0.93	0.89
TD363-517	G	C	TCAAAGGGTA	CAGCTGTGGA	0.96	1.00	0.78
TD363-531	C	T	CTGTGGATTT	CCTTGATGTA	0.83	0.93	0.89
TD363-542	T	A	CCTTGATGTA	AAAAA	0.64	0.93	0.11
TD369-146	A	G	TGTAGGTACA	ATGAATATTG	0.97	1.00	1.00
TD369-23	A	-	GAGTGCTTCC	AAAATCACTG	0.93	1.00	0.91
TD369-328	T	C	TATGCCTTCC	TAGTGAAACT	0.86	1.00	0.20
TD369-340	A	G	AGTGAAACTG	AAAGTTTCAC	0.96	1.00	1.00
TD369-383	G	A	GAGATGATAG	TTTTTGTTAA	0.96	1.00	1.00
TD369-430	T	A	AAAAACTTCT	TATCTTTCAA	0.87	1.00	0.30
TD369-493	G	A	TTATAAGGAA	TCGTTGAGTA	0.77	1.00	0.11
TD373-140	T	C	CCTACATTCT	AAACCTTTTA	0.77	0.86	0.18
TD373-391	G	T	TGTTCTAATT	GGTTGATTAA	0.42	0.50	0.91
TD375-386	TTGAGCTAAT	----------	TTTAGCTAAT	GGATTGAGTG	0.94	1.00	0.90
TD375-573	T	C	ACATCTCTGG	GCAATCCTCT	0.78	0.93	0.30
TD375-98	G	T	CATGTAGTAC	AAGTGTCATA	0.86	0.94	0.70
TD377-91	-	T	GGTGGACTTA	TTTTAAGTAT	0.90	1.00	0.20
TD377-96	A	T	GACTTATTTT	AGTATTTTTT	0.90	1.00	0.20
TD377-97	A	T	ACTTATTTTA	GTATTTTTTT	0.90	1.00	0.30
TD377-98	G	T	CTTATTTTAA	ATTTTTTTTT	0.90	1.00	0.20

polymorphism name [a]	Allele in reference genotype	Second allele	Upstream sequence	Downstream sequence	Frequence of reference allele		
					S. l. cera (N=63)	*S. l. esc* (N=17)	*S. l. pimpi* (N=10)
TD379-180	-	A	AGAATTAAAA	TCATTATTTT	0.90	0.94	0.11
TD379-219	-	T	ATTATTATTA	TATATATACC	1.00	1.00	0.56
TD379-326	C	T	TATTGCAATT	GAAAAGGTTA	0.80	1.00	0.89
TD379-353	A	G	TTCCTATACT	TTCAAAAAGG	0.90	0.93	0.56
TD379-483	A	C	TTTTCCTAAC	CACTCAACCT	0.91	0.93	0.13
TD380-242	C	T	TAACCAACAA	ACCTACAAAC	1.00	1.00	0.82
TD380-256	A	G	TACAAACTAC	AAGTATACTT	0.85	0.94	0.20
TD380-526	T	C	AAAATATAGG	AACTCAGTAA	0.69	0.88	0.00
TD381-516	G	A	TTCAAATCTT	AAAATAAAAA	0.95	0.93	1.00
TD381-548	C	T	GCATAAAGGA	ACAGAATTTT	0.94	0.93	0.50
TD381-568	C	T	TAGAATTTCA	GTATTAATTT	0.94	0.93	1.00
TD381-8	C	T	TTGTCAC	CTTTTGTCTT	0.76	0.93	0.20
TD382-102	C	G	AGACATGCAT	ACTATACTTG	0.93	0.88	1.00
TD382-172	T	C	ACGTGTTTTG	TGATCTTGTG	0.81	0.86	0.44
TD382-251	T	A	ATACATAGAT	TGCCCTTAAA	0.92	0.88	0.82
TD382-37	G	A	TGCATGGACA	ATATGTCCCT	0.79	0.81	0.64
TD383-419	A	G	TACTAGAGAG	GTGTTTTTGT	0.92	1.00	0.33
TD383-558	A	G	AAATTGTATG	ACAAACATTA	0.92	1.00	0.17
TD383-60	A	G	CCATGTGTTT	GCCATAAAAT	0.92	1.00	0.17
TD383-679	T	C	TACAATTTTA	CTTTTAACTT	0.85	0.88	0.60
TD383-684	T	-	TTTTATCTTT	AACTTTAAAA	0.84	0.88	0.60
TD385-209	T	A	TCAAACCAAG	AAAGCATCAA	0.73	0.88	0.09
TD385-613	T	C	GAGAATGTTA	ACATGGATGT	0.74	0.86	0.11
TD386-130	C	G	ATTTAATTAA	AAGTAATTTT	0.88	0.83	0.44
TD386-201	A	G	AGAGAAGCAC	GGTCCTCAAT	0.90	0.83	0.44
TD387-339	G	A	ATCTGCTTTG	TATTTCTTGT	0.93	1.00	0.82
TD387-452	C	T	AACTGTCAAA	CATGTGTAGA	0.78	1.00	0.10

[a] Polymorphism name are described with the 'name of the fragment' dash 'position of the SNP'.
[b] Reference allele is allele of the sequence deposited at GenBank
