# Supplementary material for: Genome-Wide Association Mapping in Tomato (Solanum lycopersicum) Is Possible Using Genome Admixture of Solanum lycopersicum var. cerasiforme
Source: G3 (Bethesda). 2012 Aug 1;2(8):853–64. doi: 10.1534/g3.112.002667 (PMC3411241; doi:10.1534/g3.112.002667)
Supplement: Supporting Information [file supp_2.8.853_TableS5.pdf]

Table S5 Significant associations detected when analysing only the 63 cherry tomato accessions with the MLM model involving either the structure based on SSR markers (Qssr) or the STS markers (Qsnp). Only associations with corrected p-values <0.5 with either model are shown.

		**MLM K+Q (Qssr)**		**MLM K+Q (Qsnp)**	
**trait**	**Locus**	**pvalue**	**corrected p-value**	**pvalue**	**corrected p-value**
**FW**	TD380-526	$4.43X10^{-05}$	0.016	0.0055	ns
	TD056-134	$3.56X10^{-04}$	0.036	0.0026	ns
	TD116-707	$4.07X10^{-04}$	0.037	0.0040	ns
	TD117-219	$2.11X10^{-04}$	0.038	0.0023	ns
	TD138-61	$6.04X10^{-04}$	0.043	0.0425	ns
**LCN**	lcn2.1-692	$9.31X10^{-10}$	$3.43X10^{-07}$	$2.17x10^{-11}$	$8.03x10^{-09}$
	lcn2.1-686	$8.77X10^{-09}$	$1.61X10^{-06}$	$1.66x10^{-10}$	$3.08x10^{-08}$
**SSC**	TD120-90	$8.35X10^{-04}$	ns	$2.44x10^{-04}$	0.044
	TD120-93	$8.35X10^{-04}$	ns	$2.44x10^{-04}$	0.044
	TD380-526	$6.61X10^{-04}$	ns	$4.14x10^{-04}$	0.049

ns: non significant (p > 0.05)
